# Engineered cocultures of iPSC-derived atrial cardiomyocytes and atrial fibroblasts for modeling atrial fibrillation

**DOI:** 10.1126/sciadv.adg1222

**Published:** 2024-01-19

**Authors:** Grace E. Brown, Yong Duk Han, Ashlin R. Michell, Olivia T. Ly, Carlos G. Vanoye, Emanuele Spanghero, Alfred L. George, Dawood Darbar, Salman R. Khetani

**Affiliations:** ^1^ Department of Biomedical Engineering, University of Illinois at Chicago, Chicago, IL, USA.; ^2^ Division of Cardiology, Department of Medicine, University of Illinois at Chicago, Chicago, IL, USA.; ^3^ Department of Pharmacology, Northwestern University Feinberg School of Medicine, Chicago, IL, USA.; ^4^ Department of Physiology and Biophysics, University of Illinois at Chicago, Chicago, IL, USA.; ^5^ Department of Pharmacology and Regenerative Medicine, University of Illinois at Chicago, Chicago, IL, USA.

## Abstract

Atrial fibrillation (AF) is the most common sustained cardiac arrhythmia treatable with antiarrhythmic drugs; however, patient responses remain highly variable. Human induced pluripotent stem cell–derived atrial cardiomyocytes (iPSC-aCMs) are useful for discovering precision therapeutics, but current platforms yield phenotypically immature cells and are not easily scalable for high-throughput screening. Here, primary adult atrial, but not ventricular, fibroblasts induced greater functional iPSC-aCM maturation, partly through connexin-40 and ephrin-B1 signaling. We developed a protein patterning process within multiwell plates to engineer patterned iPSC-aCM and atrial fibroblast coculture (PC) that significantly enhanced iPSC-aCM structural, electrical, contractile, and metabolic maturation for 6+ weeks compared to conventional mono-/coculture. PC displayed greater sensitivity for detecting drug efficacy than monoculture and enabled the modeling and pharmacological or gene editing treatment of an AF-like electrophysiological phenotype due to a mutated sodium channel. Overall, PC is useful for elucidating cell signaling in the atria, drug screening, and modeling AF.

## Teaser

Micropatterned iPSC-atrial cardiomyocyte and fibroblast cocultures are useful to model and treat atrial fibrillation.

## INTRODUCTION

Atrial fibrillation (AF), the most prevalent arrhythmia requiring therapy, affects >33 million patients worldwide (*1*), and the prevalence is projected to double over the next 40 years due to the concomitant rise in risk factors and aging of the population (*2*). Even asymptomatic cases of AF can increase the risk of stroke, myocardial infarction, and heart failure (*3*). Despite recent advances in catheter-based therapies, antiarrhythmic drugs (AADs) are still often used to treat symptomatic AF. However, the response in an individual patient is highly variable with ~50% of patients experiencing AF recurrence within 6 months of treatment (*4*). The lack of AAD efficacy is, in part, due to the inability to target the underlying and often unknown genetic mechanisms of AF (*5*) given the paucity of model systems. While some murine models develop spontaneous AF (*6*), they lack several important genes, including key potassium channels associated with human AF. While primary human atrial cardiomyocytes (CMs) mitigate the species-specific limitations with animal cardiac physiology, several constraints exist with the use of these cells for disease modeling and drug screening including severe sourcing limitations and a precipitous functional decline in vitro (*7*). In contrast, human induced pluripotent stem cells (iPSCs) provide for a nearly unlimited source of cells that can be differentiated into atrial CMs (iPSC-aCMs), which retain the patient’s genome. However, current differentiation protocols lead to immature iPSC-aCMs (*7*, *8*) that do not faithfully recapitulate an AF susceptible cellular substrate. Therefore, there is a critical need to develop improved maturation strategies for iPSC-aCMs with the goals of elucidating the key genetic determinants of AF and developing precision therapeutics (*4*).

While CMs account for the largest volume in the heart, CFs make up ~45 to 55% of the total cell number (*9*) and can modulate CM functions through paracrine signaling, direct contact, and extracellular matrix (ECM) deposition (*10*). For instance, CFs express voltage-gated sodium channels and gap junctions that modulate CM electrophysiology and allow for the propagation of mechanical stimuli (*11*, *12*). Because of these interactions, coculture with CFs can influence CM, and specifically iPSC-CM, maturity (*13*). It has been previously shown that modulating the interaction between iPSC-CMs and CFs yields improvements in sarcomere organization, contraction force, and electrical parameters (*14*, *15*).While atrial CFs (ACFs) are known to differ from ventricular CFs (VCFs) in the levels of ECM proteins, responses to key growth factors, and proliferation rates (*11*), the effects of chamber-specific fibroblasts on iPSC-aCM maturation remain unelucidated.

The enhancement of structural maturity driven by homotypic interactions also improves the metabolic and electrophysiological (EP) state of CMs (*14*, *16*). Thus, ventricular CMs are often spatially oriented using technologies such as micro-contact printing, microgroove structures, and microfluidic chambers into in vivo–like rod shapes and anisotropic linear structures, which has been previously shown to improve sarcomeric activity, myofibril alignment, EP properties, calcium kinetics, and mitochondria distribution (*12*, *17*, *18*). However, it is difficult to scale up these techniques into multiwell plates, which remain the workhorse for high-throughput screening of industrial chemicals and drugs (*19*, *20*). The effects of ECM patterning on iPSC-aCMs remain unelucidated.

Here, we report for the first time that primary adult atrial, but not ventricular, fibroblasts induced greater functional maturation in iPSC-aCMs, potentially due to key molecular pathways as elucidated via RNA sequencing (RNA-seq) analysis and small interfering RNA (siRNA) knockdown studies. We coupled this finding with soft-lithographic patterning of ECM proteins within industry-standard multiwell plates amenable to high-throughput drug screening to develop patterned coculture (PC) of iPSC-aCMs and atrial fibroblasts that significantly enhanced iPSC-aCM structural, EP, contractile, and metabolic maturation for 6+ weeks compared to conventional (randomly distributed) mono- and coculture. Last, we showed that PC has greater sensitivity than conventional culture for detecting drug efficacy and can be used to elucidate the effects of genetic mutations on AF and treat the AF-like phenotypes via gene editing and drug therapy.

## RESULTS

### Effects of atrial or VCFs on iPSC-aCMs

The iPSC-aCMs were cocultured at a 1:1 ratio with human primary adult ACFs or primary adult VCFs. While both CF types improved iPSC-aCM sarcomere alignment and decreased cell circularity relative to randomly distributed iPSC-aCM monocultures (RMs) (fig. S1, A and C), only ACFs (i) increased iPSC-aCM sarcomere lengths (1.79 ± 0.11 μm for iPSC-aCM/ACF compared to 1.58 ± 0.12 μm for iPSC-aCM/VCF) closer to that of adult CMs (2.2 μm) (fig. S1D) (*17*) and (ii) significantly up-regulated *SCN5A* (~2.5-fold for iPSC-aCM/ACF compared to ~1.6-fold for iPSC-aCM/VCF) and *KCNJ3* (~4.3-fold for iPSC-aCM/ACF compared to ~2-fold for iPSC-aCM/VCF) expression compared to RM (fig. S1B). The purity of the ACFs and VCFs (two donors each, commercially sourced from Lonza), as assessed via collagen 1A1 and vimentin immunostainings, was >95% (fig. S1E). ACFs were found to grow faster than VCFs in monocultures (fig. S1F), as also previously described (*21*). Furthermore, both fibroblast types expressed discoidin domain receptor 2 (DDR2), a marker enriched in CFs (fig. S1G) (*22*). Last, we used two donors each of ACFs and VCFs and observed similar maturation effects on iPSC-aCMs for each fibroblast type and each donor for at least six passages (fig. S1, C and D).

To further characterize the various populations of CFs, RNA-seq was performed on the two donors of each fibroblast subpopulation. On the basis of principal components analysis (PCA), it was determined that principal component 1 (PC1) was able to separate ACFs from VCFs effectively. When graphing PC1 versus PC2, ACFs clustered closer together, while PC1 versus PC3 resulted in improved VCF clustering, indicating that the variance associated with these populations can be defined by these principal components (fig. S1H). We found 178 and 635 genes to be differentially up-regulated in ACFs and VCFs, respectively (fig. S1I). While connexins 43 (Cx43) and 45 (Cx45) were similarly expressed, Cx40, an atrial-specific connexin, was up-regulated in ACFs (fig. S1J), which may allow ACFs to form gap junctions with the Cx40 in iPSC-aCMs for electrical propagation (*23*). In addition, ephrin B1 (*EFNB1*), a ligand of Eph-related receptor tyrosine kinases that regulates the maintenance of rod-like shaped CMs and sarcomere alignment (*24*), was up-regulated in ACFs over VCFs.

### Soft-lithographic process to pattern ECM within multiwell plates

**Fig. 1.**
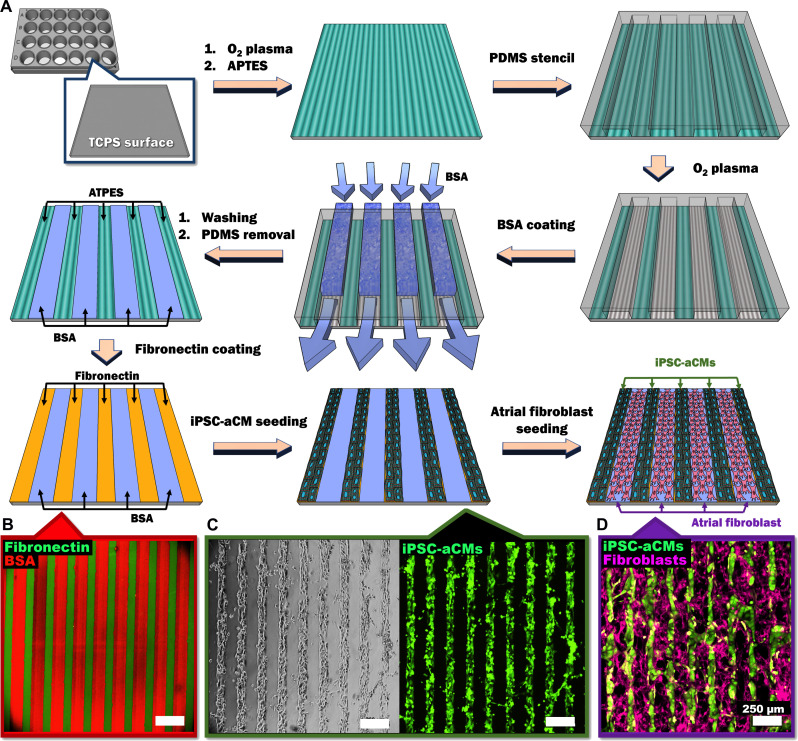
Soft lithographic process to generate PC of iPSC-aCMs and CFs within multiwell plates. (**A**) Tissue culture polystyrene (TCPS) is first treated with oxygen plasma, followed by APTES. Next, a PDMS mask/stencil is placed atop the surface followed by oxygen plasma treatment to etch the unprotected APTES. BSA is then loaded into the channels created between the TCPS and PDMS mask via vacuum force to passivate the surface for CM attachment. The PDMS mask is then removed, and FN is added to create alternating lines (**B**) with BSA. Seeded CMs preferentially attach to the FN patterns as seen via (**C**) phase contrast and calcein AM. Seeded fibroblasts attach to the BSA-coated areas to create the (**D**) PC. Green color and purple color indicate the patterned iPSC-aCMs and atrial fibroblasts, respectively. Scale bar, 250 µm.

A polydimethylsiloxane (PDMS) mask was applied to (3-aminopropyl)triethoxysilane (APTES)–coated tissue culture polystyrene (TCPS) wells of a multiwell plate; then, oxygen plasma was used to ablate the APTES regions unprotected by the PDMS mask, followed by bovine serum albumin (BSA) coating of the ablated regions (80 μm in width) (Fig. 1A). The presence of the APTES patterns was visualized via covalent binding of APTES to sulfo-*N*-hydroxysuccinimide ester–biotin followed by binding of biotin to Alexa Fluor 488–streptavidin (fig. S2). Fibronectin (FN) adhered to the patterned APTES regions (55 μm in width), which resulted in alternating lines of FN and BSA (Fig. 1B). As iPSC-aCMs selectively attached to the FN lines, linear iPSC-aCM micropatterns were created (Fig. 1C). CFs were able to attach to the BSA-coated regions, filling the space between the iPSC-aCM micropatterns (Fig. 1D). The iPSC-aCM/ACFs micropatterns displayed a homogeneous patterning quality throughout the patterning area (8 mm by 8 mm) (fig. S3A).

### Improved structural maturation of iPSC-aCMs in PC

**Fig. 2.**
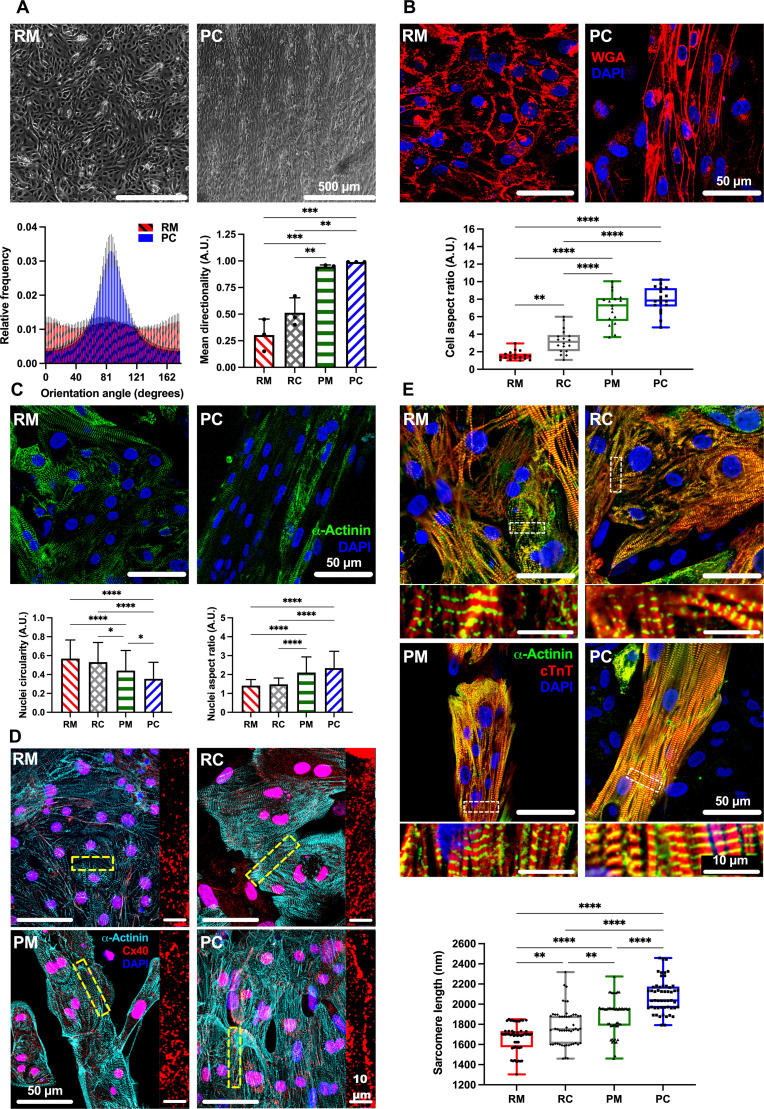
Structural maturity of iPSC-aCMs. (**A**) Phase contrast images of iPSC-aCM RM and PC of iPSC-aCMs and primary adult ACFs showed improved linear orientation in PC, quantified in histogram (bottom left). When compared to RC and PM, RC increased mean directionality of iPSC-aCMs (bottom right); however, iPSC-aCMs in PM and PC showed nearly perfect directionality (*n* = 3 images). A.U., arbitrary units. (**B**) Wheat germ agglutinin (WGA) membrane stain showed improvement in iPSC-aCM AR in PC versus RM; in addition, PM and PC improved iPSC-aCM AR versus RM and RC (*n* = 18 cells). (**C**) Cell nuclei and α-actinin staining identified iPSC-aCMs. Nuclei circularity (bottom left) of iPSC-aCMs showed a decrease in PC versus RM, while the nuclei AR of iPSC-aCMs (bottom right) was increased in PC versus RM, indicating a more elongated phenotype (*n* = 85, 57, 74, and 132 nuclei for RM, RC, PM, and PC, respectively). (**D**) Connexin 40 (Cx40) staining localized around α-actinin–positive iPSC-aCMs was improved in PC versus RM. Right images are magnified regions as indicated by the dashed rectangles in the corresponding left images. (**E**) Culture platforms immunostained for cardiac troponin T (cTnT) and α-actinin were used to visualize sarcomere organization and to quantitate sarcomere length. PC had the longest sarcomere length versus other platforms (bottom) (*n* = 53 sarcomeres). Bottom images are magnified regions as indicated by the dashed rectangles in the corresponding top images. *P < 0.05, **P < 0.01,***P < 0.001, and ****P < 0.0001. (A) Scale bar, 500 µm; [(B) and (C)] Scale bar, 50 µm; and [(D) and (E)] Scale bar, 50 µm (10 µm for magnified images).

iPSC-aCMs in RM were randomly distributed, but these cells were unidirectionally aligned along the micropattern direction in PC, as indicated by the distribution of the PC orientation angle concentrated near 90° (Fig. 2A and fig. S3, B and C). The overall degree of directionality of iPSC-aCMs was also evaluated with 0 indicating full randomness to 1.0 indicating perfect alignment. The directionality of RM, random iPSC-aCM/ACF coculture (RC), patterned iPSC-aCM monoculture (PM), and PC were 0.30 ± 0.15, 0.51 ± 0.14, 0.95 ± 0.02, and 0.99 ± 0.00 (*n* = 3 images each), respectively, indicating near perfect alignment of iPSC-aCMs in PC. To assess PC patterning fidelity over time, both dispersion angle and mean directionality were assessed. Directionality was found to be ~1 over the 21-day culture period with no significant changes. In contrast, a significant increase in dispersion angle was found between day 2 and day 9 of culture (~12° to ~20°), suggesting some remodeling of PC. However, the dispersion angle was maintained at statistically similar values for the remaining culture duration (fig. S3, D and E). Last, ACFs were treated with mitomycin C (1 μg/ml) before coculture with iPSC-aCMs, which enabled slower growth (but not complete arrest at this concentration of mitomycin C) of the ACFs, thereby mitigating fibroblast overgrowth and peeling of the cocultures over time as observed with nontreated fibroblasts. The positive effects of the mitomycin C–treated ACFs on iPSC-aCMs were statistically similar to those of nontreated ACFs (fig. S4). Thus, mitomycin C–treated fibroblasts were used for all subsequent experiments to mitigate the abovementioned issues with the overgrowth of untreated ACFs in cocultures.

In RM, iPSC-aCMs showed rounded cell shapes with an aspect ratio (AR) of 1:1.55 ± 0.49; RC showed an AR of 1:3.21 ± 1.35; PM showed an AR of 1:6.99 ± 1.84; and PC showed an AR of 1:7.92 ± 1.46 (*n* = 18 cells) (Fig. 2B). iPSC-aCMs in RM showed circular nuclei, while iPSC-aCMs in PC showed unidirectionally aligned and elliptical nuclei (Fig. 2C), a known effect of cell elongation on nuclear shape (*17*). Overall, nuclei circularity was found to be significantly decreased in both patterned conditions, PM and PC, and the nuclei AR was significantly increased compared to randomly distributed conditions, RM and RC. In addition to alignment, significant increases in tissue thickness were also observed in PC, while RM remained as a thin monolayer (fig. S5). In addition, multinucleation, a maturation hallmark reflecting CM escape from the cell cycle (*17*, *25*), was observed in iPSC-aCMs within PC at a higher rate than in RM (fig. S6). Last, while iPSC-aCMs in RM exhibited randomly dispersed Cx40 protein staining, Cx40 was clustered along iPSC-aCM boundaries in PC (Fig. 2D). In addition, we also observed Cx40 present at the interface between iPSC-aCMs and ACFs in PCs (fig. S7), suggesting heterotypic contacts.

While iPSC-aCM sarcomeres in RM and RC showed a disordered and sparse distribution as assessed by cardiac troponin T (cTnT) and α-actinin staining, iPSC-aCM sarcomeres in PM and PC showed a highly ordered and aligned shape in the micropattern direction with uniformly spaced and organized z-bands (Fig. 2E). The sarcomere length increased in the order of RM (1670.00 ± 13396 nm), RC (1775.85 ± 182.91 nm), PM (1884.17 ± 175.96 nm), and PC (2083.23 ± 163.40 nm) (*n* = 53 sarcomeres). The iPSC-aCM sarcomere length in PC was similar to the ~2.2-μm sarcomere length in adult CMs in vivo (*17*). Last, iPSC-aCMs were seeded onto linear patterns of either 55 or 85 μm widths and then surrounded by ACFs to generate PC; the iPSC-aCMs did not display significantly different sarcomere lengths nor circularity when comparing the two pattern widths in PC (fig. S8).

### Improved functional maturation of iPSC-aCMs in PC

**Fig. 3.**
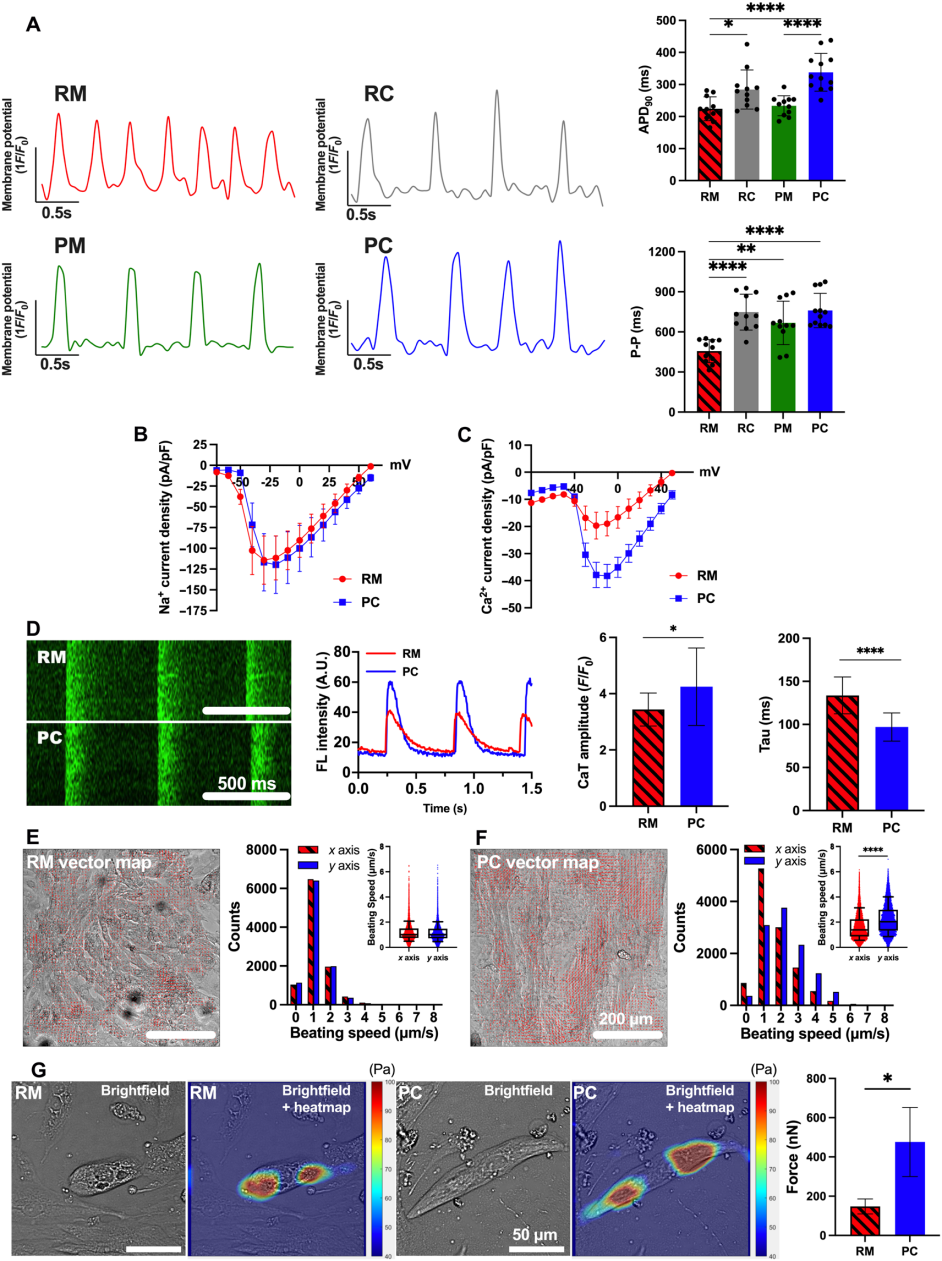
Electrical and contractile maturity of iPSC-aCMs. (**A**) OVM traces of RM, RC, PM, and PC. APD_90_ was increased in RC and PC versus RM and PM (top right); P-P duration was increased (bottom right) in RC, PM, and PC versus RM (*n* = 12 cells). Current density for (**B**) sodium (PC: *n* = 10 cells; RM: *n* = 8) and (**C**) calcium (PC: *n* = 44; RM: *n* = 12) for iPSC-aCMs in RM and PC was measured via SyncroPatch-384, with PC showing increased calcium current density. Error bars represent SEM. (**D**) Time-series line-scan images (left) of calcium flux (Fluo-4/AM dye) and quantification (middle) in iPSC-aCMs, with PC showing higher amplitude (peak signal *F*/baseline signal *F*_0_) and lower tau versus RM (right) (four first beatings of four different cells). (**E**) Left image depicts registered vector field map; right histogram shows similar distribution of beating speed between *x*- and *y*-axis components (inset shows direct *x* and *y* comparison) for iPSC-aCMs in RM. (**F**) The *y*-axis contraction of iPSC-aCMs in PC was stronger than *x*-axis contraction. (**G**) Traction force microscopy on fluorescent bead-laden 25-kPa hydrogels was used to quantify iPSC-aCM contraction force. Bright-field images and its overlay images with traction heat maps (left) for iPSC-aCMs adhered to hydrogel surfaces and previously cultured in RM or PC. Contraction force of iPSC-aCMs in PC was higher (right) compared to RM (*n* = 6 cells). *P < 0.05, **P < 0.01, and ****P < 0.0001. (D) Scale bar, 500 ms; [(E) and (F)] Scale bar, 200 µm; and (G) Scale bar, 50 µm.

We observed an increase in action potential duration (APD_90_) in the order of RM (224.13 ± 37.18 ms), PM (233.53 ± 31.22 ms), RC (284.15 ± 60.94 ms), and PC (337.83 ± 58.97 ms) as assessed by optical voltage mapping (OVM; *n* = 11 cells) (Fig. 3A). Overall, the APD_90_ in PC was closer to the APD_90_ previously measured in primary adult aCMs (~400 to 450 ms) (*26*). Likewise, we observed an increase in peak-to-peak (P-P) duration in the order of RM (456.66 ± 85.83 ms), PM (667.38 ± 162.60 ms), RC (748.15 ± 134.74 ms), and PC (761.45 ± 134.78 ms) as assessed via OVM (*n* = 12 cells for RM, RC, and PM; *n* = 13 cells for PC). Results with automated patch clamp recording showed that while sodium current in iPSC-aCMs from PC was not significantly different compared to RM (Fig. 3B), the calcium current density in iPSC-aCMs from PC was higher compared to RM (Fig. 3C). Last, we used a fluorescent calcium reporter dye, Fluo-4 acetoxymethyl ester (Fluo-4/AM), to evaluate calcium transients (CaTs) in PC and RM. The iPSC-aCMs in PC showed significantly higher CaT amplitude ratio (*F*/*F*_0_) and a shorter signal decay constant (tau) compared to RM (Fig. 3D and movies S1 and S2). Similar CaT analysis trends as above were also observed in another iPSC-aCM donor (fig. S10C).

In RM, individual groups of beating iPSC-aCMs displayed low synchrony (movie S3), whereas iPSC-aCMs in PC displayed a synchronized contraction motion (movie S4). In addition, while iPSC-aCM contraction in RM did not show a dominant direction, iPSC-aCMs in PC showed an anisotropic direction aligned with the longitudinal micropattern. For quantitative analysis of directionality in contraction motion of iPSC-aCMs, a video-based beating motion analysis using a Motion-GUI MATLAB code was performed as previously described (*27*); the algorithm segments the time-series Tagged Image File Format (TIFF) images of cells’ beating motion into tens of macroblocks and then extracts the motion vector information of each segment into a vector map. Time-series TIFF images (2048 × 2048 pixels) of iPSC-aCMs were segmented into 64 × 64 macroblocks; then, the *x* and *y* components of the acquired vector field of segmented images were further extracted and plotted into vector field maps and histograms. The histogram for iPSC-aCMs in RM showed no difference between the *x* and *y* directions for one iPSC-aCM donor (Fig. 3E and movie S5) and slightly stronger contraction in the *y* over the *x* direction for another iPSC-aCM donor (fig. S10D). In contrast, iPSC-aCMs in PC showed significantly stronger contractions in the *y* over the *x* direction for both iPSC-aCM donors (Fig. 3F, fig. S10E, and movie S6) (*27*).

To assess contractile force more directly than possible with the abovementioned video-based beating motion analysis, we performed traction force microscopy (TFM) on iPSC-aCMs from RM and PC using commercially sourced multiwell plates containing soft hydrogels (25-kPa Young’s modulus) with embedded red fluorescent microbeads. The matured iPSC-aCMs were dissociated from PC and RM and replated onto the hydrogel-bead containing plates. The bead displacement from beating iPSC-aCMs (movies S7 and S8) coupled with the known Young’s modulus of the hydrogel was used in a published MATLAB code (*28*) to quantify the contractile force of iPSC-aCMs. The iPSC-aCMs exhibited a higher contractile force in PC (476.1 ± 176.2 nN) compared to RM (147.6 ± 38.21 nN, *n* = 3 cells for each platform) (Fig. 3G). Similar trends were observed in another iPSC-aCM donor, albeit statistical significance was not reached (fig. S10F), likely due to the highly invasive procedure of dissociating the iPSC-aCMs from each platform, replating onto the hydrogel surfaces, and then waiting ~7 days for cell beating to initiate again; such a protocol may cause lower contractility overall than before dissociation. Nonetheless, the video-based beating motion analysis and TFM overall indicate that iPSC-aCMs in PC display stronger contractions and force of contraction compared to iPSC-aCMs in RM.

**Fig. 4.**
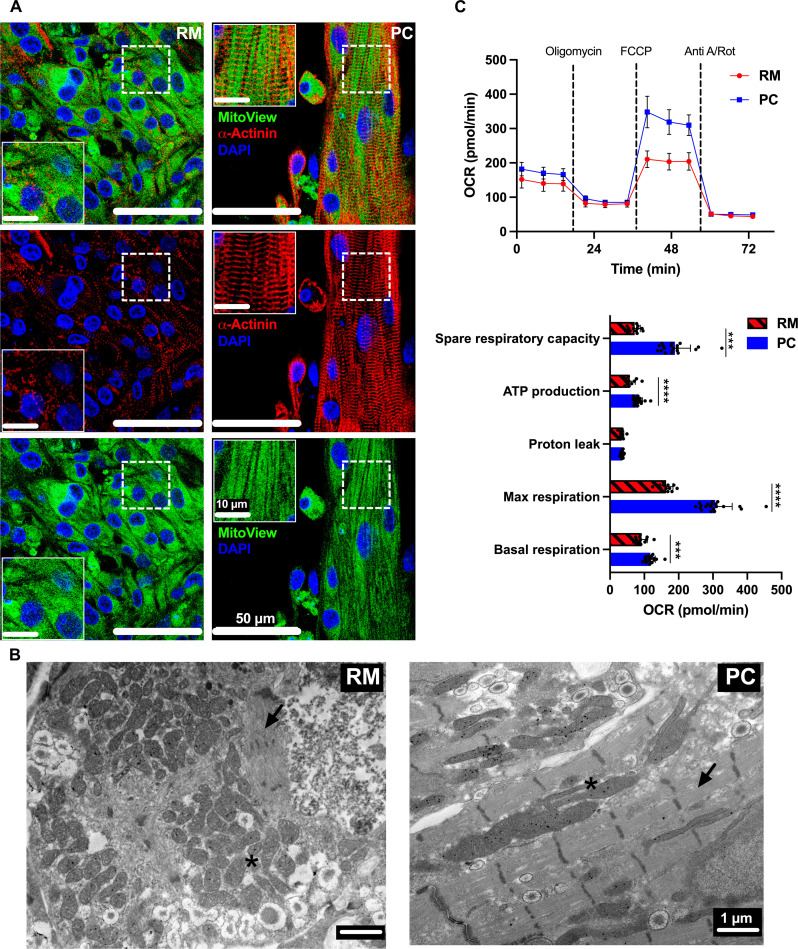
Metabolic maturity of iPSC-aCMs. (**A**) Immunostaining for mitochondria via MitoView and sarcomeres via α-actinin showed that while the iPSC-aCM mitochondria were randomly distributed in random monoculture (RM), they were linear and aligned with the sarcomeres in PC. Inset images are magnified regions as indicated by the dashed rectangles in the corresponding larger image. (**B**) TEM images of iPSC-aCMs in RM and PC, with the latter containing elongated mitochondria (*) and aligned sarcomeres (arrow). (**C**) Seahorse analysis measuring OCR of iPSC-aCMs from RM and PC; additional parameters were calculated from this data. PC showed a significant increase in spare respiratory capacity, ATP production, max respiration, and basal respiration compared to RM (PC: *n* = 20 wells; RM: *n* = 12 wells). ***P < 0.001 and ****P < 0.0001. FCCP, carbonyl cyanide (trifluoromethoxy) phenylhydrazone; anti-A/Rot, antimycin A/rotenone; DAPI, 4′,6-diamidino-2-phenylindole. (A) Scale bar, 50 µm (10 µm for inset images) and (B) Scale bar, 1 µm.

While the mitochondria in iPSC-aCMs in RM were randomly distributed, the iPSC-aCM mitochondria in PC maintained dense and sharp lines along the direction of the sarcomeres (Fig. 4A). We also used transmission electron microscopy (TEM) to visualize at higher magnifications the sarcomeres and mitochondria in iPSC-aCMs within RM and PC; iPSC-aCMs in PC displayed elongated, densely packed mitochondria aligned with organized sarcomeres, whereas the mitochondria in iPSC-aCMs in RM were more circular and randomly distributed (Fig. 4B). The results in PC are consistent with adult CMs containing mitochondria near and along the myofibril structures (*16*). In addition, the oxygen consumption rate (OCR) was analyzed using the Seahorse Extracellular Flux Analyzer (*27*, *29*). The iPSC-aCMs in PC showed significantly greater spare respiratory capacity, adenosine 5′-triphosphate (ATP) production, maximum respiration, and basal respiration compared to RM, while proton leak was similar across the two models (Fig. 4C), which is a hallmark of maturation as adult CMs display a primary energy switch from glycolysis to oxidative phosphorylation (*30*). However, we did not detect significant changes in the levels of several proteins of the electron transport chain (complex I: NDUFB8, complex II: SDHB, complex III: UQCRC2, complex IV: COXII, and complex V: ATP5A) via Western blotting (WB) of PC and RM (fig. S11A). Therefore, the staining, TEM, OCR, and WB results indicate in totality that the greater metabolic maturation of iPSC-aCMs in PC compared to RM may be due to higher ATP production from oxidative phosphorylation within mitochondria with improved morphology and more effective utilization of that ATP by the sarcomeres in close proximity/alignment to the elongated mitochondria. Last, another donor of iPSC-aCMs also showed greater structural, connexin (40 and 43) junction formation, EP, and metabolic maturation in PC compared to RM, thereby showing the utility of the PC platform to different iPSC lines (figs. S9, S10, and S11).

### Improved gene expression patterns of iPSC-aCMs in PC

**Fig. 5.**
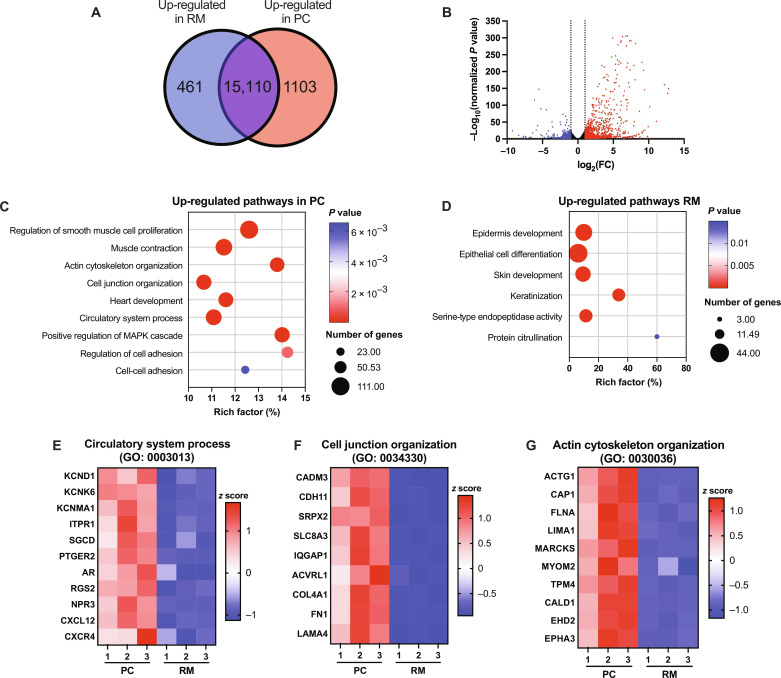
RNA-seq analysis of iPSC-aCMs. (**A**) Venn diagram depicting 461 genes differentially up-regulated in iPSC-aCMs from RM and 1103 genes up-regulated in iPSC-aCMs from PC; the iPSC-aCMs were purified from PC via magnetic activated cell sorting before sequencing. (**B**) Volcano plot showing the distribution of differentially expressed genes in PC compared to RM (red: up-regulated in PC relative to RM, and blue: downregulated in PC relative to RM or up-regulated in RM relative to PC). (**C**) up-regulated GO pathways in PC related to mature cardiac functions. (**D**) Up-regulated GO pathways in RM related to fetal and epidermis development. Down-selected genes up-regulated in PC in key cardiac pathways including (**E**) circulatory system process (GO: 0003013), (**F**) cell junction organization (GO: 0034330), and (**G**) actin cytoskeleton organization (GO: 0030036) (*n* = 3 sequencing replicates).

RNA-seq analysis revealed that the expression levels of 1103 and 461 genes were up-regulated in iPSC-aCMs from PC and RM, respectively (Fig. 5, A and B). Gene Ontology (GO) pathways up-regulated in iPSC-aCMs from PC included muscle contraction, actin cytoskeletal organization, cell junction organization, heart development, circulatory system process, and cell-cell adhesion (Fig. 5C), whereas pathways up-regulated in iPSC-aCMs from RM included epithelial cell differentiation, epidermis development, and serine-type endopeptidase activity (Fig. 5D). In the circulatory system process pathway, 76 genes were up-regulated in iPSC-aCMs from PC compared to RM, such as potassium channel genes (*KCND1*, *KCNK6*, and *KCNMA*), cardioprotective/anti-apoptotic factors (*RGS2* and *NPR3*), and the CXCL12-CXCR4 signaling pathway (Fig. 5E) that is critical for cardiac development and calcium homeostasis (*31*). For cell junction organization, 86 genes were up-regulated in iPSC-aCMs in PC compared to RM including genes related to cell-cell adhesion such as *CADM3* and genes from the cadherin family including *CDH11* (Fig. 5F). Furthermore, gene expression of ECM proteins, including *COL4A1*, *FN1*, and *LAMA4*, were up-regulated in iPSC-aCMs in PC compared to RM. It was previously shown that collagen 4 and laminins are integral parts of the CM basement membrane, while FN was implicated in cell adhesion and as an integral part of cardiac tissue development (*32*). Last, 76 genes were up-regulated in iPSC-aCMs in PC compared to RM related to actin cytoskeletal organization (Fig. 5G), such as *ACTG1*, *CAP1*, *FLNA*, *LIMA1*, and *MARCKS*. In addition, actin-myosin–related binding proteins were also up-regulated in iPSC-aCMs in PC compared to RM including *MYOM2*, *TPM4*, and *CALD1*, which may underlie the improved iPSC-aCM contraction force in PC. Additional genes related to cell cycle arrest (*CDKN2A* and *CDKN2B*), and additional integrins and cytoskeletal proteins (*ITGA*, *ITGB1*, *LIMS1*, *FERMT2*, *TLN1*, and *VCL*) were also increased in iPSC-aCMs in PC compared to RM (fig. S12).

### Effects of Cx40 or Ephrin B1 on iPSC-aCM maturation in PC

**Fig. 6.**
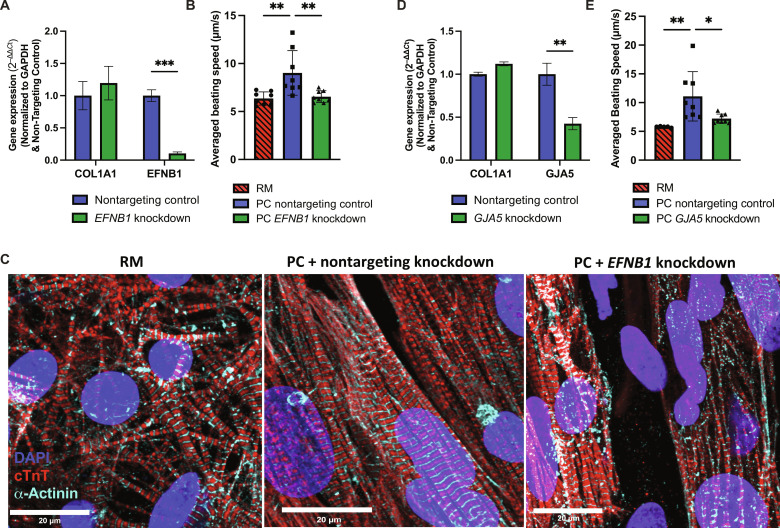
Knockdown of Cx40 or ephrin B1 (EFNB1) expression in primary adult ACFs before coculture with iPSC-aCMs. (**A**) Verification of siRNA-mediated *EFNB1* knockdown via gene expression analysis in ACFs 7 days following transfection (*n* = 6). Collagen 1a1 (*COL1A1*) was used an untargeted control ACF gene. Knocking down *EFNB1* in ACFs before coculture (**B**) lowers speed of iPSC-aCM contraction movement (*n* = 8) and (**C**) disrupts sarcomere organization in iPSC-aCMs as visualized via immunostaining of cTnT and α-actinin. (**D**) Verification of siRNA-mediated Cx40 (*GJA5*) knockdown via gene expression analysis in ACFs 7 days following transfection (*n* = 6). (**E**) Knocking down *GJA5* in ACFs before coculture lowers speed of iPSC-aCM contraction movement (*n* = 8). Scale bar, 20 µm.

Cx40 (*GJA5*) and Ephrin B1 (*EFNB1*) were found to be differentially up-regulated in ACFs compared to VCFs (fig. S1J), and Cx40 protein was localized to cell junctions between iPSC-aCMs and ACFs in PC (fig. S7). To determine the effects of these genes in iPSC-aCM maturation in PC, we performed siRNA knockdowns in ACF monoculture before coculture with iPSC-aCMs in PC and subsequently assessed functional output. Knockdown of *EFNB1* was verified through reverse transcription and quantitative polymerase chain reaction (RT-qPCR) and was maintained after 7 days of culture (Fig. 6A). In PC containing ACFs pretreated with *EFNB1* siRNA, we observed lower iPSC-aCM contraction movement to 73 ± 7% (Fig. 6B, donor 1, statistically significant) and 64 ± 4% (donor 2, not statistically significant) of the PC control containing ACFs pretreated with nontargeting siRNA. Furthermore, *EFNB1* knockdown caused disorganization of iPSC-aCM sarcomere structures (Fig. 6C) across both iPSC-aCM donors. When *EFNB1* was knocked down directly in PC, similar trends as above were observed in iPSC-aCMs (fig. S13). *GJA5* knockdown in ACFs was verified through RT-qPCR and was maintained after 7 days of culture (Fig. 6D). In PC containing ACFs pretreated with *GJA5* siRNA, we observed lower iPSC-aCM contraction movement to 65 ± 7% (Fig. 6E, donor 2, statistically significant) and 80 ± 14% (donor 1, not statistically significant) of the PC control containing ACFs pretreated with nontargeting siRNA. Knockdown of *EFNB1* or *GJA5* in ACFs lowered contraction movement in both iPSC-aCM donors cultured in PC to similar levels as in RM. Overall, the trends across the two donors of iPSC-aCMs showed that ACF-derived Cx40 and Ephrin B1 have roles in enhancing iPSC-aCM contraction movement, likely through effective electrical coupling (*23*) and optimal sarcomere organization/alignment (*24*), respectively. However, the above results also suggest that other factors may affect the maturation of specific iPSC-aCM donors in PC.

### PC utility for drug screening

**Fig. 7.**
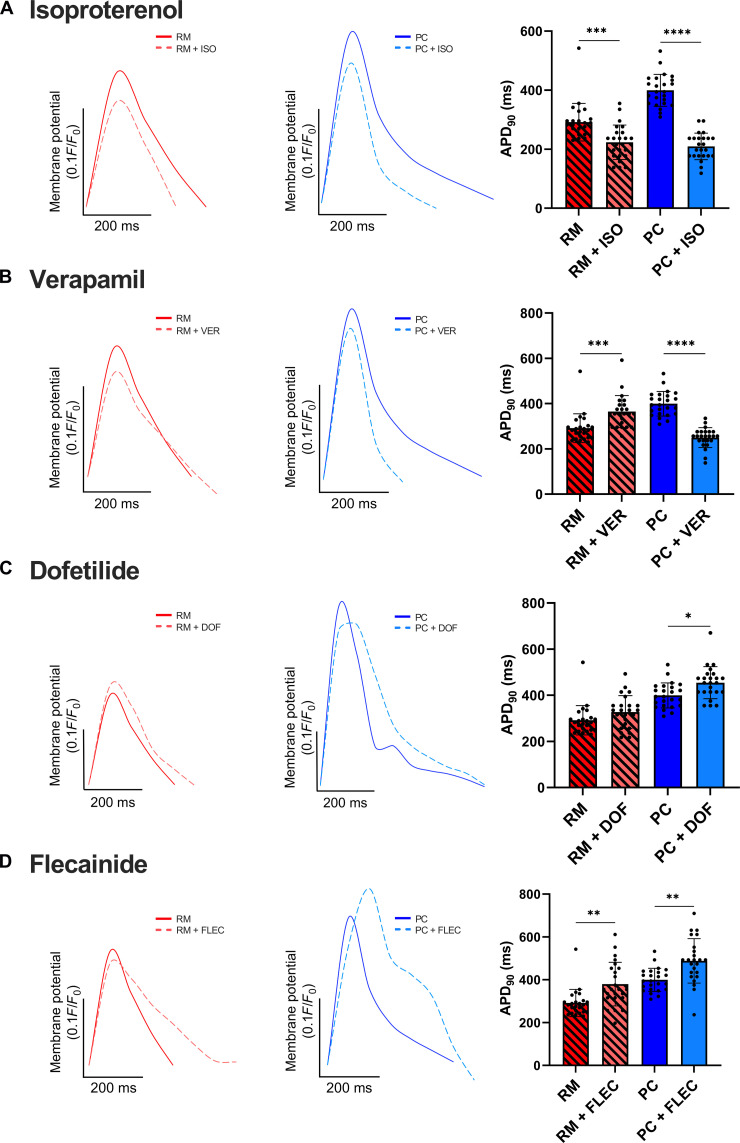
Prototypical drug response of iPSC-aCMs. (**A**) Isoproterenol (ISO)–treated RM and PC showed a statistically significant decrease in APD_90_, relative to vehicle [dimethyl sulfoxide (DMSO)]–treated control cultures; the relative decrease in APD_90_ was higher in PC compared to RM. (**B**) PC showed a significant decrease in APD_90_ when treated with verapamil (VER), while an increase was observed in RM. (**C**) PC showed a significant increase in APD_90_ in response to dofetilide (DOF), while no significant difference was observed in RM. (**D**) Flecainide (FLEC)–treated RM and PC both showed a significant increase in APD_90_. All panels have *n* = 24 cells. *P < 0.05, **P < 0.01, ***P < 0.001, and ****P < 0.0001.

Isoproterenol is a β-adrenergic agonist used to treat bradycardia by increasing beating frequency (*33*). While iPSC-aCMs in RM and PC showed significantly shorter APD_90_ with isoproterenol treatment, a larger difference was observed in PC at the same drug concentration (Fig. 7A). Verapamil, which blocks both I_CaL_ and the delayed rectifier potassium channel I_Kr_, shortens the QT interval in patients (*34*). While a significantly shorter APD_90_ was observed in PC following verapamil treatment, an increase was observed in RM (Fig. 7B). Dofetilide, a class III AAD that targets I_Kr_ and prolongs the effective refractory period (*35*), caused a statistically significant increase in APD_90_ only in PC and not RM (Fig. 7C). Last, flecainide, a class I_c_ sodium channel blocker used as an AAD to prolong depolarization (*36*) and known to inhibit I_Kr_ channels, resulting in APD prolongation (*37*), caused a significant increase in APD_90_ across both RM and PC (Fig. 7D). Similar trends were also observed in another iPSC-aCM donor (fig. S14).

**Fig. 8.**
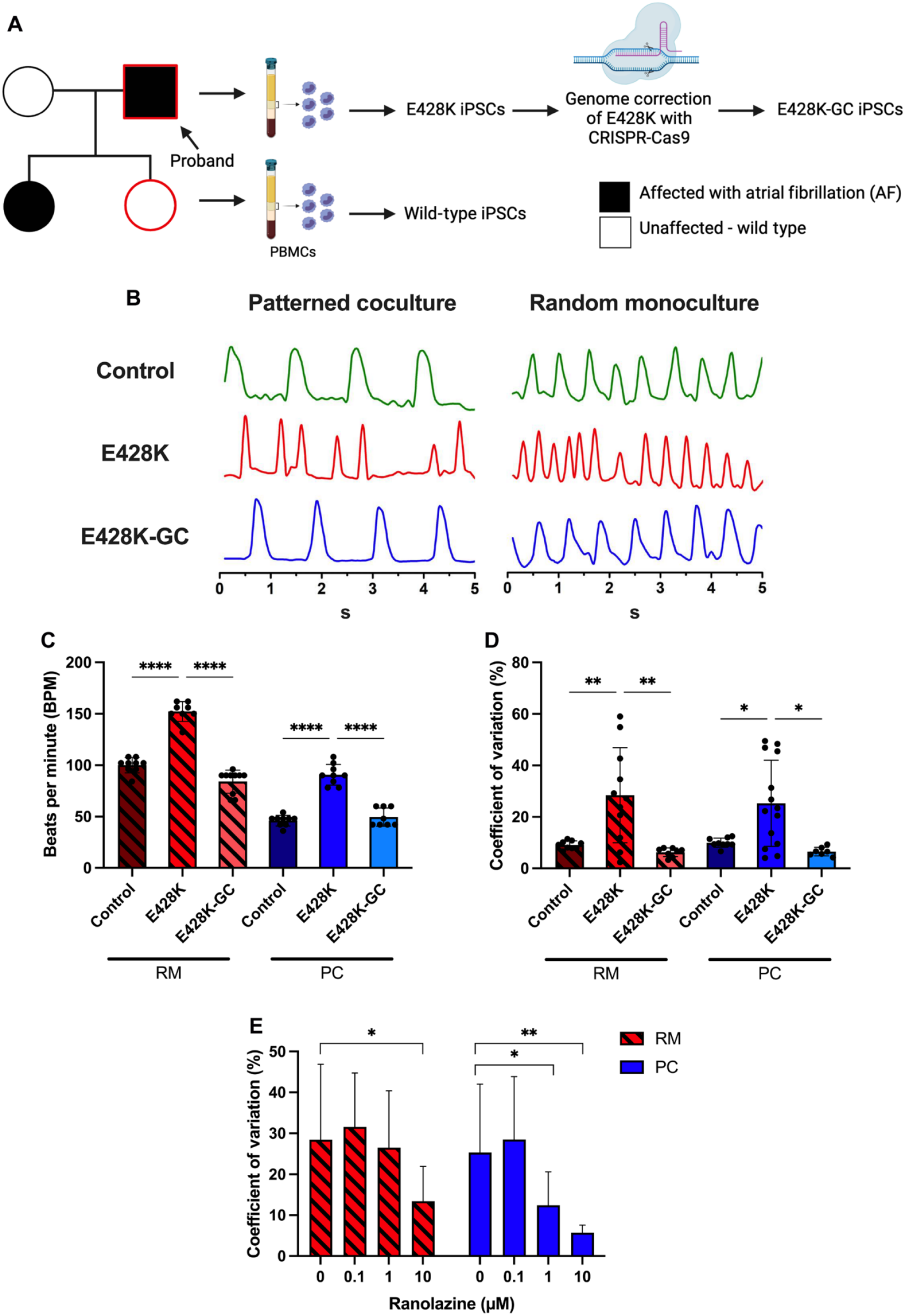
Modeling AF in iPSC-aCMs with E428K mutation in *SCN5A*. (**A**) Left to right: Schematics of family tree with E428K mutation and an unaffected genetic control (wild type), isolation of peripheral blood mononuclear cells (PBMCs), generation of iPSCs from PBMCs, and CRISPR correction to generate a genome-corrected (GC) control. Diagram constructed in BioRender. (**B**) OVM traces comparing PC to RM for the iPSC lines showed a uniform beating pattern in the control line and an irregular beating pattern in the E428K line, which was corrected with the E248K-GC line. (**C**) An increase in beats per minute was observed in the E428K line versus control and GC lines; however, values were higher in RM versus PC, which was closer to the adult heart’s beating rates (*n* = 8 cells). (**D**) An increase in coefficient of variation (mean peak-to-peak interval divided by the SD) was observed in the E428K line compared to the control and GC lines in both RM and PC (*n* = 8 cells for control and E428K-GC lines; *n* = 11 cells for RM E428K line; *n* = 14 cells for PC E428K line). (**E**) Coefficient of variation was significantly decreased in E428K-PC treated with both 1 and 10 μM ranolazine (Rano), whereas a significant decrease was observed only at 10 μM Rano treatment in E428K-RM, suggesting that PC are more sensitive to Rano treatment (*n* = 11 cells). *P < 0.05, **P < 0.01, and ****P < 0.0001.

To assess the ability to model AF using iPSC-aCMs in PC, peripheral blood mononuclear cells (PBMCs) were isolated from a patient with drug refractory AF who was heterozygous for a missense mutation (E428K) in *SCN5A*, which leads to gain of function as characterized by a significantly larger late sodium current (I_NaL_) than wild-type (WT) controls (*38*). PBMCs were also isolated from an unaffected family member who does not carry the genetic variant (WT control). The PBMCs were reprogrammed into iPSCs; a genome corrected (GC) iPSC line was also generated using CRISPR gene editing (Fig. 8A). The iPSC-aCMs generated from both the WT (Fig. 2B) and E428K (fig. S15, A and B) lines displayed a higher level of structural maturation in PC compared to RM. Furthermore, E428K-iPSC-aCMs exhibited a faster beating rate in RM and PC compared to WT- and E428K-GC-iPSC-aCMs; however, PC maintained an adult-like (lower) beating rate compared to RM (Fig. 8, B and C). Furthermore, the coefficient of variation based on peak-to-peak interval variability was significantly increased in E428K-iPSC-aCMs compared to WT- and E428K-GC-iPSC-aCMs in both PC and RM (Fig. 8D). However, we observed a slight (10 to 20%) decrease in APD_90_ in E428K-iPSC-aCMs in RM and PC compared to the WT and E428K-GC iPSC-aCMs; this result is in contrast to our previously published result (*38*) showing increased APD_90_ in E428K-iPSC-aCMs in RM, which could be due to differences in techniques used to quantify APD_90_ (patch clamping and multi-electrode array with field pacing used previously versus OVM used here). Last, when treated with different concentrations of ranolazine, a I_NaL_ channel blocker, PC exhibited a significant reduction in the coefficient of variation at both 1 and 10 μM, whereas RM exhibited a significant reduction in the coefficient of variation at only 10 μM (Fig. 8E); the *C*_max_ for ranolazine varies from ~1 to 14 μM in humans (*39*). APD_90_ was not affected at any of these ranolazine concentrations in both RM and PC (fig. S15, C and D). Overall, the results above show that PC are more sensitive than RM for disease modeling and drug screening.

## DISCUSSION

Owing to the paucity of primary human CMs, iPSC-CMs are being increasingly used for drug screening and to investigate genotype-phenotype relationships in cardiac diseases (*40*, *41*). However, iPSC-CMs in RM do not display adequate adult-like maturity (*40*, *42*) to always enable the sensitive detection of clinically predictive outcomes. Bioengineering technologies such as cell micropatterning, microfluidics, and three-dimensional (3D) bioprinting can improve iPSC-CM maturation, but many such technologies are not scalable for high-throughput screening applications and/or have not been adapted to iPSC-aCMs (*43*–*45*). Here, we mitigate such limitations by showing that a combination of iPSC-aCM micropatterning and coculture with primary adult ACFs significantly improves the structural, EP, contractile, and metabolic maturity of iPSC-aCMs, making them better suited for the detection of drug efficacy and gene editing outcomes than conventional monocultures.

We found that while both ACFs or VCFs led to less iPSC-aCM circularity and improved sarcomere organization compared to RM, sarcomere length in iPSC-aCM was significantly improved only with ACFs, as well as the expression of *SCN5A* and *KCNJ3*. Furthermore, while both ACFs and VCFs expressed similar levels of *Cx43* and *Cx45*, *Cx40* was significantly higher in ACFs, likely due to their atrial origin since Cx40 is expressed in the atria (*12*). Immunostaining analysis showed that Cx40 protein was indeed expressed in iPSC-aCM/ACF cocultures with staining patterns detected at heterotypic contacts between the two cell types. The expression of *EFNB1*, a regulator of CM shape/sarcomere organization (*24*), was also higher in ACFs than VCFs. Knockdown of ACF-derived *Cx40 (GJA5)* and *EFNB1* individually via siRNA treatment caused lower iPSC-aCM contraction movement in PC, while knockdown of ACF-derived *EFNB1* also caused iPSC-aCM sarcomeres to become disorganized compared to the nontargeting siRNA control. However, the extent of inhibition of iPSC-aCM contraction movement due to the knockdowns varied across iPSC-aCM donors, suggesting that other factors may also affect the maturation of specific iPSC-aCM donors in PC.

Micropatterning has proven to be useful for aligning iPSC-CMs and thereby their sarcomeres (*17*, *46*, *47*). However, it has been challenging to pattern proteins uniformly across the entire well surface area of multiwell plates that are routinely used for compound screening across several industries, such as pharmaceutical, chemical, and agricultural. To mitigate this limitation, we developed a soft lithographic process tailored for patterning a variety of ECM proteins within multiwell plates. Surface modification strategies were chosen to enable the patterning of ECM proteins to promote iPSC-aCM attachment to certain areas of the plate, while BSA patterning was used to deter iPSC-aCM attachment while still allowing for CF attachment. FN was chosen for iPSC-aCM attachment based on preliminary studies of iPSC-aCM attachment/retention on TCPS coated with collagen I, laminin, and vitronectin (VTN). FN line widths of 55 μm with 80-μm spacing were chosen to enable robust lateral contacts between two and three iPSC-aCMs (*48*) while allowing for heterotypic signaling and gap junctional communication from neighboring fibroblasts. The resulting PC containing both iPSC-aCMs and ACFs displayed significant improvements in multiple iPSC-aCM maturation metrics that were comparable to published ones in adult CMs and significantly better than fetal CMs and RM (Table 1) (*17*).

**Table 1.** Comparison of fetal and adult primary CM maturation parameters with iPSC-aCMs cultured in RM and PC.

	Fetal (immature) primary CMs (*17*)	Adult primary CMs (*17*)	iPSC-aCMs in RM	iPSC-aCMs in PC
**Cell shape**	Round and circular shape	Rod shape with AR ~1:7 and unidirectionally aligned	Circular shape with AR ~1:2 without spatial alignment. Flattened out monolayers.	Elongated shape with AR ~1:8 and unidirectionally aligned. 3D cell organiza tion develops over time in culture.
**Nuclei**	Circular shape, single nucleus	Elliptical shape and multi- nucleated [25% to 57% in adult myocardium (*74*, *75*)]	Circular and less multinucle ated (~10–20%)	Rod shape and more multi nucleated (~25–50%)
**Sarcomere structure and length**	Sparse and random distribution, ~1.6 μm	High density and anisotropic alignment, ~2.2 μm	Sparse and random distribution, ~1.7 μm	Dense and unidirectional alignment, ~2.1 μm
**Electrophysiology and calcium handling**	Following maturation, spontaneous beating stops and APD is prolonged (~400–450 ms) in right aCMs (*26*)		Short APD (~225–300 ms).	Prolonged APD (~350–500 ms)
	Calcium cycling is improved in adult CMs compared to fetal CMs		Low Ca^2+^ current density (−19.67 pA/pF)	Higher Ca^2+^ current density (−38.24 pA/pF)
	Gap junctions and adhesive junctions are polarized in adult CMs compared to fetal CMs		Randomly distributed gap junction (Cx40) staining patterns	Localization of Cx40 at cell boundaries
**Contraction**	Synchronized and unidirectional contraction with increased contractile force in adult CMs compared to fetal CMs		Beating at individual cell level with low contractile force (0.44 ± 0.25 μN)	Beating at integrated cellular patch level with higher contractile force (1.59 ± 0.64 μN)
**Metabolism**	Glycolysis-based respiration	Oxidative phosphorylation- based respiration	Lower maximum respiration	Higher maximum respiration
	Small and discrete mitochondria with random distribution	Large mitochondria with intermyofibrillar localization	Random distribution of mitochondria	Elongated mitochondria aligned around sarcomeres
**Ease of use for drug screening and disease modeling**	Rapidly lose functionality when cultured ex vivo		Amenable to multiwell plates allowing for ease of use with multichannel pipettes and automated fluid handlers for high- throughput screening	
			Amenable to high-content imaging analysis	

A distinctive maturation change observed in iPSC-aCMs in PC compared to RM and RC was the iPSC-aCM sarcomere structure, exhibiting an increased density, organization, and length, which are key regulators of other CM processes (*16*). We also observed an improved spatial rearrangement of mitochondria close to sarcomere structures in PC compared to RM via both fluorescent staining and TEM, which is consistent with literature showing that sarcomere maturation regulates mitochondrial size and developmental level through the serum response factor pathway (*16*). It was also previously reported that the sarcoplasmic reticulum (SR) spatially arranges closer to sarcomeres and mitochondria during the CM maturation process, thereby allowing for the efficient transport of ATP from adenosine triphosphatases in the sarcomere and SR (*16*, *17*). Here, the improved Ca^2+^ current density, increased CaT amplitude, decreased CaT decay tau, and prolonged APD_90_ observed in PC compared to RM may be due to enhanced ATP transport between SR and the sarcomere in PC, although further studies are needed to prove these links. Last, we also observed significantly increased multinucleation rates in iPSC-aCMs in PC (25 to 50%) compared to RM (10 to 20%), which may reflect the induction of cell cycle withdrawal (*25*, *49*, *50*). iPSC-aCMs in PC up-regulated the gene expression of cyclin-dependent kinase inhibitors, *CDKN2A* and *CDKN2B*, important for cell cycle withdrawal (*49*). Previous studies have shown that long-term culture (>100 days) was required to achieve iPSC-aCM bi/multinucleation (*25*, *50*), whereas iPSC-aCMs in PC achieved these outcomes in ~45 days, suggesting an accelerated maturation timeline useful for more timely execution of compound screening and disease modeling.

Another unique structural feature of PC was the reorganization of iPSC-aCMs into a 3D tissue-like structure. While single layers of iPSC-aCMs initially attached to the FN surface patterns, upon prolonged coculture with ACFs, the iPSC-aCMs in PC formed elongated tissue patches oriented along the micropatterns. These iPSC-aCM patches showed volumetric changes during contraction and smooth surfaces such as previous 3D iPSC-CM microstructures embedded within ECM hydrogels (*27*). Gaussian centroids in tissue thickness distribution of RM and PC were 1.675 to 3.659 μm and 6.638 to 6.981 μm, respectively. Because iPSC-aCMs maintained a single layer thickness throughout the culture duration in RM, the thickness distribution suggests that iPSC-aCMs form >2 layers over time in PC. In addition, RNA-seq analysis of purified iPSC-aCMs in PC versus RM showed up-regulation of several ECM-related genes suggesting that the three-dimensionality in PC could be the result of increased ECM deposition from both iPSC-aCMs and ACFs. In comparison to RM, iPSC-aCMs in PC also showed an increased expression of growth differentiation factor 15 (*GDF15*), a protein that was previously found to be highly expressed in cardiac tissues subjected to increased biomechanical stress (*51*). The greater expression of *GDF15* in iPSC-aCMs within PC, even in the absence of external mechanical stretch that requires specialized equipment, could be due to the greater contractile force observed via TFM in the multilayered PC compared to RM. Thus, PC provides an ideal balance between technological simplicity/throughput for drug screening and the 3D morphology/contractility of iPSC-aCMs.

Another notable maturation hallmark of iPSC-aCMs in PC was the up-regulation of the gene expression of integrin subunits, *ITGA5* and *ITGB1*, as well as cytoskeleton/structure proteins (*52*), which may underlie the improved contraction directionality and contraction force of iPSC-aCMs within PC. Previous studies have shown that iPSC-CM culture on a soft Matrigel-coated PDMS substrate induced β1-integrin expression and integrin receptors genes, including *ITGA5* and *ITGB1* (*53*). The up-regulation of integrin and cytoskeleton genes in iPSC-aCMs in PC in a similar way as the above study on a soft substrate suggests that PC generated the appropriate 3D cell-ECM interactions to mitigate any detrimental effects of the hard plastic used to generate the initial cell patterns in industry-standard multiwell plates.

The iPSC-aCMs in PC showed increased OCR compared to RM, including spare respiratory capacity, ATP production, maximum respiration, and basal respiration, which is a hallmark of maturation as adult CMs rely heavily on oxidative phosphorylation rather than glycolysis (*30*). Protein expression of mitochondrial complexes I, II, III, and V (involved in oxidative phosphorylation) were fairly similar across both PC and RM, likely due to the inclusion of fatty acids (FAs) and TID [T3, insulin-like growth factor-1 (IGF-1), dexamethasone] soluble factors in the culture medium, which we have previously shown to improve the metabolic maturation of iPSC-aCMs in RM as well (*54*). Overall, our results suggest that the greater metabolic maturation of iPSC-aCMs in PC compared to RM may be due to enhanced ATP production and utilization by closely aligned elongated mitochondria and organized sarcomeres.

Both RM and PC responded in clinically meaningful ways to isoproterenol and flecainide. However, for the calcium channel blocker verapamil, only PC displayed a statistically significant decrease in APD_90_, which may be due to the significant increase in the Ca^2+^ current observed in PC compared to RM. Dofetilide, a class III AAD that targets I_kr_ and prolongs the effective refractory period (*35*), caused an increase in APD_90_ in both RM and PC, but the increase in RM did not reach statistical significance for one of the two iPSC-aCM donors. Furthermore, while a statistically significant decrease in APD_90_ was observed in both RM and PC following isoproterenol treatment, PC displayed a larger decrease (1.7-fold versus 1.1-fold). While sodium channel (*SCN5A*) mutant E428K-iPSC-aCMs exhibited a faster beating rate and greater coefficients of variation (based on peak-to-peak interval variability, thereby indicating irregular AF-like beating patterns) in both RM and PC compared to WT-and E428K-GC-iPSC-aCMs, only PC maintained an adult-like (lower) beating rate. Last, ranolazine, an I_NaL_ channel blocker, reduced coefficients of variation at both 1 and 10 μM in E428K-iPSC-aCM–containing PC compared to only 10 μM in RM; ranolazine *C*_max_ in humans is known to vary from ~1 to 14 μM (*39*). The disease modeling and drug efficacy results above in totality demonstrate greater sensitivity of PC compared to RM for detecting clinically relevant outcomes in both WT and mutant iPSC lines.

While PC in multiwell plates significantly enhanced iPSC-aCM maturation compared to RM, sodium current density was similar in both. In vivo, CMs are subjected to other microenvironmental cues, such as electrical pacing and mechanical stretch due to flowing blood. In the future, we anticipate that introduction of additional stimuli to the multiwell PC format can further mature iPSC-aCMs. For example, PC can be adapted to commercially available electrical stimulation equipment or generated on (i) elastomeric substrates (e.g., PDMS) compatible with mechanical stretching equipment, (ii) within microfluidic devices for perfusion with an endothelial layer, and (iii) multielectrode arrays to enable real-time electrical pacing and monitoring (*55*). However, static multiwell plate PCs, which are more accessible and cost-effective, will likely suffice for routine drug screening and modeling certain aspects of AF as we have shown here. Last, coculture of iPSC-aCMs with fetal CFs first, either primary (*56*) or iPSC-derived if genotype matching is needed (*57*), followed by coculture with adult CFs may lead to higher maturation than coculture with only adult CFs; to enable such a dynamic coculture strategy, reconfigurable cell culture platforms (*58*) can be adapted to PC while still maintaining the multiwell format for compound screening.

In conclusion, a combination of cell micropatterning and coculture with ACFs (versus VCFs) leads to significantly greater structural, EP, contractile, and metabolic maturity in iPSC-aCMs than in randomly distributed monocultures and cocultures. This approach makes PC suitable for compound screening and modeling the effects of genetic mutations in AF-like phenotypes in iPSC-aCMs using industry-standard multiwell plates amenable to high-throughput investigations.

## MATERIALS AND METHODS

### CF cell culture

Cryopreserved primary human adult CFs of both atrial (ACF) and ventricular (VCF) origin, two donors for each type of fibroblast, were obtained from Lonza (Walkersville, MD). CFs were grown and passaged with culture medium containing 10% (v/v) fetal bovine serum (FBS; Thermo Fisher Scientific, Waltham, MA) in Ham’s F-12 medium containing l-glutamine (Thermo Fisher Scientific), 1% (v/v) penicillin/streptomycin (P/S) (Corning, Corning, NY), basic fibroblast growth factor (4 ng/ml; STEMCELL Technologies, Vancouver, Canada), and human insulin (20 μg/ml; MilliporeSigma, Burlington, MA). CFs were grown to ~80% confluency and passaged no more than six times. Before seeding the CFs onto iPSC-aCMs, the cells were treated with mitomycin C (1 μg/ml; MilliporeSigma) as a strategy to prevent overgrowth of the fibroblasts (*59*) in coculture with iPSC-aCMs.

### Differentiation of iPSC-aCMs

iPSCs were seeded on human recombinant VTN (Thermo Fisher Scientific)–coated six-well plates and cultured in mTeSR medium (STEMCELL Technologies) with media changes daily. Once iPSCs reached 80 to 90% confluence, differentiation was initiated by performing a media change to medium A (day 1) of the PSC Cardiomyocyte Differentiation Kit (Thermo Fisher Scientific) in which the cells were incubated for 48 hours to cause mesodermal induction. Next, the medium was replaced with medium B of the aforementioned kit and incubated for 48 hours to induce CM differentiation. On day 5, the cells were treated with 1 μM all-trans retinoic acid (MilliporeSigma), which has been shown previously to promote an atrial phenotype (*60*), for 4 days with a culture medium change every 2 days. Last, further CM purification was achieved via glucose starvation with sodium dl-lactate (40 mM; MilliporeSigma) replacement for six additional days (*61*). After CM purification, iPSC-aCMs were cultured in cardiomyocyte maintenance media (CMM; Thermo Fisher Scientific) for 16 to 18 days and then used for further experiments.

### Protein micropatterning on TCPS

The TCPS 24-well plate surface (CellVis, Mountain View, CA) was chemically modified with APTES (MilliporeSigma) to increase the adhesiveness of the PDMS mask and FN. Briefly, the TCPS 24-well plate was treated with oxygen plasma [O_2_ gas flow (2.5 cm^3^/min) with plasma power at 100 W, PE-50 model, PlasmaEtch, Carson City, NV] for 2 min followed by treatment with 5% APTES in 95% ethanol (EtOH; MilliporeSigma) for 30 min at room temperature. In this process, the hydroxyl groups in plasma-treated TCPS surface were covalently conjugated with hydroxyl groups in hydrolyzed APTES (*62*). After rinsing with 100% EtOH, the plate was dried under a N_2_ stream and then thermally cured at 75°C for 1 hour. This thermal curing is known to stabilize the covalent coating of APTES on the TCPS surface (*63*, *64*). The PDMS mask was then adhered to each well of the APTES-coated 24-well plate, which was again treated with oxygen plasma (same conditions as above) for 2 min to ablate away the APTES not protected by the PDMS mask. Then, 0.05% (w/v) BSA (Thermo Fisher Scientific) in double deionized water (ddH_2_O) was loaded to each well and allowed to penetrate each microchannel made by the PDMS mask via capillary forces and applying gentle vacuum (721.3-mbar maximum vacuum, Cole-Parmer, Vernon Hills, IL) to the dry side of the channel. The plate was then incubated at 4°C overnight. After BSA blocking (*65*), the solution was removed and rinsed with ddH_2_O, while the PDMS mask was still attached. The PDMS mask was then removed from the TCPS surface and washed again with ddH_2_O. The plate was then sterilized via ultraviolet light for 1.5 hours. Last, human FN (30 μg/ml; Corning) in 1× Dulbecco’s modified phosphate-buffered saline (DPBS; Corning) was then added to each well, incubated at 4°C overnight, and washed with DPBS three times before cell seeding.

### Micropatterning of iPSC-aCMs and CFs

Between days 16 and 18 after initiation of differentiation, iPSC-aCM cultures were incubated with DPBS without Ca^2+^ and Mg^2+^ for 20 min at 37°C. Next, TrypLE Express (Thermo Fisher Scientific) was added to the cells and incubated for 5 min at 37°C, followed by Liberase (25 μg/ml; MilliporeSigma) for 20 min. The cells were then transferred to a conical tube and centrifuged for 5 min at 500*g*. After removing the supernatant, the cells were resuspended in CMM supplemented with 10% FBS and 1% (v/v) P/S. The iPSC-aCMs were then seeded onto the FN/BSA-micropatterned 24-well plate at a cell density of 0.8 × 10^6^ cells/ml. After the initial seeding, plates were maintained in a 37°C incubator. For even distribution of iPSC-aCMs on the FN micropatterns, the plate was shaken every 20 min for 3 hours. After 48 hours, CFs were passaged and resuspended in media containing 2% FBS, P/S, and additional factors that have been shown to lead to CM maturation, specifically 100 nM T3 (MilliporeSigma), IGF-1 (100 ng/ml; PeproTech, East Windsor, NJ), 1 μM dexamethasone (BioGems, Westlake Village, CA) (*54*, *66*), herein referred collectively as “TID,” and FAs (100 μM oleic acid and 50 μM palmitic acid, MilliporeSigma) complexed with BSA, herein referred to as “CMM-TID/FA” media. Before CF seeding, the micropatterned iPSC-aCMs were rinsed with CMM. Then, the CFs resuspended to a density of 0.33 × 10^6^ cells/ml were seeded onto the micropatterned iPSC-aCMs and incubated at 37°C. To minimize the aggregation and uneven distribution of CFs, the plate was shaken every 20 min for the first 2 hours. For the remaining cocultivation period, the medium was changed with serum-free CMM-TID/FA media every 2 days.

### Magnetic activated cell sorting

To sort the iPSC-aCMs from the CFs for CM-specific end-point analysis, the cells were disassociated as described above. Once the cells were in suspension, the PSC-Derived Cardiomyocyte Isolation Kit (Miltenyi Biotec, Gaithersburg, MD) was used to isolate the CMs via negative selection per the manufacturer’s protocol with LS columns and a MidiMACS Separator magnet (Miltenyi Biotec). Only labeling of the non-CM population was performed to reduce handling of the iPSC-aCMs. This has been shown previously to be sufficient for CM isolation (*67*) and, in our hands, yielded ~80 to 90% pure iPSC-aCMs and <6% fibroblasts based on cTnT and collagen 1a1 (Col1a1) immunostaining analysis, respectively (fig. S16). CM cell sorting was performed before RT-qPCR, RNA-seq, patch clamping, and Seahorse analysis.

### Gene expression analysis

Cell samples for RNA-seq and RT-qPCR analysis were both lysed with TRIzol (Invitrogen, Waltham, MA), and the RNA fraction was further isolated by introducing chloroform, centrifuging at a 12,000*g*, and removing the top fraction. The samples were further purified with the RNeasy kit (QIAGEN Sciences, Germantown, MD). Genomic DNA was then removed via deoxyribonuclease I treatment (MilliporeSigma) per the manufacturer’s protocol. After purification, cDNA was then synthesized for qPCR analysis with the AzuraFlex cDNA Synthesis Kit (Azura Genomics, Raynham, MA). Next, cDNA was combined with PowerUp SYBR Green Master Mix (Applied Biosystems, Waltham, MA) and a gene-specific primer (Integrated DNA Technologies, Coralville, IA). Reported values were calculated via the ∆∆*C*_T_ method compared to a control (specified in each figure) with *RPL37A* or glyceraldehyde-3-phosphate dehydrogenase as the housekeeping gene.

RNA-seq was performed at the University of Chicago Genomics Facility. Library preparation was performed via the TruSeq Stranded mRNA Kit (Illumina, San Diego, CA) following the manufacturer’s protocol. Libraries were sequenced on the Illumina NovaSeq 6000. Next, data alignment to the human genome was performed using the BioJupies platform (*68*) maintaining the default parameter settings. The R package edgeR (*69*) was further used for data normalization. PCA was performed after normalization, and differentially expressed genes were identified with false discovery rate < 0.05 and an absolute log_2_ fold change of greater than 1. The adjusted *P* value was further assessed with the *P*-adjust function in edgeR. GO pathway analysis was performed in Cytoscape with the ClueGO (*70*) package for analysis and visualization. All RNA-seq data has been deposited in the Gene Expression Omnibus (www.ncbi.nlm.nih.gov/geo/) accession number GSE239322.

### OCR analysis

OCR was assessed using the Seahorse XFe96 Analyzer (Agilent Technologies, Santa Clara, CA). Four days before analysis, iPSC-aCMs were sorted via magnetic activated cell sorting (MACS) and replated onto FN-coated Seahorse XFe96 cell culture microplates at a density of 40,000 cells per well with media changes every other day. The assay was performed as described previously (*54*). Briefly, culture medium on cells was changed to 100 μl per well of bicarbonate-free RPMI 1640 (Agilent Technologies) and incubated in a non-CO_2_ incubator for 1 hour at 37°C. Drugs were then loaded into the cartridge with a final effective concentration as follows: 2.5 μM oligomycin, 5 μM carbonyl cyanide (trifluoromethoxy) phenylhydrazone, and 2.5 μM antimycin A + rotenone.

### Optical voltage mapping

Cells were washed five times with indicator free Tyrode’s solution (Boston BioProducts, Milford, MA). VF2.1C1 voltage sensitive dye (MilliporeSigma) was first reconstituted to a concentration of 2 mM in dimethyl sulfoxide (DMSO), followed by dilution to 1 mM with 10% Pluronic F-127 (MilliporeSigma). Last, the dye/pluronic mixture was diluted to 100 nM in Tyrode’s solution and added to the cells. The cells were incubated with the dye for 45 min at 37°C, followed by five washes with indicator-free Tyrode’s solution. After allowing the cells to recover, the dye was excited at 514 nm, and an image time series was acquired to assess fluorescent changes over time. Images were captured on a spinning disk confocal microscope (Zeiss, Pleasanton, CA) at either 10 or 15 fps for a total of 200 frames. The image series were then analyzed as described previously to calculate APD_90_ and peak-to-peak duration (*54*). Briefly, areas on individual iPSC-aCMs were identified and selected with the region of interest manager in ImageJ. Then, the integrated intensity was extracted over the time series and imported into Microsoft Excel. The membrane potential was then calculated by first determining the approximate average of the lowest points (*F*_0_) and dividing all the integrated intensity values by that value (*F*/*F*_0_). The data points were then graphed and analyzed in ImageJ software to determine APD_90_ and peak-to-peak duration. This analysis was performed for three cells per image series and three image series per condition.

### Automated patch clamp recording

After sorting via MACS as described above, iPSC-aCMs were replated onto FN-coated six-well plates and cultured for an additional 6 days. The cells were then disassociated as follows. Briefly, medium was aspirated from wells. PBS (1 ml) without Ca^2+^/Mg^2+^ was added to each well and swirled gently two to three times. The solution was replaced with 2 ml of fresh PBS without Ca^2+^/Mg^2+^ per well and incubated for 30 min at 37°C in 5% CO_2_. After this period, solution was removed from the wells and 1 ml of PBS without Ca^2+^/Mg^2+^ containing Liberase TH Research Grade (25 μg/ml; MilliporeSigma) was added to each well. Cells were then incubated for 25 min at 37°C in 5% CO_2_. After incubation, cells in each well were separated by gentle trituration using a 5-ml pipette and then the cell mixture was transferred to a 15-ml conical tube. PBS (1 ml) without Ca^2+^/Mg^2+^ was added to each well to recover remaining cells, added to the cell mixture in a conical tube, and gently mixed by aspirating up and down with a 5-ml pipette. A cell aliquot (500 μl) was used to determine cell number and viability by automated cell counting (ViCell, Beckman Coulter, Brea, CA). Cells were diluted to 500,000 cells/ml with PBS without Ca^2+^/Mg^2+^ and allowed to recover 20 to 30 min at 15°C while shaking on a rotating platform at 250 rpm before recordings commenced. Automated patch clamp recordings were performed to assess sodium and calcium currents using the SyncroPatch 384 platform (Nanion Technologies, Germany) with single-hole, 384-well thin-glass recording chips. An extended protocol can be found in supplemental material and methods. Data was then further analyzed and plotted through a combination of DataController384 V1.8.0.24 (Nanion Technologies), Excel, SigmaPlot V14 (Systat Software, Chicago, IL), and GraphPad Prism (San Diego, CA). In addition, custom semi-automated data handling programs were used for analysis of current density and voltage dependence of activation and inactivation. Whole-cell currents were further normalized based on membrane capacitance, and results were expressed as means ± SEM.

### Drug testing

Cultures were treated with the respective drugs for 30 min at day 45 after initiating CM differentiation, followed by OVM analysis. All drugs were reconstituted in DMSO and diluted to a final concentration of 0.1% DMSO (v/v) in indicator-free Tyrode’s solution. Cultures were treated with isoproterenol (1 μM), verapamil (1 μM), dofetilide (3 nM), or flecainide (10 μM). All drugs were purchased from Cayman Chemicals (Ann Arbor, MI). Drug concentrations were chosen on the basis of the previous in vitro literature (*27*, *71*, *72*). Untreated vehicle controls with 0.1% DMSO (v/v) were maintained for comparison.

### Disease modeling of AF

A three-generation family that maintained familial AF was originally enrolled in a clinical biorepository study (*73*). Whole-blood samples were obtained from the proband (SCN5A 1041: 62-year-old Caucasian male) and an unaffected family member (SCN5A 1043: 48-year-old Caucasian female), and iPSC lines were generated by Stanford Cardiovascular Institute as described previously (*38*). The specific underlying genetic mutation leading to AF was previously identified as a point mutation specifically in the E428K region of the SCN5A gene, and a GC iPSC line was generated using CRISPR-Cas9 as described previously (*38*). To assess the effect of ranolazine on the E428K iPSC-aCMs, ranolazine (Cayman Chemicals) was diluted in indicator-free Tyrode’s solution at concentrations ranging from 0.1 to 10 μM and incubated with the cells for 30 min before OVM assessment as described above. Coefficient of variation was assessed by determining the peak-to-peak values and calculating the mean and SD for each cell. Then, the SD was divided by the mean and multiplied by 100 to calculate the coefficient of variation.

### Statistical analysis

All findings were validated in two or more independent experiments with three to four replicate wells per condition from two iPSC donors, which included SCN5A 1043 (48-year-old Caucasian female) and Pt46 (74-year-old African American Male) (*54*). Data shown in figures represent one experiment with a single iPSC donor, unless otherwise indicated. Unless otherwise specified, data are represented as mean and SD. For datasets with two groups, a two-tailed unpaired Student’s *t* test was performed to determine statistical significance. For datasets with more than two experimental groups, a one-way analysis of variance (ANOVA) with a post hoc Tukey’s correction was applied. Significance was depicted on figures as *P < 0.05, **P < 0.01, ***P < 0.001, and ****P < 0.0001, with *P* < 0.05 as the level of significance.

## Supplementary Materials

### This PDF file includes:

Supplementary Materials and Methods Figs. S1 to S16 Legends for movies S1 to S8 Code for calcium transient parameter analysis References
