# Supplementary material for: Engineered cocultures of iPSC-derived atrial cardiomyocytes and atrial fibroblasts for modeling atrial fibrillation
Source: Sci Adv. 2024 Jan 19;10(3):eadg1222. doi: 10.1126/sciadv.adg1222 (PMC10798559; doi:10.1126/sciadv.adg1222)
Supplement: Supplementary file 1 — Supplementary Materials and Methods Figs. S1 to S16 Legends for movies S1 to S8 Code for calcium transient parameter analysis References [file sciadv.adg1222_sm.pdf]

Science Advances
AAAS

Supplementary Materials for

Engineered cocultures of iPSC-derived atrial cardiomyocytes and atrial fibroblasts for modeling atrial fibrillation

Grace E. Brown *et al.*

Corresponding author: Salman R. Khetani, skhetani@uic.edu; Dawood Darbar, darbar@uic.edu

Sci. Adv. **10**, eadg1222 (2024)
DOI: 10.1126/sciadv.adg1222

The PDF file includes:

Supplementary Materials and Methods
Figs. S1 to S16
Legends for movies S1 to S8
Code for calcium transient parameter analysis
References

Other Supplementary Material for this manuscript includes the following:

Movies S1 to S8

SUPPLEMENTARY MATERIALS AND METHODS

Fabrication of polydimethyl siloxane (PDMS) stamps

To prepare PDMS stamps, a SU-8 photoresist master mold was fabricated by using a conventional SU-8 photolithography approach. Briefly, SU-8 2100 (Kayaku Advanced Materials, Westborough, MA) was spin coated and pre-baked on a silicon wafer (University Wafers, Boston, MA) at a 100 µm thickness. Next, the SU8 layer was exposed to UV at 250 mJ/cm^2 power in the presence of a film photomask exhibiting the desired linear pattern shape (55 µm in width for opaque region and 80 µm in width for transparent region). After developing and hard-baking, areas of linear SU8 micropatterns (80 µm in width) were visible. To cast the PDMS stamps, Sylgard-184 base and curing agent (Dow-Corning, Midland, MI) were mixed at a 10:1 ratio (w/w) and poured onto the SU-8 master mold. After degassing for 30 minutes in a vacuum chamber, the PDMS-poured master mold was thermally cured at 75°C in an oven. The cured PDMS replicate was peeled off from the master mold and cut into the desired dimensions. The prepared PDMS stamps were cleaned and sealed by 3M Scotch tape to protect from dust and debris until further use.

Fluorescent visualization of micropatterns

For the visualization of fibronectin (FN) and bovine serum albumin (BSA) micropatterns, 0.05% (w/v) BSA solution containing Rhodamine-polyethylene glycol-thiol (PEG-SH, 0.1 mg/mL, Nanocs, New York, NY) was prepared in double deionized water (ddH$_2$O) and used in the FN/BSA-micropatterning process described in the methods of the main manuscript. After the fibronectin/BSA-micropatterning, the well plate surface was blocked with a 0.3% BSA solution for 1 hour at room temperature (RT) and then incubated with an anti-human FN mouse IgG antibody (Thermo-Fisher, Waltham, MA) for 1 hour. Next, a secondary antibody (Alexa Fluor® 488 donkey anti-mouse IgG, Thermo-Fisher) was applied for 1 hour at RT, followed by washing with Dulbecco's phosphate buffered saline (DPBS). The resulting fluorescent signals on the micropatterns were visualized by an Olympus IX83 epi-fluorescent microscope using the Green Fluorescent Protein (GFP) and Red Fluorescent Protein (RPF) filters. After 2 days following induced pluripotent stem cell-derived atrial cardiomyocyte (iPSC-aCM) attachment onto FN micropatterns, the cells were rinsed with Cardiomyocyte Maintenance Media (CMM, Thermo-Fisher), treated with Calcein AM (Thermo-Fisher, 2 µM) in CMM for 30 minutes at 37°C, rinsed again with CMM, and imaged via epifluorescence microscopy using the GFP filter. Next, primary adult atrial cardiac fibroblasts (ACFs) were dissociated from the flask as described in the methods of the main manuscript and stained with CellTracker™ (Thermo-Fisher) per the manufacturer's protocol. The labeled ACFs were seeded onto the patterned iPSC-CMs and incubated for 1 day at 37°C. Lastly, the fluorescently stained iPSC-CM and ACF patterns were visualized via epifluorescent microscopy using GFP and Cy5 filters. All the antibodies were diluted in a 0.3% BSA solution at 1:200 dilution.

Small interfering RNA (siRNA) Knockdown of Cx40 and Ephrin1B in ACFs

ACFs were treated with 10 pmol ON-TARGET plus siRNA against human *GJA5* (Horizon Discovery, Cambridge, United Kingdom) or 10 pmol non-targeting siRNA control pool (Horizon

Discovery) with lipofectamine RNAiMAX (Thermo-Fisher) per the manufacturer's instructions. Briefly, the siRNA and the lipofectamine RNAiMAX were diluted separately in Opti-MEM media (Thermo-Fisher), combined at a 1:1 ratio, and incubated for 5 minutes at RT. The siRNA-lipid complex was then added to the cells in a dropwise manner and incubated for 48 hours followed by a medium change. The treated (*GJA5* or non-targeting siRNA) ACFs were then allowed to recover for 1 day prior to seeding in patterned coculture (PC) with iPSC-aCMs. In separate experiments, ACF monocultures or 2-day old PCs were treated with 10 pmol ON-TARGET plus siRNA against human *EFNB1* (Horizon Discovery) or 10 pmol non-targeting siRNA control pool (Horizon Discovery) with lipofectamine RNAiMAX (Thermo-Fisher) as described above. The treated (*EFNB1* or non-targeting siRNA) ACFs were then allowed to recover for 1 day prior to seeding in PC with iPSC-aCMs. Knockdown of each gene was assessed in parallel ACF monocultures via gene expression analysis at 7 days post-transfection.

Immunofluorescent Staining of Micropatterned iPSC-aCMs

For the immunofluorescent staining of the sarcomere structure (α-actinin and cardiac troponin T: cTnT), micropatterned iPSC-aCMs were fixed with 4% paraformaldehyde (PFA, Millipore Sigma, Burlington, MA) for 25 minutes at RT. Then, the fixed cultures were permeabilized and blocked with blocking buffer containing 5% donkey serum and 0.3% Triton X-100 (Tx-100) for 1 hour at RT. Primary antibodies for cTnT (rabbit-IgG, Abcam, Waltham, MA, RRID:AB_956386) and α-actinin (mouse-IgG, Abcam, RRID:AB_307264) were diluted in dilution buffer A (0.1% BSA and 0.3% Tx-100) and incubated overnight at 4°C. In certain cases, fibroblast visualization was facilitated by staining them with an antibody against human collagen 1a1 (sheep-IgG, R&D Systems, RRID:AB_10891543). Next, the cultures were washed with DPBS and treated with secondary antibodies (Alexa Fluor® 568 donkey anti-rabbit IgG and Alexa Fluor® 647 donkey anti-mouse IgG, Thermo-Fisher) in dilution buffer A for 1 hour at RT. Next, 2.7 μM of 4′,6-diamidino-2-phenylindole (DAPI, Thermo-Fisher) was added to the cultures for 15 minutes at RT to stain the nuclei. Finally, the cultures were washed with DPBS three times and analyzed via laser scanning confocal microscopy at 63x magnification (LSM 710, Zeiss, Pleasanton, CA).

The immunofluorescent staining of Cx40, Cx43, and α-actinin was carried out using the aforementioned staining protocol but with some modifications. Specifically, after PFA fixation, the cultures were permeabilized with 0.1% Tx-100 solution for 10 minutes at RT and then rinsed with DPBS three times. Next, 5% donkey serum was added to the cultures for 1 hour at RT followed by treatment with primary antibodies against Cx43 (rabbit-IgG, Cell Signaling Technology, Danvers, MA, RRID:AB_2294590) or Cx40 (rabbit-IgG, Thermo-Fisher, RRID:AB_2533263) with the addition of the abovementioned α-actinin antibody diluted in a dilution buffer B (0.3% BSA). After the antibody incubation at 4°C overnight, the cultures were rinsed with DPBS three times followed by incubation with secondary antibodies (same antibody pairs as with sarcomere staining) in dilution buffer B for 1 hour at RT. Finally, after DAPI staining and rinsing with DPBS, the immunostained cultures were visually analyzed via laser scanning confocal microscopy at 63x magnification. All the solutions for fixation, permeabilization, and antibody dilution were prepared in DPBS.

For fluorescent staining of mitochondria and cell membrane, MitoView™ Green (Biotium, Fremont, CA) (76) and Wheat Gum Agglutinin (Alexa Fluor® 568-labeled WGA , Biotium) (77)

were employed, respectively. To prepare working solutions, MitoView was diluted in DPBS to a concentration of 5 μg/mL and WGA was diluted in 1x Hanks' Balanced Salt Solution (HBSS, Thermo-Fisher) to a concentration of 100 nM. Prior to staining, iPSC-aCM/ACF micropatterns were fixed with PFA according to the abovementioned protocol. Then, MitoView was added to the plate and incubated for 30 minutes at RT. Next, the plate was sequentially rinsed 2X with DPBS and then HBSS. WGA was then applied to the cultures and allowed to react for 1 hour at RT. After WGA incubation, the plate was sequentially rinsed with HBSS and DPBS. Finally, for the analysis of co-localization of mitochondria and the cardiac sarcomeres, immunofluorescent staining of α-actinin was further performed on the MitoView/WGA-labeled iPSC-aCMs using the abovementioned sarcomere staining protocol. The resulting labeled cells were visualized on a laser scanning confocal microscope with GFP, RFP and Cy5 channels. All the primary and secondary antibodies were diluted at 1:200 ratio except the Cx43 antibody, which was diluted at 1:100 ratio.

Directionality Analysis for Micropatterned iPSC-CMs

At day 7 of culture post-replating, phase contrast images of PC of iPSC-aCMs and ACFs or random iPSC-aCM monocultures (RM) were captured as TIFF image files at 10x magnification. At day 25 of culture post-replating, cultures of RM, PC with mitomycin-C-treated ACFs, and PC with non-treated ACFs were stained with cTnT and fluorescent images were captured at 20x magnification. Separate PC cultures were fixed at days 2, 9, 15, and 21 post-replating, stained for cTnT, and fluorescently imaged at 20x magnification. The directionality and orientation of iPSC-CMs and cardiac fibroblasts in the image files were analyzed by FIJI software using the Directionality analysis function with the Local Gradient Orientation option. The orientation angle range for the histogram was set from 0° to 180°. The acquired orientation data was plotted onto a histogram using Prism (GraphPad, San Diego, CA) and the directionality data was depicted as a bar graph. Three different images were taken and analyzed for each condition and the resulting data was averaged for the plotted data.

Image Analysis

From the acquired fluorescent confocal microscopic images of iPSC-aCMs, the structural maturation indicators for cardiomyocytes, including cell aspect ratio, sarcomere length, and nuclei shape, were analyzed with Zeiss's Zen Blue Software and NIH ImageJ software. For the cell aspect ratio analysis, two different channels including WGA and α-actinin were utilized for analysis. As WGA stained all cell boundaries, including ACFs, only α-actinin-expressing cells were selected for aspect ratio analysis for the iPSC-aCMs. On the defined iPSC-aCM regions, the vertical and horizontal lengths of the cell were measured in the Zen Blue software. Six cells were analyzed for each image, and three images were analyzed per condition. As a result, 18 data points were collected from each condition and were plotted. To measure the sarcomere length, α-actinin stained images were loaded into the Zen Blue software. Then, on 13 sarcomere regions per image, lines perpendicular to the direction of sarcomeres (α-actinin signal) were drawn. By using the profile function, the fluorescent intensity profiles of each pixel's locations on the line were measured. The data set was transferred to Origin 8.0 and analyzed with the peak finding function. From this analysis, peak-to-peak length was assessed. The analysis was repeated for four individual images per condition. The acquired 52 data points per condition were plotted using GraphPad Prism. Nuclei shape analysis was carried out using NIH ImageJ. For coculture images, only cells

expressing α-actinin were assessed. After threshold adjustment on the NIH ImageJ software, all nuclei located in α-actinin-expressing regions were selected via the multiple regions of interest (ROI) selection tool and then analyzed. The data collection was repeated with four different images per condition and the collected data was plotted in GraphPad Prism.

Calcium Transient Analysis of iPSC-aCMs

At day 18 of culture post-replating, calcium transient analysis using Fluo-4/AM (Invitrogen, Waltham, MA) was performed (78). The Fluo-4/AM stock solution (50 μg/mL) was prepared in dimethyl sulfoxide (DMSO) containing 2.5% (w/v) Pluronic® F127 (Millipore Sigma, Burlington, MA). Then, the Fluo-4/AM working solution was prepared in Tyrode's solution at 5 μM. Next, culture medium was removed from the cell culture wells and the wells were washed with 1 mL of Tyrode's solution. After removal of Tyrode's solution, the prepared 1 mL Fluo-4/AM working solution was added to wells and incubated at 37°C for 20 minutes. Then, the dye solution was removed, and 1 mL of Tyrode's solution was added to the cells again. The well plate was incubated at 37°C again for 15 minutes. The wells were then washed with 1 mL Tyrode's solution. Finally, the prepared plate was brought to the cell culture incubator (37°C and ~5% CO_2) installed on a confocal fluorescent microscope (Olympus FV3000). By using the GFP filter and a 60x objective lens, the changes in fluorescent signal in iPSC-aCMs were monitored and recorded at 202 frames per second (fps) as time-series TIFF images for 10 seconds. The acquired image sequence was analyzed by a custom calcium transient profiling MATLAB code (included in the Appendix here). This MATLAB code provides important calcium transient (CaT) analysis parameters including CaT amplitude (amplitude=peak signal F/baseline signal F_0), contraction rise time to 10% peak and 90% peak, relaxation decay time to 10% peak and 90% peak, and decaying tau constant. The first four CaT peaks from four different cells were analyzed (n=16) for each condition.

Contraction Movement Analysis of iPSC-aCMs

For the quantitative analysis of the contraction profiles of iPSC-aCMs, the beating motion of iPSC-aCMs was digitally documented as sequential TIFF image series (at 2048 x 2048 pixels of image resolution) using an Olympus IX83 microscope at 10 fps for 10 seconds under 20x magnification with phase contrast mode at day 14 after replating. The vectorial information of contraction movement of iPSC-aCMs in the acquired image sequences was digitally analyzed and extracted via a published MATLAB code: Motion-GUI (20,54). In the MATLAB code operations, the experimental parameter settings were as follows: i) speed/velocity mode analysis, ii) 10 fps, iii) 32 pixels as macroblock size, iv) 8 pixels as maximum detectable movement, v) 0.5 as moving threshold, and vi) 1.3 of minimum brightness. By setting 32 pixels as a macroblock size, the original images were converted into 64 × 64 segments and then analyzed. The acquired time-series vector field information in 64 × 64 segments was visualized as a vector field map. The averaged vector information in 64 × 64 macroblocks were transferred to Microsoft Excel to transform the field data indicating spatial vector information at each coordinate of the TIFF series image into a single column data. Finally, the zero values were removed from the prepared column data for further statistical analysis in GraphPad Prism.

Traction Force Microscopy (TFM) to Quantitate Contractile Forces of iPSC-aCMs

For the quantitative analysis of the contraction force profiles of iPSC-aCMs, traction force microscopy (TFM) was executed using fluorescent bead (0.2 μm in diameter, red fluorescent, excitation/emission: 580 nm/ 605 nm)-embedded soft hydrogel-coated glass bottom 24 well plates exhibiting 25 kPa Young's modulus (Easy Coat™ Plate, Matrigen, Irvine, CA) (28). To modify the hydrogel surface, 50 μg/mL of fibronectin solution (in 1x DPBS) was applied to the soft well plate a day prior to cell seeding and incubated at 4°C overnight. At day 18 of culture post-replating, PC containing iPSC-aCMs and ACFs and RM containing only iPSC-aCM were dissociated from the well plate using TrypLE Express (Thermo-Fisher) and Liberase (Millipore Sigma) as described in the methods of the main manuscript. The dissociated cells were seeded in CMM containing 1% penicillin/streptomycin (P/S) and 10% fetal bovine serum (FBS) and replated onto the fibronectin-coated soft well plates at a density of 0.1×10^6 cells/mL. After 48 hours, the culture medium was replaced with CMM-TID/FA media (recipe described in the methods of the main manuscript) containing 2% FBS. For the remaining cultivation period, the media was changed to CMM-TID/FA media without FBS every other day. The iPSC-aCMs started to beat on the hydrogel surfaces after ~7 days. The beating motions of iPSC-aCMs and the displacement motion of fluorescent beads in the hydrogel were monitored and recorded as time-series TIFF images (45 frames per field of view) in the cell culture incubator (37°C and ~5% CO_2) installed on a confocal fluorescent microscope using a 20x objective lens and RFP channel. The pixel size of acquired image was 0.313 μm/pixel and the frame rate was 7.95 fps. Based on these parameters, the acquired TIFF images were computationally analyzed by a published MATLAB code (28), which provides the cardiomyocytes' traction force information by using the measured bead displacements and Young's modulus of the cell-laden substrate (25 kPa).

Analysis of Multi-nucleation Rate of iPSC-aCMs

For quantitative analysis of multi-nucleation frequency in iPSC-aCMs, iPSC-aCMs were cultured in RM and PC culture formats for 21 days. The cells were fluorescently stained with DAPI (DAPI channel), Alexa Fluor® 488-labeled WGA (GFP channel) and anti-α-actinin antibody (Cy5 channel) for the visualization of nuclei, cell boundary and sarcomere, respectively. Then, multiple regions of interests (318 x 318 μm^2) per two wells (24 well plates) were randomly selected and Z-stacks were imaged via confocal microscopy. The number of single-nucleated cells and multi-nucleated cells were counted manually by two operators separately to verify the counts.

Analysis of Changes in Tissue Thickness

At day 21 of culture post-replating, RM and PC were fixed via 4% PFA and fluorescently stained with DAPI, Alexa Fluor® 488-labeled WGA, and anti-α-actinin antibody. Then, 10 series of z-stack confocal images (from 6 to 29 slices per file, at 1 μm step size) of iPSC-aCMs in RM and PC were randomly selected and analyzed for their height profiles using the Z-axis profile algorithm in NIH ImageJ (79). In each Z-stack image, 3 channels (Cyan, Green and Far-red) were merged into a single channel to simplify the quantification processes. The Z-axis profiling algorithm of ImageJ quantifies the mean signal intensity per unit area of each slice by dividing integrated fluorescent signal density of each slice by its image size (318× 318 μm^2). The data shows the distributions of mean fluorescent signal per slice along the Z-axis. By analyzing the signal

distributions along the Z-axis, the tissue thickness information was determined. The acquired Z-stack profiles from 10 images per condition were then averaged and plotted using GraphPad Prism.

Automated Patch Clamp Recording

Automated patch clamp recordings were performed to assess sodium and calcium currents using the Syncropatch i384 (Nanion Technologies, Germany) with single-hole, 384-well thin-glass recording chips. For both sodium and calcium currents, the internal solution contained 110 mM CsF, 10 mM CsCl, 10mM NaCl, 10 mM HEPES, 20 mM EGTA, and 4 mM ATP-Mg with the final pH adjusted to 7.2 with NaOH. Comparatively, the external solution contained 140 mM NaCl, 4 mM KCl, 2 mM $CaCl_2$, 1 mM $MgCl_2$, 10 mM HEPES, and 5 mM glucose. Pulse generation and data collection were performed using the PatchController384 V.1.9.7 software (Nanion Technologies). Whole cell currents were recorded at RT, filtered at 3 kHz, and acquired at 10 kHz. Both access resistance and apparent membrane capacitance were estimated utilizing built-in protocols. Access resistance compensation was set to 80% while leak and capacitance artifacts were removed using the P/4 method. Peak whole-cell sodium currents were recorded during 500 ms depolarizing pulses from -120 mV to 60 mV (holding potential -120 mV) with readings acquired stepwise every 10 mV switching every 7.5 seconds. This was followed by a 20 ms step to -20 mV and currents were assessed to determine voltage dependance of inactivation. Next, whole cell calcium currents were assessed from a holding potential of -120 mV. Sodium currents were first inactivated with a 2000 ms pulse to -80 mV followed by an 800 ms ramp pulse to -50 mV prior to each test pulse. Calcium currents were then obtained with 500 ms pulses ranging from -80 mV to 50 mV in 10 mV steps, ramping every 10 sec.

Protein Isolation and Western Blotting (WB)

Western blots were executed as previously described (54). Briefly, cells were disassociated and iPSC-aCMs were sorted with magnetic-activated cells sorting (MACS) as described in the methods of the main manuscript and then lysed with 250 µL of 1x RIPA buffer with protease and phosphatase inhibitors (G-Biosciences, St. Louis, MO). Each well was kept separate for protein isolation. Lysate concentrations were determined via the bicinchoninic acid assay (BCA) and were diluted with 4x Laemmli buffer (Bio-Rad, Hercules, CA) with 10% 2-mercaptoethanol (Millipore Sigma). Next, 25 µg of protein per sample was loaded into a 10% SDS-PAGE gel and size fractioned. The resolved gels were then transferred onto nitrocellulose membranes. The membranes were then blocked with 5% BSA in Tris-buffered saline with 0.1% Tween 20 detergent (TBST) for 1 hour at RT. To assess mitochondrial proteins, total OXPHOS human WB antibody cocktail (Abcam) was utilized by diluting it 1:1000 in TBST with 2.5% BSA and incubating at 4°C overnight. The blots were then incubated with an anti-mouse HRP secondary antibody for 1 hour and developed using the Pierce ECL Western Blotting Substrate (Thermo-Fisher) per the manufacture's protocol. The blots were imaged on a C280 imaging system (Azure Biosystems, Dublin, CA). Protein was quantified based on intensity using ImageJ and normalized to the corresponding β-actin signal.

Transmission Electron Microscopy (TEM)

PC and RM cultures were washed with DPBS without calcium or magnesium followed by the addition of 2.5% glutaraldehyde in 0.1 M Sorensen's sodium phosphate buffer (SPB, pH 7.2), prewarmed to 37°C, and incubated for 1 hour at RT. After several washes with SPB, the fixed cells were scraped into a microcentrifuge tube containing SPB, followed by centrifugation at 2500×g for 10 minutes. The resulting pellet was then dislodged and allowed to further fix with 1% osmium tetroxide in SPB for 1 hour at RT. The fixative was then replaced with 1% glutaraldehyde and 4% paraformaldehyde solution in SPB. The fixed samples were washed with SPB multiple times and then dehydrated in ascending concentrations of ethanol leading to 100% absolute ethanol, followed by two changes in propylene oxide (PO) transition fluid. The dehydrated samples were infiltrated overnight in a 1:1 mixture of PO and LX-112 epoxy resin (Ladd Research Industries, Burlington, VT), and 3 hours in 100% LX-112 resin. Then the resin-treated specimens were placed in a 60°C oven to polymerize for 3 days. After the polymerization, semi-thin sections (0.5-1.0 µm) were cut and stained with 1% Toluidine blue-O to confirm the areas of interest via light microscopy (LM). Ultra-thin sections (70-80 nm) were then cut using a Leica Ultracut UCT ultramicrotome, collected onto 200-mesh copper grids, and contrasted with 6% uranyl acetate and Reynolds' lead citrate stains. Specimens were then examined by using a JEOL JEM-1400 Flash transmission electron microscope (JEOL USA Inc., Peabody, MA) at 80 kV operation voltage. Micrographs were acquired using an AMT Side-Mount Nano Sprint Model 1200S-B camera loaded with AMT Imaging software V.7.0.1. (AMT Imaging, Woburn, MA).

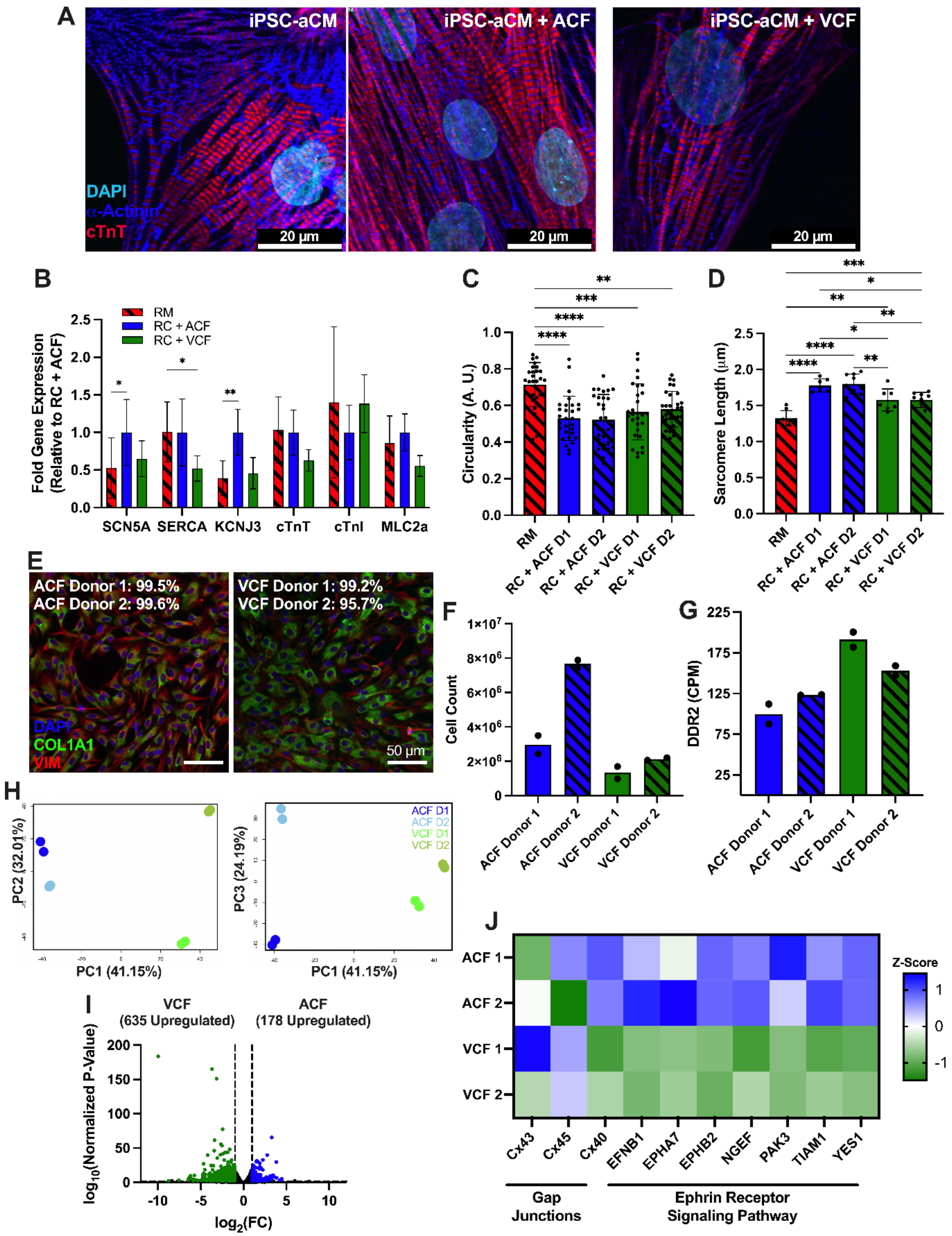

Fig. S1.

Co-culture of iPSC-aCMs with primary adult atrial cardiac fibroblasts (ACFs) or primary adult ventricular cardiac fibroblasts (VCFs). (**A**) Sarcomere visualization via immunostaining for α-actinin and cardiac troponin T (cTnT). Coculture with ACFs or VCFs showed improved

sarcomere organization in iPSC-aCMs over iPSC-aCM monocultures. (**B**) RT-qPCR analysis comparing iPSC-aCM monocultures and iPSC-aCM/ACF and iPSC-aCM/VCF co-cultures indicating that *SCN5A* and *KCNJ3* were significantly increased in iPSC-aCM/ACF co-cultures compared to iPSC-aCM monocultures (n = 8 replicates). RM = randomly distributed iPSC-aCM monocultures; RC = randomly distributed co-cultures of iPSC-aCMs and ACFs or VCFs. (**C**) iPSC-aCM circularity was significantly decreased in both coculture conditions compared to iPSC-aCM monocultures (n = 30 cells for iPSC-aCM/ACF and iPSC-aCM/VCF cocultures and 26 cells for iPSC-aCM monocultures). A.U. = arbitrary units; D1 = donor 1 of corresponding cell type; D2 = donor 2 of corresponding cell type. (**D**) While the iPSC-aCM sarcomere length was significantly increased in both coculture conditions compared to iPSC-aCM monocultures, a significant increase was also observed in the iPSC-aCM/ACF cocultures compared to the iPSC-aCM/VCF cocultures (n = 7 cells for iPSC-aCM/ACF and iPSC-aCM/VCF cocultures donor 1, 9 cells for iPSC-aCM/VCF cocultures donor 2, and 8 cells for iPSC-aCM monocultures). (**E**) Immunostaining of COL1A1 and vimentin (VIM) was performed to determine fibroblast population purity across two donors each of ACFs and VCFs. (**F**) Growth rate over 48 hours was determined for two donors each of ACFs and VCFs with ACFs showing a faster growth rate than VCFs. (**G**) All fibroblast populations were found to express the cardiac fibroblast marker, DDR2, as extracted from RNA-sequencing data (n=2 replicates for each donor and fibroblast type). CPM = counts per million. **(H**) Principal component analysis (PCA) of RNA-sequencing data from the two technical replicates for each of the two donors of ACFs and two donors of ACFs. (**I**) Volcano plot showing the distribution of differentially expressed genes in VCF versus ACF (data averaged for 2 donors of each fibroblast type). (**J**) Heatmap of genes of interest. While connexin 43 (Cx43) and connexin 45 (Cx45) were expressed similarly in the two fibroblast types, connexin 40 (Cx40) was upregulated in ACFs compared to VCFs. Likewise, genes related to the ephrin receptor signaling pathway were upregulated in ACFs compared to VCFs. *$P < 0.05$, **$P < 0.01$, ***$P < 0.001$, ****$P < 0.0001$.

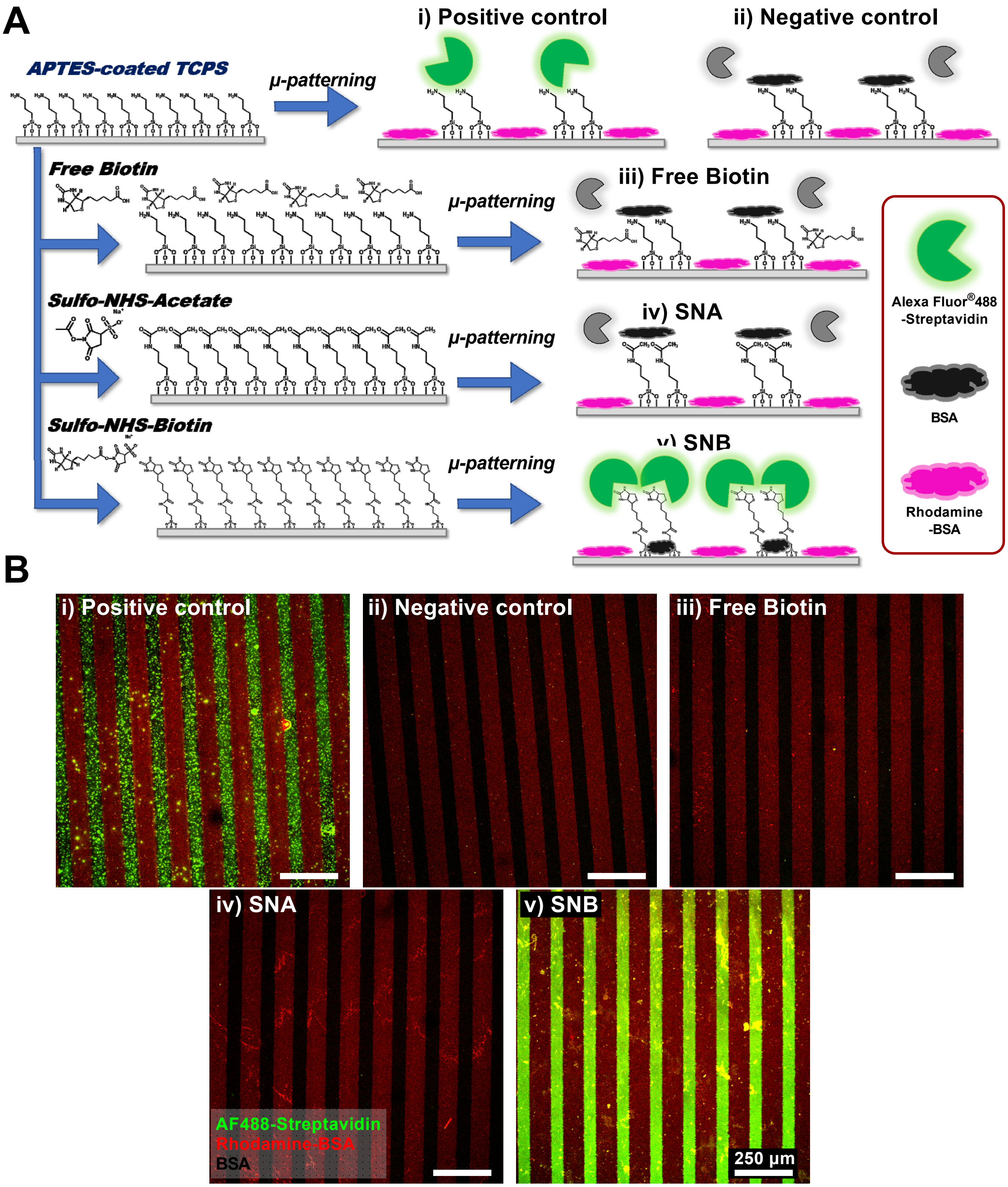

Fig. S2.

Validation of covalent coating and micropatterning of (3-Aminopropyl)triethoxysilane (APTES) on tissue culture polystyrene (TCPS). (A) As shown in Figure 1 of the main manuscript, we treated TCPS with O_2 plasma to activate it with hydroxyl groups, coated with APTES to introduce amine groups on the TCPS, placed a polydimethyl siloxane (PDMS) mask on

it, exposed to O_2 plasma to ablate APTES from unprotected regions to expose bare TCPS, flowed in rhodamine (red)-labeled bovine serum albumin (rhodamine-BSA) with the PDMS mask on to coat the bare TCPS regions with BSA, and then removed the PDMS mask to expose the alternating patterns of APTES-coated and BSA-coated TCPS. To demonstrate the alternating pattern, we subsequently treated for 30 minutes the APTES/rhodamine-BSA patterns generated as described above with AF488 (green)-Streptavidin (SA), diluted at 20 µg/mL in 1x DPBS containing 0.1% Tween20 and 0.3% BSA (followed by washing in 1X DPBS); the AF488-SA adsorbed non-specifically to the patterned APTES regions but *not* to the rhodamine-labeled BSA regions as shown schematically in (i) of panel A (BSA is depicted by the cloud symbol while AF488-SA is depicted by the Pacman symbol; green for immobilization to the surface) and fluorescent image in (i) of panel B. In a second instance, we repeated the steps of the first instance above except that we first treated the APTES/rhodamine-BSA patterns with unlabeled BSA and then incubated with AF488-SA, which did not absorb to *any* parts of the surface as shown schematically in (ii) of panel A and fluorescent image in (ii) of panel B since the BSA (labeled and unlabeled) was coated everywhere. The only color seen in (ii) of panel B is that of the rhodamine-BSA patterns. In a third instance, we first incubated the APTES-coated surface with free biotin (20 mM) for 1 hour at RT and washed with water *prior to* placing the PDMS mask on it and executing the steps in the second instance above. The AF488-SA did not absorb much to any parts of the surface as shown schematically in (iii) of panel A and fluorescent image in (iii) of panel B since free biotin cannot strongly bind BSA nor TCPS and since the BSA (labeled and unlabeled) was coated everywhere. We saw some minor green signal in (iii) of panel B suggesting that some free biotin may not be fully washed out but otherwise, majority of the color seen in (iii) of panel B is that of the rhodamine-BSA patterns. In a fourth instance, we first incubated the APTES-coated surface with sulfo-N-Hydroxysuccinimide ester (NHS)-acetate (SNA, 20 mM) for 1 hour at RT and washed with water *prior to* placing the PDMS mask on it and executing the steps in the second instance above. The SNA binds to the APTES via amine groups. The AF488-SA did not absorb much to any parts of the surface as shown schematically in (iv) of panel A and fluorescent image in (iv) of panel B since the BSA (labeled and unlabeled) was coated everywhere and AF488-SA does not bind to the acetate groups of SNA. In a final and fifth instance, we first incubated the APTES-coated surface with sulfo-NHS-biotin (SNB, 20 mM) for 1 hour at RT and washed with water *prior to* placing the PDMS mask on it and executing the steps in the second instance above. The SNB binds to the APTES via amine groups. The AF-488-SA adsorbed strongly to the biotin-modified APTES patterns (even with some BSA blocking) but not to the rhodamine-BSA regions as shown schematically in (v) of panel A and fluorescent image in (v) of panel B. This AF-488-SA staining pattern in (v) was even stronger than that of (i) in panel B since some AF-488-SA binding was inhibited by the 0.3% BSA present in the incubation solution. Since TCPS does not contain amine groups and the source of the amine groups is APTES alone, we conclude from results above that APTES was successfully patterned onto the surface and provided the amine groups necessary for fibronectin conjugation.

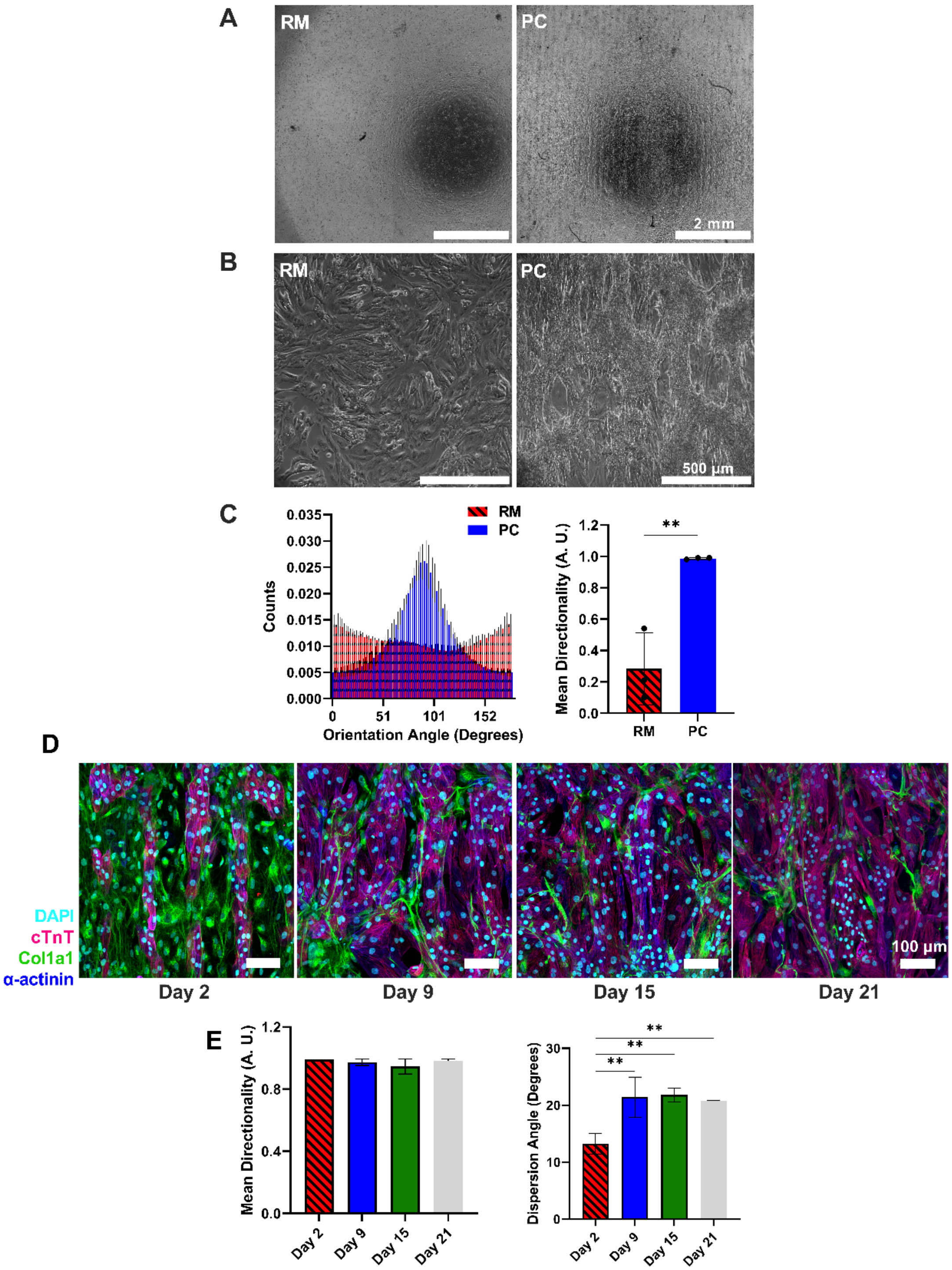

Fig. S3.

iPSC-aCM alignment. (**A**) Low magnification (6.5 mm x 6.5 mm) phase contrast images of random iPSC-aCM monocultures (RM) and patterned co-culture (PC) of iPSC-aCMs and primary adult atrial cardiac fibroblasts (ACFs) to visualize improved alignment in PC. (**B**) Phase contract images of RM and PC from another iPSC-aCM donor. (**C**) Histogram (left) of orientation angles of cells in RM and PC. PC showed nearly perfect alignment (right) as compared to RM based on

mean directionality (n = 3 images per platform). A.U. = arbitrary units. (**D**) PC fluorescent images over 21 days in culture. The iPSC-aCMs are stained for cardiac troponin T (cTnT) and α-actinin, while the ACFs are stained for collagen 1a1 (Col1a1). (**E**) Mean directionality in PC is maintained at similar levels over time while the dispersion angle increases after day 2 and remains similar afterwards for the 21 days in culture (n = 3 images per timepoint). $^{**}P < 0.01$.

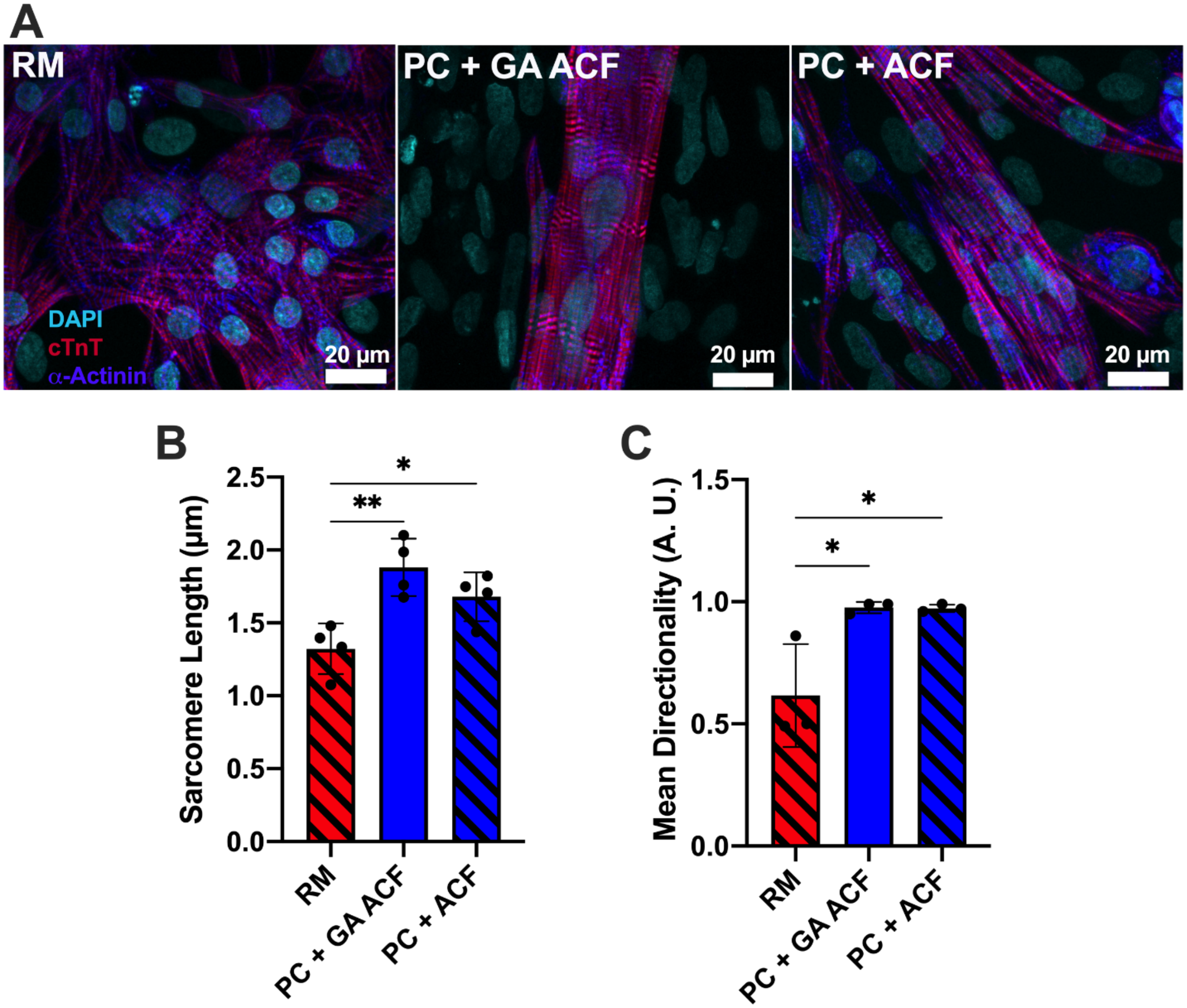

Fig. S4.

Effects of mitomycin C treatment of atrial fibroblasts on iPSC-aCM phenotype in patterned coculture (PC). (**A**) Staining of iPSC-aCMs in random monoculture (RM) and PC with either mitomycin C growth-arrested primary adult atrial cardiac fibroblasts (GA ACF) or non-growth-arrested ACFs. Cells are stained for DAPI, cardiac troponin T (cTnT), and α-actinin. (**B**) Quantification of sarcomere length of cells in each condition (n = 4 cells per condition). (**C**) Quantification of cell directionality in each condition (n = 3 images per condition). *$P < 0.05$, **$P < 0.01$. A.U. = arbitrary units.

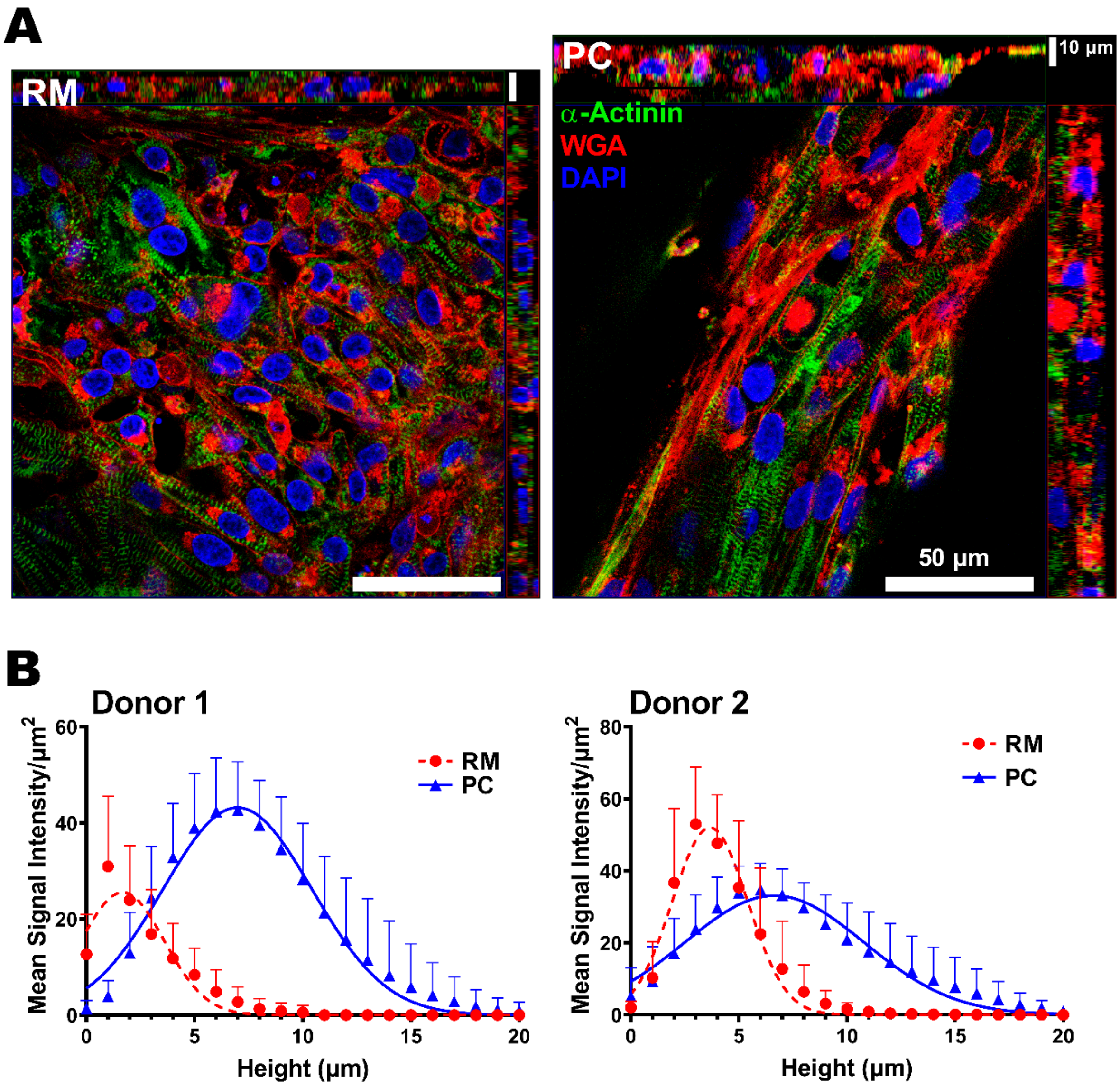

Fig. S5.

Three-dimensional reconstruction of patterned coculture (PC) of iPSC-aCMs and primary adult atrial cardiac fibroblasts (ACFs) and random iPSC-aCM monocultures (RM). (**A**) Three dimensional images of iPSC-aCMs stained for α-actinin, WGA, and DAPI showing that PC has an increased thickness (height) as compared to RM. Cross-sectional view is shown at the top and right of each wide field image. (**B**) Quantification of changes in distribution of tissue height comparing RM to PC. The graph shows the distribution of mean fluorescent signal intensity per unit area (μm^2) along the Z-axis of the Z-stack images (n = 10 series of Z-stack images per condition; see supplemental methods for additional details on quantification).

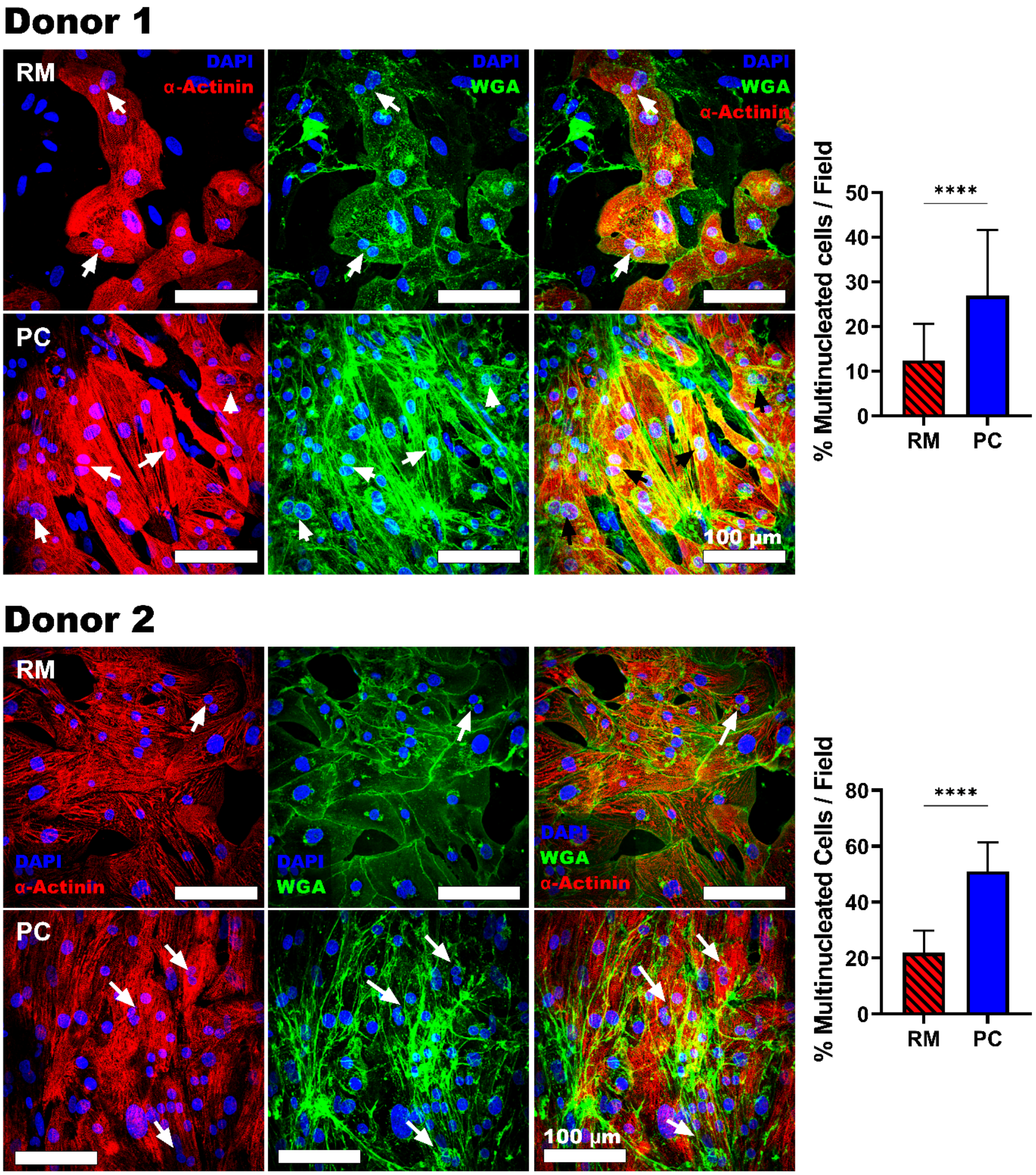

Fig. S6.

Multinucleation in iPSC-aCMs in patterned co-culture (PC) of iPSC-aCMs and primary adult atrial cardiac fibroblasts (ACFs). The iPSC-aCMs were identified via DAPI and α-actinin while cell boundaries were visualized using WGA. Both iPSC-aCM donors (top panel: donor 1, bottom panel: donor 2) showed an increase in multinucleated cells in PC compared to RM (n >180 cells per condition and per iPSC-aCM donor). Arrow indicates multinucleated cells. Scale bar size applies to all images (100 μm). ****$P < 0.0001$.

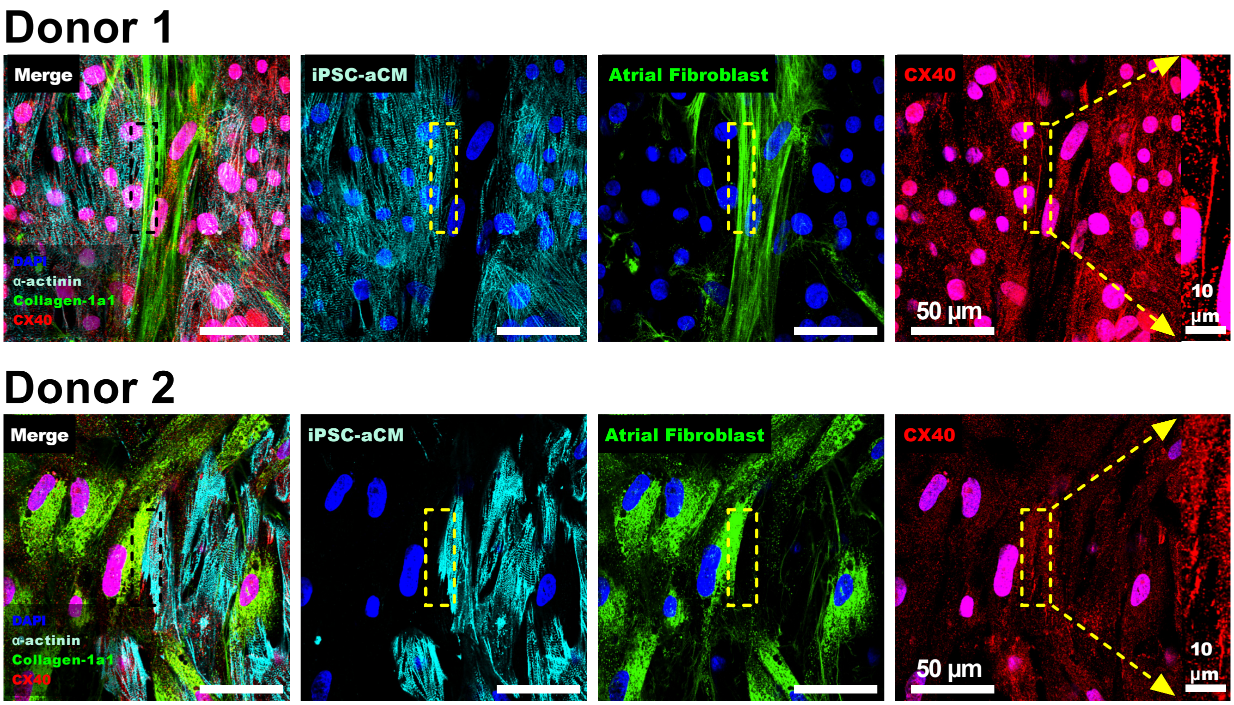

Fig. S7.

Connexin 40 (Cx40) localization at the interface between iPSC-aCMs and primary adult atrial cardiac fibroblasts (ACFs) in patterned co-culture (PC). The iPSC-aCMs (top panel: donor 1, bottom panel: donor 2) were identified via α-actinin staining (cyan) while the ACFs were identified via collagen 1a1 (green) staining. Cx40 staining (red) was carried out on the co-cultures and is present in both cell types. In the Cx40 images, the right panel shows magnified regions indicated by the dotted rectangles in the corresponding left images.

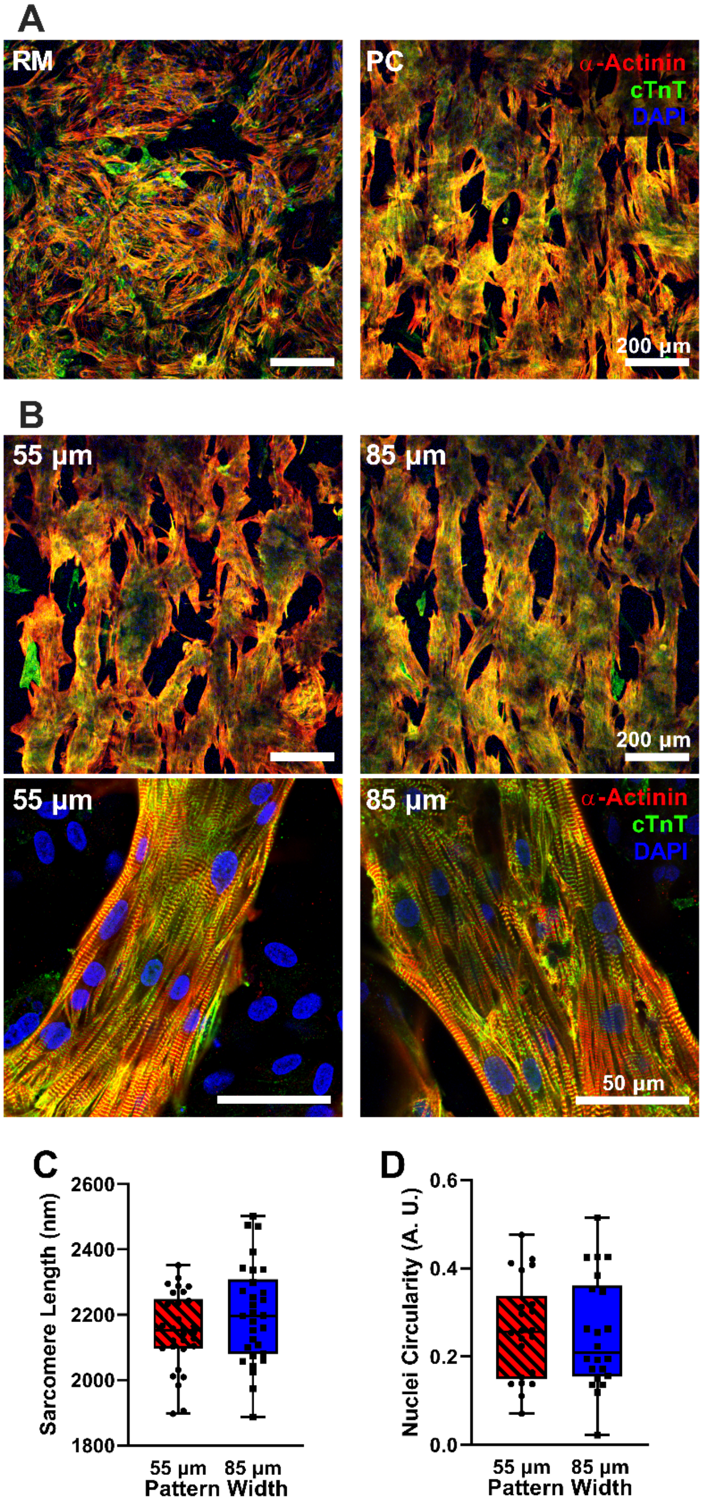

Fig. S8.
Fibronectin patterns of different widths (55 µm and 85 µm) for iPSC-aCM alignment. (A) Low magnification view of random iPSC-aCM monocultures (RM) and patterned coculture (PC) of iPSC-aCMs and primary adult atrial cardiac fibroblasts (ACFs) immunostained with α-actinin

and cardiac troponin T (cTnT) to visualize overall alignment. (**B**) Fluorescent images of iPSC-aCM patterns on fibronectin lines of 55 μm and 85 μm widths at low magnification (upper panels) and high magnification (bottom panels). The iPSC-aCMs were immunostained with α-actinin and cTnT. Quantified **(C)** sarcomere length and **(D)** nuclei circularity were statistically similar in PC containing either 55 μm or 85 μm pattern widths (n = 22 cells). A.U. = arbitrary units.

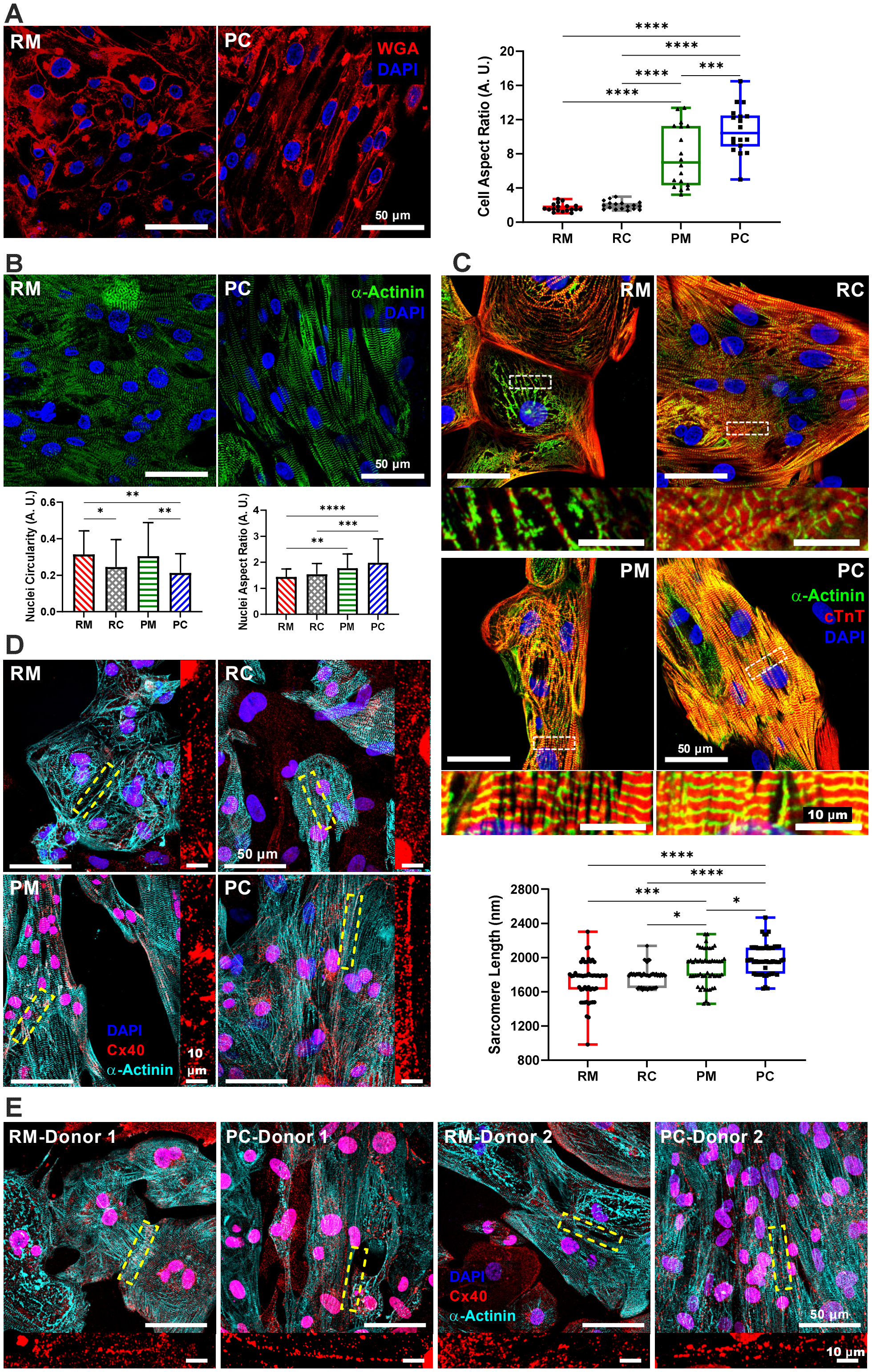

Fig. S9.

Structural maturity of iPSC-aCMs (different donor than in main manuscript) in different culture platforms. (A) WGA membrane stain of random iPSC-aCM monoculture (RM) and

patterned co-culture (PC) of iPSC-aCMs and primary adult atrial cardiac fibroblasts (ACFs). Cell aspect ratio showed a significant increase in patterned iPSC-aCM monocultures (PM) and PC compared to RM and random iPSC-aCM/fibroblast co-cultures (RC). PC also showed a significant increase in cell aspect ratio as compared to PM and RC (n = 18 cells). A.U. = arbitrary units. (**B**) Cell nuclei staining with DAPI and α-actinin to identify iPSC-aCMs. Nuclei circularity showed a significant decrease in PC compared to RM, while the nuclei aspect ratio was significantly increased in PC compared to RM, indicating a more elongated phenotype (PC: n = 47 nuclei; RM: n = 75 nuclei). (**C**) RM, RC, PM, and PC immunostained for cardiac troponin T (cTnT) and α-actinin showing an increase in sarcomere organization, specifically in PC. From these images, the sarcomere length was quantified, showing a significant increase in PM and PC compared to RM; however, the longest sarcomere length was in PC, which was significantly longer than the other three platforms (n = 52 sarcomeres). (**D**) Connexin 40 (Cx40) staining in RM and PC. Right images are magnified and rotated regions indicated by the dotted rectangles in the corresponding left images. (**E**) Connexin 43 (Cx43) staining in RM and PC from two iPSC donors. Bottom images are magnified and rotated regions indicated by the dotted rectangles in the corresponding top images. $*P < 0.05$, $**P < 0.01$, $***P < 0.001$, $****P < 0.0001$.

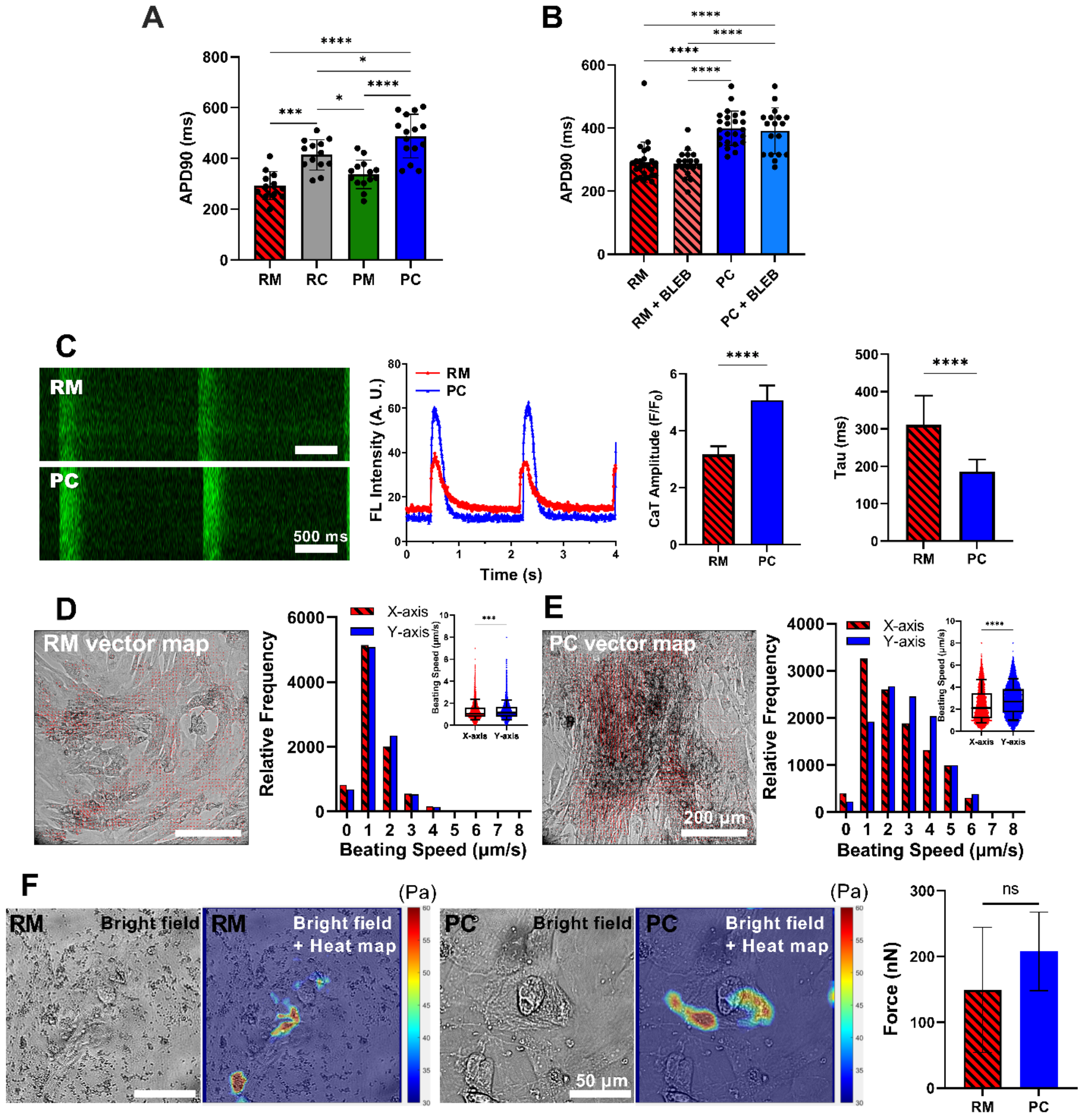

Fig. S10.

Electrical and contraction force maturity of iPSC-aCMs (different donor than in main manuscript) in different culture platforms. (**A**) Action potential duration (APD_{90}) quantification via optical voltage mapping (OVM) indicating a significant increase in patterned iPSC-aCM/ACF co-culture (PC) compared to random iPSC-aCM/ACF co-culture (RC), random iPSC-aCM monoculture (RM), and patterned iPSC-aCM monoculture (PM) (n=12 cells). (**B**) Cell treatment with a myosin II inhibitor blebbistatin (BLEB) at 10 µM for 1 hour in Tyrode's solution (80) had no statistical effect on the OVM data, indicating that the OVM data is not confounded by artifacts of cell movement (n=18 cells for RM and 24 cells for PC). (**C**) Calcium imaging indicates an increase in CaT amplitude and a decrease in decaying tau (n=16, 4 first beatings for 4 different

cells per culture condition) for PC compared to RM. A.U. = arbitrary units. (**D**) Vector analysis results for iPSC-aCMs in RM. Left image depicts registered vector field map. Right histogram compares intensity distribution of contraction intensity between x- and y-axis components (inset box chart shows direct comparison of x- and y- components). (**E**) Vector analysis results for PC. Left image depicts registered vector field map. Right histogram compares intensity distribution of contraction intensity between x- and y-axis components (inset box chart shows direct comparison of x- and y- components). (**F**) Mean contractile force comparison for iPSC-aCMs previously cultured in RM or PC using traction force microscopy (TFM) analysis on fluorescent bead-embedded 25 kPa hydrogel surfaces. Left panels show brightfield images and its overlay images with traction heat maps for iPSC-aCMs adhered to hydrogel surfaces and previously cultured in RM or PC. Right panel shows quantitative comparison of acquired mean contractile forces for iPSC-aCMs on hydrogel surfaces and previously cultured in RM or PC. *$P < 0.05$, ***$P < 0.001$, ****$P < 0.0001$.

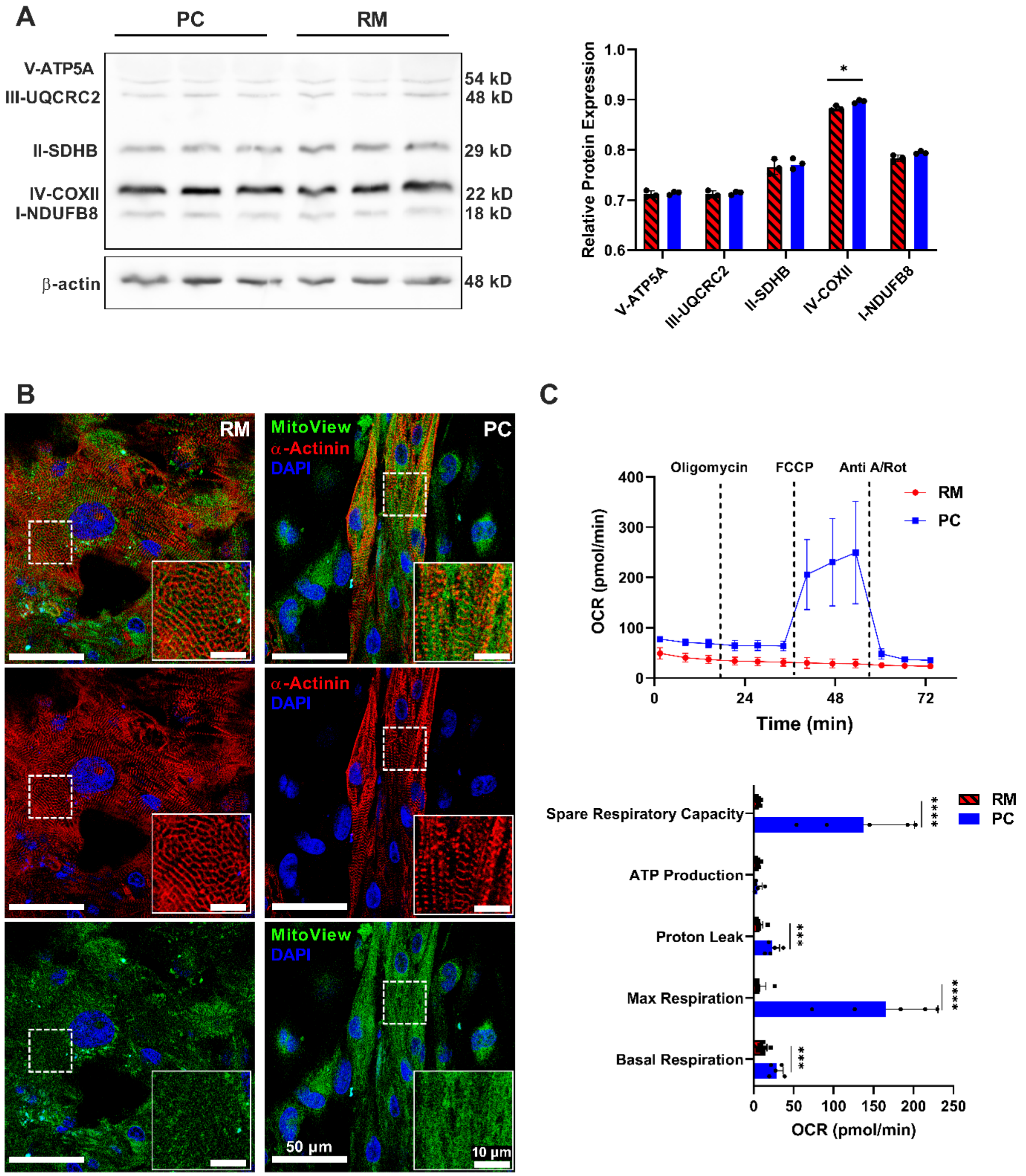

Fig. S11.

Metabolic maturity of iPSC-aCMs in different culture platforms (**A**) Western blot analysis for mitochondrial complexes in random iPSC-aCM monoculture (RM) and patterned coculture (PC) of iPSC-aCMs and primary adult atrial cardiac fibroblasts (n=3 replicates). Right panel depicts the quantitation of image on left. (**B**) Immunostaining for mitochondria via MitoView and sarcomeres via α-actinin shows that while the mitochondria are randomly distributed in RM, they are linear and aligned with the sarcomeres in PC (different donor than in main manuscript). (**C**) Seahorse

analysis measuring oxygen consumption rate (OCR) of RM and PC (different donor than in main manuscript). Based on this analysis, additional parameters could be calculated, which revealed a significant increase in spare respiratory capacity, max respiration, and basal respiration in PC compared to RM (PC: n = 5 wells; RM: n = 8 wells). *$P < 0.05$, ***$P < 0.001$, ****$P < 0.0001$.

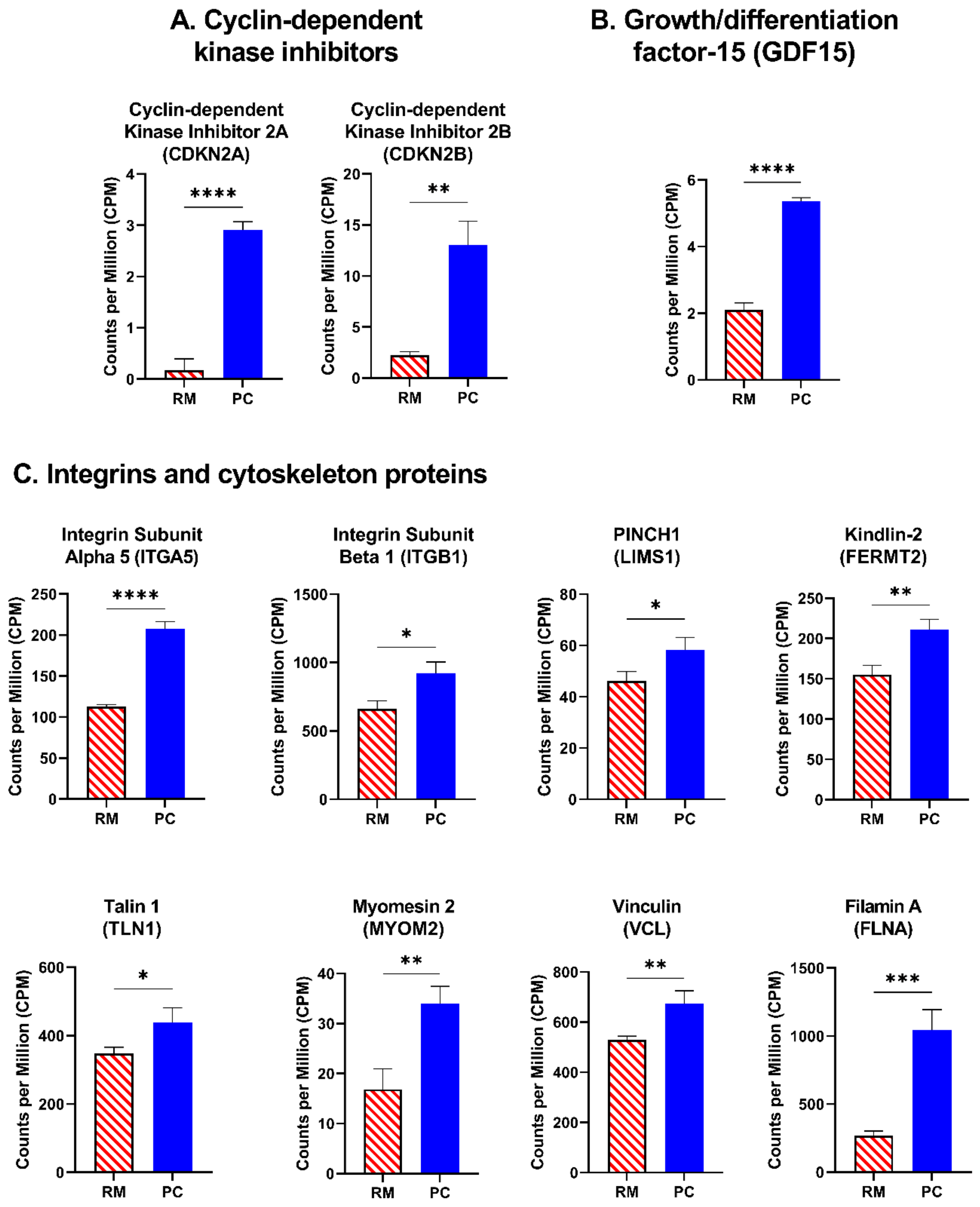

Fig. S12.

Additional genes of interest from RNA sequencing analysis. (**A**) *CDKN2A* and *CDKN2B* expression comparison between random iPSC-aCM monoculture (RM) and patterned coculture (PC) of iPSC-aCMs and primary adult atrial cardiac fibroblasts (n = 3 sequencing replicates). (**B**) GDF15 expression comparing RM to PC (n = 3 sequencing replicates). (**C**) Comparison of gene expressions related to integrin/cytoskeleton proteins (*ITGA5*, *ITGB1*, *LIMS1*, *FERMT2*, *TNL1*, *MYOM2*, *VCL*, and *FLNA*) for RM and PC (n = 3). *$P < 0.05$, **$P < 0.01$, ***$P < 0.001$, ****$P < 0.0001$.

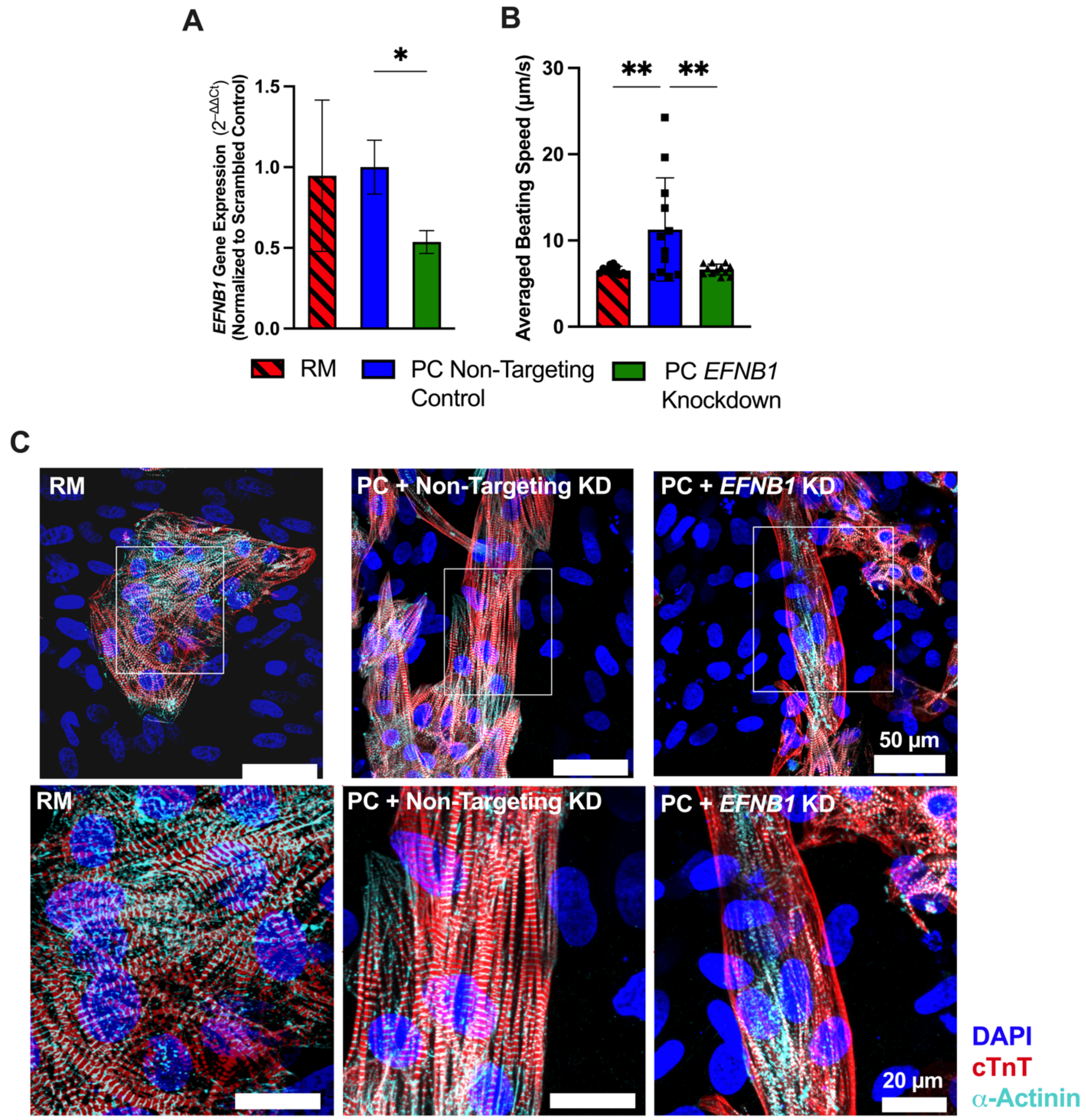

Fig S13.

Knockdown of ephrin B1 expression in patterned co-culture (PC) of primary adult atrial fibroblasts (ACFs) and iPSC-aCMs. (**A**) Verification of ephrin B1 (*EFNB1*) knockdown via gene expression analysis in PC 8 days following transfection with siRNA targeting *EFNB1* or non-targeting siRNA control (n = 6). Expression in random iPSC-aCM monoculture (RM) is shown for comparison to the PC conditions. Knocking down *EFNB1* (**B**) lowers the speed of iPSC-aCM contraction movement (n = 12) and (**C**) disrupted/disorganized sarcomeres in iPSC-aCMs as visualized via immunostaining of cardiac troponin T (cTnT) and α-actinin. Bottom images in panel (C) are zoomed-in images for the corresponding rectangles in the top images. KD = knockdown.

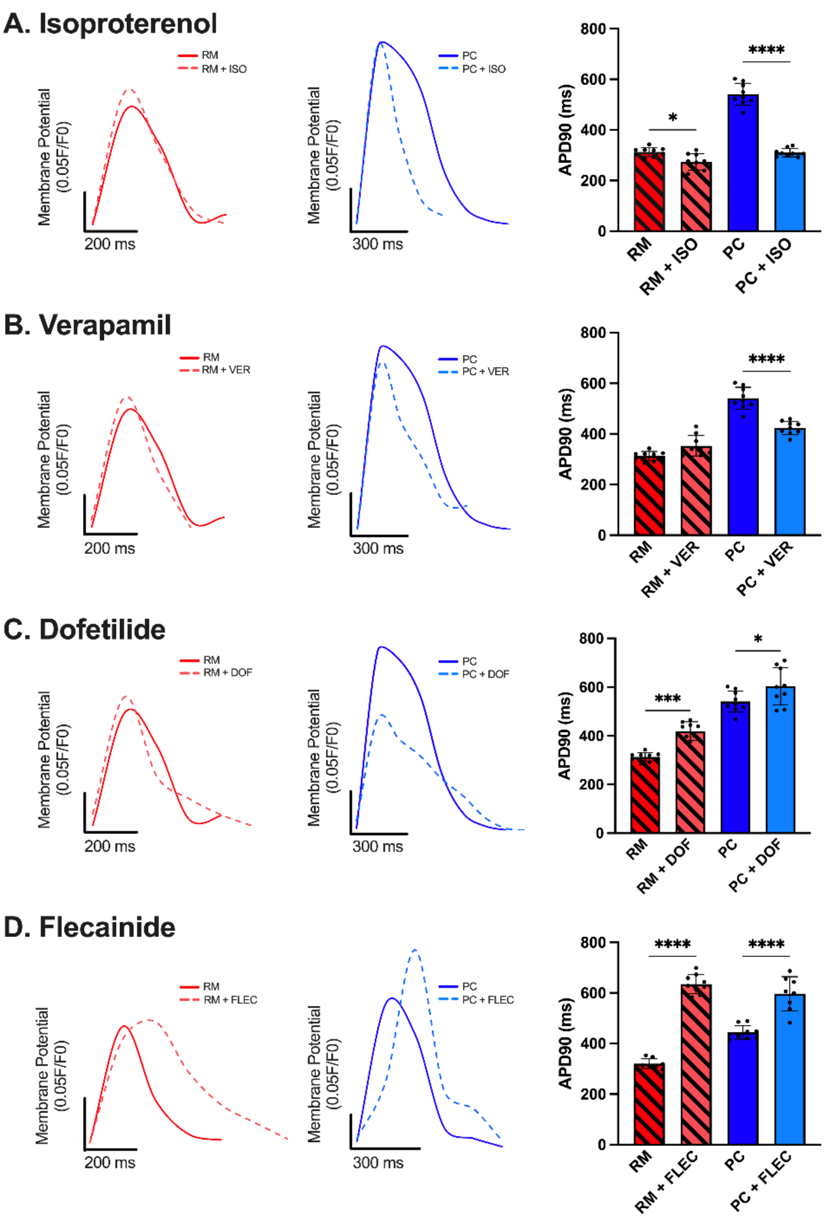

Fig. S14.

Prototypical drug response of iPSC-aCMs (different donor than in main manuscript) in different culture platforms. (A) Isoproterenol (ISO) treated random iPSC-aCM monoculture (RM) and patterned coculture (PC) of iPSC-aCMs and primary adult atrial cardiac fibroblasts showed a statistically significant decrease in action potential duration (APD_{90}) relative to vehicle (DMSO) treated control cultures; the relative decrease in APD_{90} was higher in PC compared to RM. **(B)** PC showed a significant decrease in APD_{90} when treated with verapamil (VER), while no difference was observed in RM. **(C)** Both RM and PC showed a significant increase in APD_{90} in response to dofetilide (DOF). **(D)** Treatment with flecainide (FLEC) led to increases in APD_{90} for both RM and PC. All panels: n = 9 cells. $^{*}P < 0.05$, $^{***}P < 0.001$, $^{****}P < 0.0001$.

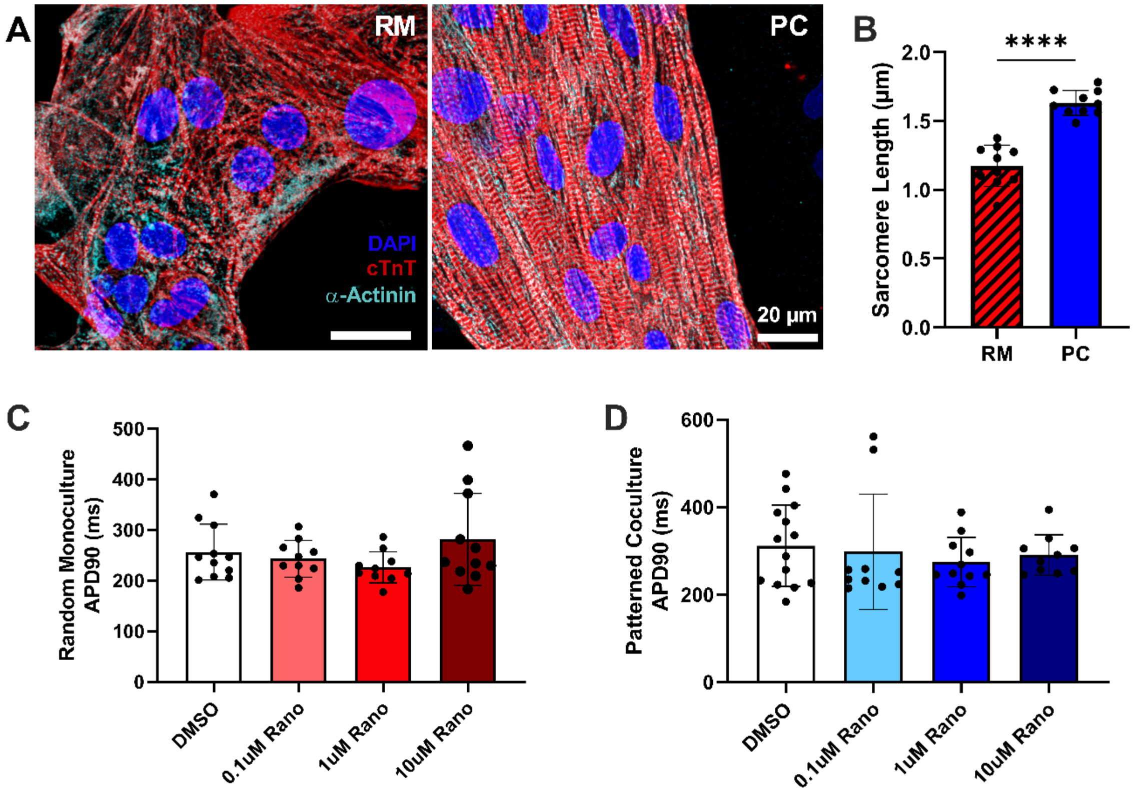

Fig. S15.

Mutant (E428K in *SCN5A*) iPSC-aCM maturation and drug response in patterned co-culture (PC) of iPSC-aCMs and adult primary atrial cardiac fibroblasts. (**A**) Sarcomere visualization of mutant iPSC-aCMs cultured in random monoculture (RM) or PC. Cells are stained for DAPI, cardiac troponin T (cTnT), and α-actinin. (**B**) Quantification of sarcomere length of cells imaged in A. PC shows significantly longer sarcomeres than RM (n = 10 cells per condition). Action potential duration (APD_{90}) measured by Optical Voltage Mapping of mutant iPSC-aCMs cultured in **(C)** RM or **(D)** PC and treated with varying concentrations of ranolazine. ****P < 0.0001.

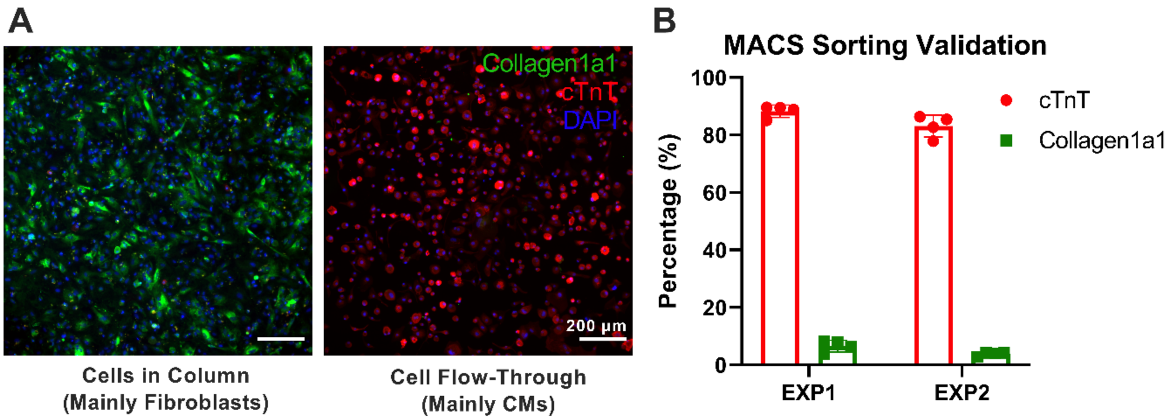

Fig. S16.

Validation of Magnetic activated cell sorting (MACS). After negative sorting for non-cardiomyocytes, the separated cells were plated and immunostained for (**A**) Collagen 1a1, cardiac troponin T (cTnT), and DAPI to determine the percentage of fibroblasts and cardiomyocytes. (**B**) The flow through cell population was quantified to determine cardiomyocyte purity indicating improved enrichment of cardiomyocytes after MACS sorting.

Movie S1.
Calcium imaging of iPSC-aCMs in random monoculture (RM).

Movie S2.
Calcium imaging of iPSC-aCMs in patterned coculture (PC) with primary adult atrial cardiac fibroblasts.

Movie S3.
Beating iPSC-aCMs in RM.

Movie S4.
Beating iPSC-aCMs in PC.

Movie S5.
Beating iPSC-aCMs in RM with vector map. The red arrows indicate direction and magnitude of vector profile in the corresponding Macroblock segments.

Movie S6.
Beating iPSC-aCMs in PC with vector map. The red arrows indicate direction and magnitude of vector profile in the corresponding Macroblock segments.

Movie S7.
Beating iPSC-aCMs previously cultured in RM, dissociated, and subsequently cultured onto red fluorescent bead-containing hydrogels (25 kPa) for traction force microscopy (TFM) analysis. Left panel shows the merged videos from brightfield and red fluorescent protein (RFP) channel, and right panel shows the traction heat map video.

Movie S8.
Beating iPSC-aCMs previously cultured in PC, dissociated, and subsequently cultured onto red fluorescent bead-containing hydrogels (25 kPa) for TFM analysis. Left panel shows the merged videos from brightfield and RFP channel, and right panel shows the traction heat map video.

MATLAB™ Code for Calcium Transient Parameter Analysis

```
clc;clear;close all;

AcquisitionFrequency = 18;          %This can be changed, maybe UI


addpath('/Users/EmaSpanghero/Documents/MATLAB/bfmatlab')

disp('Please select the tiff file to analyze');
[FileName,PathName] = uigetfile('*.tif;*.tiff');

tiff_stack = bfopen(char(strcat(PathName,FileName)));

fig1 = figure(1);
J = imadjust(tiff_stack{1,1}{1,1});
NumberOfFrames = size(tiff_stack{1,1},1);
imshow(J, 'InitialMagnification','fit')

fig = drawfreehand();
mask = fig.createMask();
close(figure(1))

%% Extraction of intensity profile

cropped=[];
for i = 1:NumberOfFrames
   cropped(:,:,i) = double(tiff_stack{1,1}{i,1}).*mask;
end
cropped(cropped==0) = NaN;

intensity = [];
for i = 1:NumberOfFrames
   intensity(i) = mean(cropped(:,:,i), 'all', 'omitnan');
end
figure(1);
movegui(figure(1),[0 250])
plot(intensity)


%% Contraction isolation

[contr_val,peak_maxes, location_maxes, location_bases, ...
```

```
    offset] = contraction_calculator(intensity, NumberOfFrames);

%% Last peak check

%{
[~,~,~,pr_check] = findpeaks(contr_val(:,end));
[peak_check,loc_check] =
findpeaks(contr_val(:,end),'MinPeakProminence',0.2*max(pr_check));

check = 0;
if length(peak_check)>1
    check = 1;
    loc_check(end+1) = NumberOfFrames-location_bases(end)+offset;
    answer = 'No';
    k = 2;
    while strcmp(answer,'No')
        contr_val(:,end) = 0;
        start_beat = location_bases(end)-offset;
        end_beat = start_beat+loc_check(k)-offset;
        for i = 1:end_beat-start_beat

          contr_val(i,end) = intensity(start_beat+i-1);
        end
        k=k+1;
        figure(2)
        movegui(figure(2),[800 250])
        plot(nonzeros(contr_val(:,end)))
        answer = questdlg('Does the contraction profile look correct?','Last contraction',...
        'Yes','No','Yes');
    end
else
        %do nothing
end
%}
disp('Beat/s segmentation, done!');
fprintf("%i peak/s found at t =\n", length(location_maxes));
disp(location_maxes);



%% Photobleach correction

photobl =questdlg('Do you need to apply photo bleach correction?','Photobleach correction
toggle',...
    'Yes','No ', 'Yes');
if photobl == char('Yes')
```

```
    [CorrectedTrace] = photobleachcorrection(intensity, contr_val,...
        NumberOfFrames);

    figure()
    plot(intensity)
    hold on
    plot(CorrectedTrace)
    photobl = 1;
    intensity = CorrectedTrace;
end
%% Contr_val recalculation if photobleach correction is activated

if photobl == 1
    [contr_val, peak_maxes, location_maxes, location_bases, ...
    offset] = contraction_calculator(intensity, NumberOfFrames);
end
%{
for i = 1: size(contr_val,2)
    figure()
    plot(nonzeros(contr_val(:,i)))
end
%}
%% Elimination of wrongly identified peaks

elim = questdlg('Do you want to remove wrongly identified peaks from the analysis?','Peak
elimination',...
    'Yes','No','No');

switch elim
    case 'Yes'
    prompt = {'\bf \fontsize{12} Please enter the peak number (eg [1 2 3]):'};
    dlgtitle = 'Remove peaks';
    dims = [1 88];
    definput = {'','', '', '','','','',''};
    opts.Interpreter = 'tex';
    RC = [];
    RC = inputdlg(prompt,dlgtitle,dims,definput, opts);
    rem_peak = str2num(RC{1});

    if rem_peak ~= size(contr_val,2)
        temp = [nonzeros(contr_val(:,rem_peak-1)); nonzeros(contr_val(:,rem_peak))];
        for j = 1:location_bases(rem_peak+1)-location_bases(rem_peak-1)
            temp(j) = intensity(location_bases(rem_peak-1)-offset+j);
        end

        contr_temp = [];
```

```
        if length(temp) > size(contr_val,1)
            contr_temp = zeros(length(temp),size(contr_val,2));
            for i = 1:size(contr_val,2)
                if i == rem_peak-1
                    contr_temp(:,i) = temp(:);
                else
                    contr_temp(:,i) = [contr_val(:,i); ...
                        zeros(length(temp)-size(contr_val,1),1)];
                end
            end
            contr_val = [];
            contr_val = contr_temp;
        end
        contr_val(:,rem_peak)=[];
        location_bases(rem_peak) = [];
        location_maxes(rem_peak) = [];
    else
        temp = [nonzeros(contr_val(:,rem_peak-1)); nonzeros(contr_val(:,rem_peak))];
        for i = 1:size(contr_val,1)
            contr_val(i,rem_peak-1) = temp(i);
        end
        contr_val(:,rem_peak) = [];
        location_bases(rem_peak) = [];
        location_maxes(rem_peak) = [];
    end


    disp('Peak elimination completed!');
end


%% Contraction measurements

close all
time_stamps = [];
time_values = [];
magnitude = [];
dim_interp = length(1:0.01:size(contr_val,1));
interpolation= zeros(dim_interp,size(contr_val,2));

for i = 1:size(contr_val,2)

    curr_trace = nonzeros(contr_val(:,i));
    max_local = max(curr_trace);

    points = 1:0.01:length(curr_trace);
```

```
    new_fps = (length(points))/(length(curr_trace)/AcquisitionFrequency); %number of new
frames / total rise time
    curr_interp = interp1(1:length(curr_trace),curr_trace,points);
    interpolation(:,i) = [curr_interp'; zeros(dim_interp-length(curr_interp),1)];

    figure(3)
    %plot(1:length(curr_trace),curr_trace,'o',points,interpolation,':.')
    plot(nonzeros(interpolation(:,i)))
    limits = xlim;
    limits(1) = -500;
    xlim(limits)

    %time_stamps = points x coordinate (time frame)
    %time_values = points y coordinate (intensity value)
    [time_stamps(:,i),~] = getpts(figure(3));
    time_stamps(:,i) = round(time_stamps(:,i));
    time_values(:,i) = interpolation(time_stamps(:,i),i);

    magnitude(i) = max_local-time_values(1,i);

    [C10, C50, C90, t_start] = findcontractionparams(nonzeros(interpolation(:,i)),...
        max_local, magnitude(i));

    %Save params
    c10(i) = C10-t_start; %number of frames
    c50(i) = C50-t_start;
    c90(i) = C90-t_start;
end

c10_time = c10/new_fps*1000; %milliseconds
c50_time = c50/new_fps*1000;
c90_time = c90/new_fps*1000;

C10_avg = mean(c10_time);
C50_avg = mean(c50_time);
C90_avg = mean(c90_time);

close(figure(3))
disp('Contraction parameters measurement, done!')

%% Relaxation isolation and tau measurement


relax_val = zeros(size(interpolation,1),size(interpolation,2));

for i = 1:size(interpolation,2)
```

```
    [~,local_max] = max(interpolation(:,i)); %location where the max is in every contraction
    for j = 1:time_stamps(2,i)-local_max
        relax_val(j,i) = interpolation(local_max+j-1,i);
    end
end

tau = [];
a = [];
b = [];
c = [];

for i = 1:size(relax_val,2)

    temp = nonzeros(relax_val(:,i));
    frames = 1:length(temp);
    [fitresult] = fit_tau(frames,temp);

    tau = [tau; ((1/(-fitresult.b)).*(1/new_fps))*1000]; %ms
    a = [a; fitresult.a];
    b = [b; fitresult.b];
    c = [c; fitresult.c];
end

%Exclude negative tau values
tau(tau<0) = NaN;
tau_avg = mean(tau);

disp('Tau measurement, done!');

%% Verify fitting

%{
%Uncomment to plot the graphs
for curve_n = 1:size(contr_val,2)
    %curve_n = beat that you want to verify the fitting of

    x = 1:nnz(interpolation(:,curve_n));
    [~,loc] = max(interpolation(:,curve_n));
    delta = length(interpolation(:,curve_n))-length(interpolation(loc:end,curve_n));
    y = a(curve_n)*exp(b(curve_n)*(x-delta))+c(curve_n);
    figure()
    plot(x,y, 'LineWidth', 1)
    hold on
    plot(x,nonzeros(interpolation(:,curve_n)), 'Linewidth', 1)
    xlim([1 5000])
    ylim([min(interpolation(:,curve_n)-50) max(interpolation(:,curve_n))+50])
```

```
    saveas(figure(1),'fitting.png')
end

%}

%% Baseline

%Average between the starting points of all contractions
beat_mean = mean(time_values,1);
baseline = mean(beat_mean);
baseline_old = mean(time_values(1,:));

disp('Baseline measurement, done!');

%% Relaxation measurements

r10 = zeros(size(interpolation,2),1);
r50 = zeros(size(interpolation,2),1);
r90 = zeros(size(interpolation,2),1);

for i = 1:size(interpolation,2)

    maximum = relax_val(1,i);
    minimum = time_values(2,i);
    magnitude = maximum-minimum;

    fall_data = nonzeros(relax_val(:,i));
    %points = 1:0.05:length(fall_data);
    %new_fps = (length(points))/(length(fall_data)/AcquisitionFrequency); %number of new
frames / total rise time
    %interpolation = interp1(1:length(fall_data),fall_data,points);
    %interpolation = interpolation';
    %plot(1:length(fall_data),fall_data,'o',points,interpolation,':.')

    for j = 1:length(fall_data)
        if r10(i) == 0
            if fall_data(j) <= minimum+0.9*magnitude
                r10(i) = j;
            end
        end
        if r50(i) == 0
            if fall_data(j) <= minimum+0.5*magnitude
                r50(i) = j;
            end
        end
```

```
        if r90(i) == 0
            if fall_data(j) <= minimum+0.1*magnitude
                r90(i) = j;
                break
            end
        end
    end
    r10(i) = r10(i)-1; %number of frames
    r50(i) = r50(i)-1;
    r90(i) = r90(i)-1;

end
r10_time = r10/new_fps*1000; %milliseconds
r50_time = r50/new_fps*1000;
r90_time = r90/new_fps*1000;

R10_avg = mean(r10_time);
R50_avg = mean(r50_time);
R90_avg = mean(r90_time);

disp('Time to relaxation, done!');
%% Fmax/F0
Fmax_F0 = [];

for i = 1:size(contr_val,2)
    Fmax_F0(i) = peak_maxes(i)/beat_mean(i);
end

disp('Fmax/F0, done!');

%% Save and export all data

%SAVING OF PARAMETERS
matrix = zeros(size(contr_val,2),9);
Beats = [];
for i = 1:size(contr_val,2)
    a = num2str(i);
    Beats = [Beats "Beat_"+a];
end

matrix(:,1) = Fmax_F0;
matrix(:,2) = time_values(1,:);
matrix(:,3) = c10_time;
matrix(:,4) = c50_time;
matrix(:,5) = c90_time;
matrix(:,6) = r10_time;
```

```
matrix(:,7) = r50_time;
matrix(:,8) = r90_time;
matrix(:,9) = tau;

matrix(end+1,:) = [mean(Fmax_F0) baseline C10_avg C50_avg C90_avg...
    R10_avg R50_avg R90_avg tau_avg];
Beats = [Beats "Average"];

col_names = {'Fmax/F0','Baseline','10% contr [ms]','50% contr [ms]','90% contr [ms]',...
    '10% relax [ms]','50% relax [ms]','90% relax [ms]','tau [ms]'};
matrix = array2table(matrix,'VariableNames',col_names,'RowNames',Beats);

if mkdir('Ca_analysis')

else
    mkdir('Ca_analysis')
end

cd('Ca_analysis')
dir_name = FileName(1:end-4);

if mkdir(dir_name)

else
    mkdir(dir_name)
end

cd(dir_name)
writetable(matrix, [dir_name,'_Ca_Analysis-2.csv'], 'WriteVariableNames',true,...
    'WriteRowNames', true, 'WriteMode', 'overwrite')
%SAVING OF THE PLOTS
intensity_baseline = figure();
plot(intensity)
saveas(intensity_baseline,[dir_name,'_plot.png'])

fprintf('Analysis complete!')
fprintf('All measurements are saved as %s_Ca_analysis.csv\n', dir_name);
%}

%% Functions

function [contr_val, peak_maxes, location_maxes, location_bases, ...
    offset] = contraction_calculator(intensity, NumberOfFrames)

    dif_sig = movmean(diff(intensity),6);
```

```
[~,~,~,Prom] = findpeaks(dif_sig);
[~,location_bases] = findpeaks(dif_sig,'MinPeakProminence',0.5*max(Prom));

[~,~,~,pr2] = findpeaks(intensity);
[peak_maxes,location_maxes] = findpeaks(intensity,'MinPeakProminence',0.5*max(pr2));

if length(location_maxes) < length(location_bases)
   loc_bases = [];
   for i = 1:length(location_maxes)
      temp = location_bases;
      temp_sub = temp-location_maxes(i);

      [~,idx] = min(abs(temp_sub));
      %[~,~,idx] = unique(round(abs(temp_sub-n)),'stable');
      loc_bases(i) = location_bases(idx);
   end
   location_bases = loc_bases;
end


if length(location_maxes)>1

   offset = ceil(0.1*min(diff(location_maxes)));
   dist = max(diff(location_maxes));
   dim = max(dist,NumberOfFrames-location_maxes(end));
   contr_val = zeros(dim,length(location_maxes));

   for j = 1:length(location_maxes)-1
      if location_bases(j)-3*offset >= 1
         start_beat = location_bases(j)-3*offset;
         end_beat = location_bases(j+1) - offset;
         for i = 1:end_beat-start_beat
            contr_val(i,j) = intensity(start_beat+i-1);
         end
      else
         start_beat = 1;
         end_beat = location_bases(j+1) - offset;
         for i = 1:end_beat
            contr_val(i,j) = intensity(start_beat+i-1);
         end
      end
   end
   j = j+1;
else
   j = 1;
   dim = NumberOfFrames-location_maxes(j);
```

```
        offset = ceil(0.1*(min(location_maxes(j),NumberOfFrames-location_maxes(j))));
        contr_val = zeros(dim,j);
    end

    start_beat = location_bases(j)-3*offset;
    end_beat = NumberOfFrames;
    for i = 1:end_beat-start_beat
        contr_val(i,j) = intensity(start_beat+i-1);
    end

end

function [Corrected_Trace] = photobleachcorrection(intensity_trace, contr_val,...
    N_frames)

    BaselineValues = [];
    for K = 1:size(contr_val,2)
        single_trace = nonzeros(contr_val(:,K));
        n = ceil(0.05*length(single_trace));
        peak = max(single_trace);
        Baseline = mean(single_trace(end-n:end));
        Magn = peak - Baseline;
        val95 = Baseline + (0.05 * Magn);
        BaselineValuePts = (single_trace<val95);
        BaselineVals = (single_trace .* BaselineValuePts)';
        BaselineValues = [BaselineValues BaselineVals];
    end

    vs = BaselineValues>0;
    y = 1:size(BaselineValues,2);
    ycords = (vs.*y)';
    BaselineValues = BaselineValues';
    ys = BaselineValues(BaselineValues(:,1) > .0,:);
    xs = ycords(BaselineValues(:,1) > .0,:);

    t = 1:N_frames;
    [pt,st,mut] = polyfit(xs,ys,2);
    trend = polyval(pt,t,[],mut);

    adj = intensity_trace - trend;
    Corrected_Trace =  adj + (intensity_trace(1)-adj(1));

end

function [fitresult, gof,xData, yData] = fit_tau(x, half_v)
```

```
[xData, yData] = prepareCurveData( x, half_v );

ft = fittype( 'exp2' );
opts = fitoptions( 'Method', 'NonlinearLeastSquares' );
opts.Display = 'Off';
opts.Lower = [-Inf -Inf -Inf 0];
opts.Upper = [Inf Inf Inf 0];

[fitresult, gof] = fit( xData, yData, ft, opts );

end

function [C10, C50, C90, t_start] = findcontractionparams(interpolation, peak_int,...
    magnitude)
    C10 = 0;
    C50 = 0;
    C90 = 0;
    t_start = 0;

    for j = 1:length(interpolation)
        if C10 == 0
            if interpolation(j) >= peak_int-0.9*magnitude
                C10 = j;
            end
        elseif C10 ~= 0 && C50 == 0
            if interpolation(j) >= peak_int-0.5*magnitude
                C50 = j;
            end
        elseif C10 ~= 0 && C50 ~= 0 && C90 == 0
            if interpolation(j) >= peak_int-0.1*magnitude
                C90 = j;
            end
        end
    end
    for i = C10:-1:1
        if interpolation(i) <= peak_int-0.95*magnitude
            t_start = i;
            break
        end
    end
end
```

REFERENCES AND NOTES

1. S. S. Chugh, R. Havmoeller, K. Narayanan, D. Singh, M. Rienstra, E. J. Benjamin, R. F. Gillum, Y.H. Kim, J. H. McAnulty Jr, Z.J. Zheng, M. H. Forouzanfar, M. Naghavi, G. A. Mensah, M. Ezzati, C. J.L. Murray, Worldwide epidemiology of atrial fibrillation: A global burden of Disease 2010 Study. *Circulation* **129**, 837–847 (2014).

2. B. P. Krijthe, A. Kunst, E. J. Benjamin, G. Y. H. Lip, O. H. Franco, A. Hofman, J. C. M. Witteman, B. H. Stricker, J. Heeringa, Projections on the number of individuals with atrial fibrillation in the European Union, from 2000 to 2060. *Eur. Heart J.* **34**, 2746–2751 (2013).

3. R. S. Wijesurendra, B. Casadei, Mechanisms of atrial fibrillation. *Heart* **105**, 1860–1867 (2019).

4. O. T. Ly, G. E. Brown, Y. D. Han, D. Darbar, S. R. Khetani, Bioengineering approaches to mature induced pluripotent stem cell-derived atrial cardiomyocytes to model atrial fibrillation. *Exp. Biol. Med.* **246**, 1816–1828 (2021).

5. Y. H. Chen, S.J. Xu, S⁚ Bendahhou, X.L. Wang, Y. Wang, W.Y. Xu, H.W. Jin, H. Sun, X.Y. Su, Q.N. Zhuang, Y.Q. Yang, Y.B. Li, Y. Liu, H.J. Xu, X.F. Li, N. Ma, C.P. Mou, Z. Chen, J. Barhanin, W. Huang, KCNQ1 gain-of-function mutation in familial atrial fibrillation. *Science* **299**, 251–254 (2003).

6. S. Ghezelbash, C. E. Molina, D. Dobrev, Altered atrial metabolism: An underappreciated contributor to the initiation and progression of atrial fibrillation. *J. Am. Heart Assoc.* **4**, e001808 (2015).

7. T. Christ, M. D. Lemoine, T. Eschenhagen, Are atrial human pluripotent stem cell-derived cardiomyocytes ready to identify drugs that beat atrial fibrillation? *Nat. Commun.* **12**, 1725 (2021).

8. I. Goldfracht, Y. Efraim, R. Shinnawi, E. Kovalev, I. Huber, A. Gepstein, G. Arbel, N. Shaheen, M. Tiburcy, W. H. Zimmermann, M. Machluf, L. Gepstein, Engineered heart tissue

models from hiPSC-derived cardiomyocytes and cardiac ECM for disease modeling and drug testing applications. *Acta Biomater.* **92**, 145–159 (2019).

9. O. Bergmann, S. Zdunek, A. Felker, M. Salehpour, K. Alkass, S. Bernard, S. L. Sjostrom, M. Szewczykowska, T. Jackowska, C. dos Remedios, T. Malm, M. Andrä, R. Jashari, J. R. Nyengaard, G. Possnert, S. Jovinge, H. Druid, J. Frisén, Dynamics of cell generation and turnover in the human heart. *Cell* **161**, 1566–1575 (2015).

10. C. A. Souders, S. L. Bowers, T. A. Baudino, Cardiac fibroblast: The renaissance cell. *Circ. Res.* **105**, 1164–1176 (2009).

11. M. Litviňuková, C. Talavera-López, H. Maatz, D. Reichart, C. L. Worth, E. L. Lindberg, M. Kanda, K. Polanski, M. Heinig, M. Lee, E. R. Nadelmann, K. Roberts, L. Tuck, E. S. Fasouli, D. M. De Laughter, B. M. Donough, H. Wakimoto, J. M. Gorham, S. Samari, K. T. Mahbubani, K. Saeb-Parsy, G. Patone, J. J. Boyle, H. Zhang, H. Zhang, A. Viveiros, G. Y. Oudit, O. A. Bayraktar, J G Seidman, C. E. Seidman, M. Noseda, N. Hubner, S. A. Teichmann, Cells of the adult human heart. *Nature* **588**, 466–472 (2020).

12. E. Ongstad, P. Kohl, Fibroblast-myocyte coupling in the heart: Potential relevance for therapeutic interventions. *J. Mol. Cell. Cardiol.* **91**, 238–246 (2016).

13. P. Beauchamp, C. B. Jackson, L. C. Ozhathil, I. Agarkova, C. L. Galindo, D. B. Sawyer, T. M. Suter, C. Zuppinger, 3D coculture of hiPSC-derived cardiomyocytes with cardiac fibroblasts improves tissue-like features of cardiac spheroids. *Front. Mol. Biosci.* **7**, 14 (2020).

14. E. Giacomelli, V. Meraviglia, G. Campostrini, A. Cochrane, X. Cao, R.W.J. van Helden, A. Krotenberg Garcia, M. Mircea, S. Kostidis, R.P. Davis, B.J. van Meer, C.R. Jost, A.J. Koster, H. Mei, D.G. Míguez, A.A. Mulder, M. Ledesma-Terrón, G. Pompilio, L. Sala, D.C.F. Salvatori, R.C. Slieker, E. Sommariva, A.A.F. de Vries, M. Giera, S. Semrau, L.G.J. Tertoolen, V.V. Orlova, M. Bellin, C.L. Mummery, Human-iPSC-derived cardiac stromal cells enhance maturation in 3D cardiac microtissues and reveal non-cardiomyocyte contributions to heart disease. *Cell Stem Cell* **26**, 862–879.e11 (2020).

15. T. A. Hookway, O. B. Matthys, F. N. Mendoza-Camacho, S. Rains, J. E. Sepulveda, D. A. Joy, T. C. McDevitt, Phenotypic variation between stromal cells differentially impacts engineered cardiac tissue function. *Tissue Eng. Part A* **25**, 773–785 (2019).

16. Y. Guo, Y. Cao, B. D. Jardin, I. Sethi, Q. Ma, B. Moghadaszadeh, E. C. Troiano, N. Mazumdar, M. A. Trembley, E. M. Small, G.-C. Yuan, A. H. Beggs, W. T. Pu, Sarcomeres regulate murine cardiomyocyte maturation through MRTF-SRF signaling. *Proc. Natl. Acad. Sci. U.S.A.* **118**, e2008861118 (2021).

17. Y. Guo, W. T. Pu, Cardiomyocyte Maturation: New Phase in Development. *Circ. Res.* **126**, 1086–1106 (2020).

18. A. J. S. Ribeiro, Y.S. Ang, J.D. Fu, R. N. Rivas, T. M. A. Mohamed, G. C. Higgs, D. Srivastava, B. L. Pruitt, Contractility of single cardiomyocytes differentiated from pluripotent stem cells depends on physiological shape and substrate stiffness. *Proc. Natl. Acad. Sci. U.S.A.* **112**, 12705–12710 (2015).

19. S. D. Burnett, A. D. Blanchette, W. A. Chiu, I. Rusyn, Cardiotoxicity hazard and risk characterization of toxcast chemicals using human induced pluripotent stem cell-derived cardiomyocytes from multiple donors. *Chem. Res. Toxicol.* **34**, 2110–2124 (2021).

20. F. A. Grimm, A. Blanchette, J.S. House, K. Ferguson, N.H. Hsieh, C. Dalaijamts, A.A. Wright, B. Anson, F.A. Wright, W.A. Chiu, I. Rusyn, A human population-based organotypic in vitro model for cardiotoxicity screening. *ALTEX* **35**, 441–452 (2018).

21. F. Rizvi, A. DeFranco, R. Siddiqui, U. Negmadjanov, L. Emelyanova, A. Holmuhamedov, G. Ross, Y. Shi, E. Holmuhamedov, D. Kress, A. J. Tajik, A. Jahangir, Chamber-specific differences in human cardiac fibroblast proliferation and responsiveness toward simvastatin. *Am. J. Physiol. Cell Physiol.* **311**, C330–339 (2016).

22. S. T. Brinker, S. P. Kearney, T. J. Royston, D. Klatt, Simultaneous magnetic resonance and optical elastography acquisitions: Comparison of displacement images and shear modulus

estimations using a single vibration source. *J. Mech. Behav. Biomed. Mater.* **84**, 135–144 (2018).

23. P. Kohl, P. Camelliti, Fibroblast-myocyte connections in the heart. *Heart Rhythm* **9**, 461–464 (2012).

24. G. Genet, C. Guilbeau-Frugier, B. Honton, E. Dague, M. D. Schneider, C. Coatrieux, D. Calise, C. Cardin, C. Nieto, B. Payré, C. Dubroca, P. Marck, C. Heymes, A. Dubrac, D. Arvanitis, F. Despas, M.F. Altié, M.H. Seguelas, M.B. Delisle, A. Davy, J.M. Sénard, A. Pathak, C. Galés, Ephrin-B1 is a novel specific component of the lateral membrane of the cardiomyocyte and is essential for the stability of cardiac tissue architecture cohesion. *Circ. Res.* **110**, 688–700 (2012).

25. J. Lewandowski, N. Rozwadowska, T. J. Kolanowski, A. Malcher, A. Zimna, A. Rugowska, K. Fiedorowicz, W. Łabędź, Ł. Kubaszewski, K. Chojnacka, K. Bednarek-Rajewska, P. Majewski, M. Kurpisz, The impact of in vitro cell culture duration on the maturation of human cardiomyocytes derived from induced pluripotent stem cells of myogenic origin. *Cell Transplant.* **27**, 1047–1067 (2018).

26. N. Voigt, J. Heijman, Q. Wang, D. Y. Chiang, N. Li, M. Karck, X. H.T. Wehrens, S. Nattel, D. Dobrev, Cellular and molecular mechanisms of atrial arrhythmogenesis in patients with paroxysmal atrial fibrillation. *Circulation* **129**, 145–156 (2014).

27. N. Huebsch, B. Charrez, G. Neiman, B. Siemons, S. C. Boggess, S. Wall, V. Charwat, K. H. Jæger, D. Cleres, Å. Telle, F. T. Lee-Montiel, N. C. Jeffreys, N. Deveshwar, A. G. Edwards, J. Serrano, M. Snuderl, A. Stahl, A. Tveito, E. W. Miller, K. E. Healy, Metabolically driven maturation of human-induced-pluripotent-stem-cell-derived cardiac microtissues on microfluidic chips. *Nat. Biomed. Eng.* **6**, 372–388 (2022).

28. A. J. S. Ribeiro, *O. Schwab, M. A. Mandegar, Y.S. Ang, B. R. Conklin, D. Srivastava, B. L. Pruitt*, Multi-imaging method to assay the contractile mechanical output of micropatterned human iPSC-derived cardiac myocytes. *Circ. Res.* **120**, 1572–1583 (2017).

29. K. Ronaldson-Bouchard, S. P. Ma, K. Yeager, T. Chen, L.J. Song, D. Sirabella, K. Morikawa, D. Teles, M. Yazawa, G. Vunjak-Novakovic, Advanced maturation of human cardiac tissue grown from pluripotent stem cells. *Nature* **556**, 239–243 (2018).

30. E. Karbassi, A. Fenix, S. Marchiano, N. Muraoka, K. Nakamura, X. Yang, C. E. Murry, Cardiomyocyte maturation: advances in knowledge and implications for regenerative medicine. *Nat. Rev. Cardiol.* **17**, 341–359 (2020).

31. A. Segret, C. Rücker-Martin, C. Pavoine, J. Flavigny, E. Deroubaix, M.A. Châtel, A. Lombet, J.F. Renaud, Structural localization and expression of CXCL12 and CXCR4 in rat heart and isolated cardiac myocytes. *J. Histochem. Cytochem.* **55**, 141–150 (2007).

32. N. Bildyug, Extracellular matrix in regulation of contractile system in cardiomyocytes. *Int. J. Mol. Sci.* **20**, 5054 (2019).

33. M. W. Szymanski, D. P. Singh, in *StatPearls* (2022).

34. W. S. Redfern, L. Carlsson, A.S. Davis, W.G. Lynch, I. MacKenzie, S. Palethorpe, P.K. Siegl, I. Strang, A.T. Sullivan, R. Wallis, A.J. Camm, T.G. Hammond, Relationships between preclinical cardiac electrophysiology, clinical QT interval prolongation and torsade de pointes for a broad range of drugs: evidence for a provisional safety margin in drug development. *Cardiovasc. Res.* **58**, 32–45 (2003).

35. J. P. Mounsey, J. P. DiMarco, Cardiovascular drugs Dofetilide. *Circulation* **102**, 2665–2670 (2000).

36. E. Aliot, A. Capucci, H. J. Crijns, A. Goette, J. Tamargo, Twenty-five years in the making: Flecainide is safe and effective for the management of atrial fibrillation. *Europace* **13**, 161–173 (2011).

37. D. Melgari, Y. Zhang, A. El Harchi, C. E. Dempsey, J. C. Hancox, Molecular basis of hERG potassium channel blockade by the class Ic antiarrhythmic flecainide. *J. Mol. Cell. Cardiol.* **86**, 42–53 (2015).

38. L. Hong, M. Zhang, O. T. Ly, H. Chen, A. Sridhar, E. Lambers, B. Chalazan, S.W. Youn, M. Maienschein-Cline, L. Feferman, S.G. Ong, J. C. Wu, J. Rehman, D. Darbar, Human induced pluripotent stem cell-derived atrial cardiomyocytes carrying an *SCN5A* mutation identify nitric oxide signaling as a mediator of atrial fibrillation. *Stem Cell Reports* **16**, 1542–1554 (2021).

39. U.S. Food and Drug Administration, *RANEXA (ranolazine) extended-release tablets NDA 21-526/S-002 approval letter* (NDA 21-526/S-002, FDA, 2007); www.accessdata.fda.gov/drugsatfda_docs/label/2007/021526s002lbl.pdf).

40. *A. J. S. Ribeiro, B. D. Guth, M. Engwall, S. Eldridge, C. M. Foley, L. Guo, G. Gintant, J. Koerner, S. T. Parish, J. B. Pierson, M. Brock, K. W. Chaudhary, Y. Kanda, B. Berridge,* Considerations for an in vitro, cell-based testing platform for detection of drug-induced inotropic effects in early drug development. Part 2: Designing and fabricating microsystems for assaying cardiac contractility with physiological relevance using human ipsc-cardiomyocytes. *Front. Pharmacol.* **10**, 934 (2019).

41. P. Liang, F. Lan, A. S. Lee, T. Gong, V. Sanchez-Freire, Y. Wang, S. Diecke, K. Sallam, J. W. Knowles, P. J. Wang, P. K. Nguyen, D. M. Bers, R. C. Robbins, J. C. Wu, Drug screening using a library of human induced pluripotent stem cell-derived cardiomyocytes reveals disease-specific patterns of cardiotoxicity. *Circulation* **127**, 1677–1691 (2013).

42. S. B. Bremner, K. S. Gaffney, N. J. Sniadecki, D. L. Mack, A change of heart: Human cardiac tissue engineering as a platform for drug development. *Curr. Cardiol. Rep.* **24**, 473–486 (2022).

43. C. Zuppinger, 3D cardiac cell culture: A critical review of current technologies and applications. *Front. Cardiovasc. Med.* **6**, 87 (2019).

44. L. C. Liew, B. X. Ho, B. S. Soh, Mending a broken heart: Current strategies and limitations of cell-based therapy. *Stem Cell Res. Ther.* **11**, 138 (2020).

45. C. Zuppinger, 3D culture for cardiac cells. *BBA-Mol. Cell. Res.* **1863**, 1873–1881 (2016).

46. B. N. Napiwocki, A. Stempien, D. Lang, R.A. Kruepke, G. Kim, J. Zhang, L.L. Eckhardt, A.V. Glukhov, T.J. Kamp, W.C. Crone, Micropattern platform promotes extracellular matrix remodeling by human PSC-derived cardiac fibroblasts and enhances contractility of co-cultured cardiomyocytes. *Physiol. Rep.* **9**, e15045 (2021).

47. I. Batalov, Q. Jallerat, S. Kim, J. Bliley, A. W. Feinberg, Engineering aligned human cardiac muscle using developmentally inspired fibronectin micropatterns. *Sci. Rep.* **11**, 11502 (2021).

48. B. N. Napiwocki, D. Lang, A. Stempien, J. Zhang, R. Vaidyanathan, J. C. Makielski, L. L. Eckhardt, A. V. Glukhov, T. J. Kamp, W. C. Crone, Aligned human cardiac syncytium for in vitro analysis of electrical, structural, and mechanical readouts. *Biotechnol. Bioeng.* **118**, 442–452 (2021).

49. P. Ahuja, P. Sdek, W. R. MacLellan, Cardiac myocyte cell cycle control in development, disease, and regeneration. *Physiol. Rev.* **87**, 521–544 (2007).

50. T. Smit, E. Schickel, O. Azimzadeh, C. von Toerne, O. Rauh, S. Ritter, M. Durante, I. S. Schroeder, A Human 3D cardiomyocyte risk model to study the cardiotoxic influence of x-rays and other noxae in adults. *Cell* **10**, 2608 (2021).

51. D. Frank, C. Kuhn, B. Brors, C. Hanselmann, M. Lüdde, H. A. Katus, N. Frey, Gene expression pattern in biomechanically stretched cardiomyocytes—Evidence for a stretch-specific gene program. *Hypertension* **51**, 309–318 (2008).

52. A. K. Peter, H. Cheng, R. S. Ross, K. U. Knowlton, J. Chen, The costamere bridges sarcomeres to the sarcolemma in striated muscle. *Prog. Pediatr. Cardiol.* **31**, 83–88 (2011).

53. T. J. Herron, A. M. D. Rocha, K. F. Campbell, D. Ponce-Balbuena, B. C. Willis, G. Guerrero-Serna, Q. Liu, M. Klos, H. Musa, M. Zarzoso, A. Bizy, J. Furness, J. Anumonwo, S. Mironov, J. Jalife, Extracellular matrix-mediated maturation of human pluripotent stem cell-derived cardiac monolayer structure and electrophysiological function. *Circ. Arrhythm. Electrophysiol.* **9**, e003638 (2016).

54. O. T. Ly, H. Chen, G. E. Brown, L. Hong, X. Wang, Y. D. Han, M. A. Pavel, A. Sridhar, M. Maienschein-Cline, B. Chalazan, S.G. Ong, K. Abdelhady, M. Massad, L. E. Rizkallah, J. Rehman, S. R. Khetani, D. Darbar, Mutant ANP induces mitochondrial and ion channel remodeling in a human iPSC-derived atrial fibrillation model. *JCI Insight* **7**, e155640 (2022).

55. A. S. T. Smith, E. Choi, K. Gray, J. Macadangdang, E. H. Ahn, E. C. Clark, M. A. Laflamme, J. C. Wu, C. E. Murry, L. Tung, D.H. Kim, NanoMEA: A tool for high-throughput, electrophysiological phenotyping of patterned excitable cells. *Nano Lett.* **20**, 1561–1570 (2020).

56. B. Liau, C. P. Jackman, Y. Li, N. Bursac, Developmental stage-dependent effects of cardiac fibroblasts on function of stem cell-derived engineered cardiac tissues. *Sci. Rep.* **7**, 42290 (2017).

57. H. Zhang, L. Tian, M. Shen, C. Tu, H. Wu, M. Gu, D. T. Paik, J. C. Wu, Generation of Quiescent Cardiac Fibroblasts From Human Induced Pluripotent Stem Cells for In Vitro Modeling of Cardiac Fibrosis. *Circ. Res.* **125**, 552–566 (2019).

58. E. E. Hui, S. N. Bhatia, Micromechanical control of cell-cell interactions. *Proc. Natl. Acad. Sci. U.S.A.* **104**, 5722–5726 (2007).

59. M. D. Davidson, S. R. Khetani, Intermittent starvation extends the functional lifetime of primary human hepatocyte cultures. *Toxicol. Sci.* **174**, 266–277 (2020).

60. M. Argenziano, E. Lambers, L. Hong, A. Sridhar, M. Zhang, B. Chalazan, A. Menon, E. Savio-Galimberti, J. C. Wu, J. Rehman, D. Darbar, Electrophysiologic characterization of calcium handling in human induced pluripotent stem cell-derived atrial cardiomyocytes. *Stem Cell Reports.* **10**, 1867–1878 (2018).

61. A. Sharma, G. Li, K. Rajarajan, R. Hamaguchi, P.W. Burridge, S.M. Wu, Derivation of highly purified cardiomyocytes from human induced pluripotent stem cells using small molecule-modulated differentiation and subsequent glucose starvation. *J. Vis. Exp.*, 52628 (2015).

62. N. Majoul, S. Aouida, B. Bessais, Progress of porous silicon APTES-functionalization by FTIR investigations. *Appl. Surf. Sci.* **331**, 388–391 (2015).

63. X. Wang, Y. Gu, S. Zhang, G. Li, T. Liu, T. Wang, H. Qin, B. Jiang, L. Zhu, Y. Li, H. Lei, M. Li, Q. Zhang, R. Yang, F. Fang, H. Guo, Unbiased enrichment of urine exfoliated cells on nanostructured substrates for sensitive detection of urothelial tumor cells. *Cancer Med.* **9**, 290–301 (2020).

64. S. H. North, E. H. Lock, C. J. Cooper, J. B. Franek, C. R. Taitt, S. G. Walton, Plasma-based surface modification of polystyrene microtiter plates for covalent immobilization of biomolecules. *ACS Appl. Mater. Interfaces* **2**, 2884–2891 (2010).

65. D. Pesen, W. F. Heinz, J. L. Werbin, J. H. Hoh, D. B. Haviland, Electron beam patterning of fibronectin nanodots that support focal adhesion formation. *Soft Matter* **3**, 1280–1284 (2007).

66. M. J. Birket, M. C. Ribeiro, G. Kosmidis, D. Ward, A. R. Leitoguinho, V. van de Pol, C. Dambrot, H. D. Devalla, R. P. Davis, P. G. Mastroberardino, D. E. Atsma, R. Passier, C. L. Mummery, Contractile defect caused by mutation in MYBPC3 revealed under conditions optimized for human PSC-cardiomyocyte function. *Cell Rep.* **13**, 733–745 (2015).

67. S. M. Biendarra-Tiegs, D. J. Clemens, F. J. Secreto, T. J. Nelson, human induced pluripotent stem cell-derived non-cardiomyocytes modulate cardiac electrophysiological maturation through connexin 43-mediated cell-cell interactions. *Stem Cells Dev.* **29**, 75–89 (2020).

68. D. Torre, A. Lachmann, A. Ma'ayan, BioJupies: Automated generation of interactive notebooks for RNA-seq data analysis in the cloud. *Cell Syst.* **7**, 556–561.e3 (2018).

69. M. D. Robinson, D. J. McCarthy, G. K. Smyth, edgeR: A Bioconductor package for differential expression analysis of digital gene expression data. *Bioinformatics* **26**, 139–140 (2010).

70. G. Bindea, B. Mlecnik, H. Hackl, P. Charoentong, M. Tosolini, A. Kirilovsky, W.H. Fridman, F. Pagès, Z. Trajanoski, J. Galon, ClueGO: A Cytoscape plug-in to decipher

functionally grouped gene ontology and pathway annotation networks. *Bioinformatics* **25**, 1091–1093 (2009).

71. I. Kopljar, H. R. Lu, K. van Ammel, M. Otava, F. Tekle, A. Teisman, D. J. Gallacher, Development of a human iPSC cardiomyocyte-based scoring system for cardiac hazard identification in early drug safety de-risking. *Stem Cell Reports* **11**, 1365–1377 (2018).

72. R. J. Mills, D.M. Titmarsh, X. Koenig, B.L. Parker, J.G. Ryall, G.A. Quaife-Ryan, H.K. Voges, M.P. Hodson, C. Ferguson, L. Drowley, A.T. Plowright, E.J. Needham, Q.D. Wang, P. Gregorevic, M. Xin, W.G. Thomas, R.G. Parton, L.K. Nielsen, B.S. Launikonis, D.E. James, D.A. Elliott, E.R. Porrello, J.E. Hudson, Functional screening in human cardiac organoids reveals a metabolic mechanism for cardiomyocyte cell cycle arrest. *Proc. Natl. Acad. Sci. U.S.A.* **114**, E8372-E8381 (2017).

73. D. Darbar, P. J. Kannankeril, B. S. Donahue, G. Kucera, T. Stubblefield, J. L. Haines, A. L. George Jr, D. M. Roden, Cardiac sodium channel (SCN5A) variants associated with atrial fibrillation. *Circulation* **117**, 1927–1935 (2008).

74. G. Olivetti, E. Cigola, R. Maestri, D. Corradi, C. Lagrasta, S.R. Gambert, P. Anversa, Aging, cardiac hypertrophy and ischemic cardiomyopathy do not affect the proportion of mononucleated and multinucleated myocytes in the human heart. *J. Mol. Cell. Cardiol.* **28**, 1463–1477 (1996).

75. S. D. Lundy, W. Z. Zhu, M. Regnier, M. A. Laflamme, Structural and functional maturation of cardiomyocytes derived from human pluripotent stem cells. *Stem Cells Dev.* **22**, 1991–2002 (2013).

76. Z. Onódi, T. Visnovitz, B. Kiss, S. Hambalkó, A. Koncz, B. Ágg, B. Váradi, V. É. Tóth, R. N. Nagy, T. G. Gergely, D. Gergő, A. Makkos, C. Pelyhe, N. Varga, D. Reé, Á. Apáti, P. Leszek, T. Kovács, N. Nagy, P. Ferdinandy, E. I. Buzás, A. Görbe, Z. Giricz, Z. V. Varga, Systematic transcriptomic and phenotypic characterization of human and murine cardiac myocyte cell lines and primary cardiomyocytes reveals serious limitations and low resemblances to adult cardiac phenotype. *J. Mol. Cell. Cardiol.* **165**, 19–30 (2022).

77. B. Emde, A. Heinen, A. Gadecke, K. Bottermann, Wheat germ agglutinin staining as a suitable method for detection and quantification of fibrosis in cardiac tissue after myocardial infarction. *Eur. J. Histochem.* **58**, 2448 (2014).

78. Y. Psaras, F. Margara, M. Cicconet, A. J. Sparrow, G. G. Repetti, M. Schmid, V. Steeples, J. A.L. Wilcox, A. Bueno-Orovio, C. S. Redwood, H. C. Watkins, P. Robinson, B. Rodriguez, J. G. Seidman, C. E. Seidman, C. N. Toepfer, CalTrack: High-throughput automated calcium transient analysis in cardiomyocytes. *Circ. Res.* **129**, 326–341 (2021).

79. A. Kumar, Y. Wu, R. Christensen, P. Chandris, W. Gandler, E. McCreedy, A. Bokinsky, D. A. Colón-Ramos, Z. Bao, M. McAuliffe, G. Rondeau, H. Shroff, Dual-view plane illumination microscopy for rapid and spatially isotropic imaging. *Nat. Protoc.* **9**, 2555–2573 (2014).

80. L. M. Swift, H. Asfour, N. G. Posnack, A. Arutunyan, M. W. Kay, N. Sarvazyan, Properties of blebbistatin for cardiac optical mapping and other imaging applications. *Pflugers Arch.* **464**, 503–512 (2012).
